# Development, synthesis and validation of improved c‐Myc/Max inhibitors

**DOI:** 10.1111/jcmm.18272

**Published:** 2024-04-03

**Authors:** Sümbül Yıldırım, Fatih Kocabaş, Arif Mermer

**Affiliations:** ^1^ Department of Genetics and Bioengineering, Faculty of Engineering Yeditepe University Istanbul Turkey; ^2^ Graduate School of Natural and Applied Sciences Yeditepe University Istanbul Turkey; ^3^ Institute for Diabetes and Cancer Helmholtz Diabetes Center, Helmholtz Center Neuherberg Germany; ^4^ Department of Biotechnology University of Health Sciences Istanbul Turkey; ^5^ Experimental Medicine Application and Research Center University of Health Sciences Istanbul Turkey; ^6^ UR22722, LABCİS, Faculty of Science and Technology University of Limoges Limoges France

**Keywords:** anti‐cancer, c‐Myc, Max, rhodanine, ultrasonication

## Abstract

The pathophysiological foundations of various diseases are often subject to alteration through the utilization of small compounds, rendering them invaluable tools for the exploration and advancement of novel therapeutic strategies. Within the scope of this study, we meticulously curated a diverse library of novel small compounds meticulously designed to specifically target the c‐Myc/Max complex. We conducted in vitro examinations of novel c‐Myc inhibitors across a spectrum of cancer cell lines, including PANC1 (pancreatic adenocarcinoma), MCF7 (breast carcinoma), DU‐145 (prostate carcinoma), and A549 (lung cancer). The initial analysis involved a 25 μM dose, which enabled the identification of potent anticancer compounds effective against a variety of tumour types. We identified c‐Myc inhibitors with remarkable potency, featuring IC50 values as low as 1.6 μM and up to 40 times more effective than the reference molecule in diminishing cancer cell viability. Notably, c‐Myc‐i7 exhibited exceptional selectivity, displaying 37‐fold and 59‐fold preference for targeting prostate and breast cancers, respectively, over healthy cells. Additionally, we constructed drug‐likeness models. This study underscores the potential for in vitro investigations of various tumour types using novel c‐Myc inhibitors to yield ground‐breaking and efficacious anticancer compounds.

## 1 INTRODUCTION

Cancer comprises a diverse group of diseases characterized by the uncontrolled growth and division of abnormal cells, culminating in the formation of malignant tumours. The potential for cancer to metastasize, or spread to other parts of the body, presents significant health risks. The choice of cancer treatment depends on factors such as the specific type and stage of the disease, as well as the patient's age and overall health. Common treatment modalities include chemotherapy, radiation therapy and surgical intervention.^1^ Continuous advancements in medical science led to the development of novel medications and therapies, aimed at improving patient outcomes while mitigating adverse effects.

Within the realm of cancer biology, the MYC paralogs, including C‐MYC, N‐MYC and L‐MYC, are frequently observed to be overexpressed in various cancer types.^2^, ^3^ This overexpression drives unregulated cell proliferation and abnormal cellular growth. Such dysregulation can result in tumour enlargement, invasiveness, and the potential for metastasis. Additionally, MYC paralogs contribute to the development of drug resistance in cancer cells by upregulating genes associated with resistance to therapeutic agents.^4^ Consequently, this heightened resistance increases the risk of cancer recurrence and diminishes overall survival rates. C‐Myc is a transcription factor that plays a pivotal role in regulating cell growth, proliferation, and apoptosis.^5^ Its overexpression is a common hallmark in numerous cancer types and is implicated in tumorigenesis. C‐Myc exerts control over the progression of the cell cycle, promoting cell proliferation and heightening the risk of tumorigenesis.^6^

Efforts to enhance cancer treatment and prevention include the development of new medications targeting C‐Myc.^7^ One promising approach involves inhibiting C‐Myc activity, thereby curtailing the proliferation and growth of cancer cells. Inhibition of C‐Myc has been under investigation as a potential therapeutic strategy for various cancers, including breast, ovarian, lung and prostate cancers.

Small molecule c‐Myc inhibitors are compounds designed to specifically hinder the activity of the c‐Myc protein. Typically, these inhibitors are small organic molecules capable of binding to the c‐Myc protein, thus preventing its interaction with DNA and the execution of its normal cellular functions. One notable example is OmoMYC, which effectively inhibits all three variants of MYC from binding to their target promoters.^8^ OmoMYC has demonstrated promising anticancer potential in various preclinical models with minimal side effects. Its broad effectiveness and low toxicity have prompted its development for clinical studies. While no medication directly targeting MYC has been tested in humans, APTO‐253, which partly reduces MYC expression, is currently undergoing phase I clinical trials in patients with relapsed/refractory acute myeloid leukaemia or myelodysplastic syndrome.^7^ It's worth noting that OmoMYC, being a peptide/small protein, may be susceptible to in vivo degradation. Although other small molecules like 10058‐F4, 10074‐G5, KJ‐Pyr‐9, MYCMI‐6, MYCi361 and MYCi975 also inhibit Myc–Max interaction, their activity is limited to specific cancer types and has been associated with side effects.^9^, ^10^, ^11^, ^12^, ^13^, ^14^ Therefore, there is a growing demand for novel c‐Myc inhibitors capable of targeting a broader spectrum of cancers and facilitating combination therapies. Such inhibitors hold the potential to address diseases characterized by aberrant c‐Myc activity, including certain autoimmune disorders, while also enhancing the efficacy of existing cancer treatments such as chemotherapy and radiation.^15^, ^16^, ^17^, ^18^ Ultimately, the development of innovative c‐Myc inhibitors holds promise for more effective treatments across a range of diseases.

In this study, we synthesized novel rhodanine derivatives utilizing ultrasonication, an environmentally friendly green chemistry technique, through a one‐pot four‐component reaction, achieving exceptional yields. The resulting compounds were meticulously characterized and further optimized as potent c‐Myc/Max inhibitors, employing both in silico and in vitro methodologies. Assessment of these compounds across a spectrum of cancer types, alongside healthy cell lines, revealed their efficacy and enhanced drug‐like properties.

## 2 MATERIALS and METHODS

FTIR (Fourier Transform Infrared Spektrofotometre) spectra were recorded using a ThermoFisher Scientific Nicolet IS50 FTIR spectrometer. ^1^H NMR and ^13^C NMR (APT) spectra were recorded on Bruker Avance II 400 MHz NMR spectrometer (chemical shift in ppm downfield from TMS (tetramethylsilane) as an internal reference). The mass spectra were obtained at MALDI‐TOF/MS (Bruker‐UltrafleXtreme). The reactions were monitored by TLC using silica gel coated plates and different solvents solutions as the mobile phase. The synthesis of the reaction was carried out in the ISOLAB Ultrasonic bath. Commercial grade reagents were purchased from Alfa Aesar (Kandel, Germany) and used without further purification.

### 2.1 Experimental

#### 2.1.1 General method for synthesis of compounds 5a‐n

The mixture of carbon disulfide (20 mmol), 3, 5‐Bis (trifluoromethyl) benzylamine (10 mmol), and ethyl bromoacetate (10 mmol) in water (1 mL) was sonicated at rt, 40 Hz for 2 min. To this mixture, triethylamine (30 mmol) and substituted aromatic aldehyde (10 mmol) were added and stirred for an additional 5 min. Then, 10 mL water was added to the mixture and sonicated 1 min more. After completion of the reaction reaction progress was monitored by TLC (CHCl_3_: EtOH [4.5:0.5]), 50 mL of water was added and extracted with CHCl_3_ (3 × 10 mL). The combined organic phase was dried over MgSO_4_, and the solvent was removed under reduced pressure to dryness. The crude product was purified by recrystallization from an appropriate solvent.

(*Z*)‐3‐(3,5‐bis(trifluoromethyl)benzyl)‐5‐(4‐nitrobenzylidene)‐2‐thioxothiazolidin‐4‐one (**5a**)

Recrystallized from ethanol. Yield: 91%. FT‐IR (cm^−1^): 3096 (arH), 1714 (C=O), 1598 (C=N), 1530 (NO_2_), 1184 (C=S). ^1^H NMR (400 MHz, DMSO‐*d*_6_) *δ* (ppm): 5.40 (s, 2H, CH_2_), 7.83 (t, 1H, *J* = 8.0 Hz, arH), 8.02 (s, 1H, arH), 8.03 (bs, 2H, arH), 8.06 (s, 2H, arH), 8.31 (dd, 1H, *J*_*1*_ = 2.0 Hz, *J*_*2*_ = 2.0 Hz, arH), 8.50 (s, 1H, CH). ^13^C NMR (APT) (100 MHz, DMSO‐*d*_6_) *δ* (ppm): 47.03, 122.07–122.11 (d, *J* = 4.0 Hz), 122.28, 124.99, 125.35, 125.51, 126.28, 129.36–129.41 (d, *J* = 5.0 Hz), 130.56, 130.88, 131.02, 131.57, 136.14, 138.56, 148.80, 167.51, 194.02. MALDI‐TOF/MS (*m*/*z*): Calculated: 492.00370; Found: 492.99400.

(*Z*)‐3‐(3,5‐bis(trifluoromethyl)benzyl)‐5‐(3,5‐dimethylbenzylidene)‐2‐thioxothiazolidin‐4‐one (**5b**)

Recrystallized from methanol. Yield: 87%. FT‐IR (cm^−1^): 3066 (arH), 1715 (C=O), 1608 (C=N), 1168 (C=S). ^1^H NMR (400 MHz, DMSO‐*d*_6_) *δ* (ppm): 2.32 (s, 6H, 2CH_3_), 5.38 (s, 2H, CH_2_), 7.15 (s, 1H, arH), 7.22 (s, 2H, arH), 7.73 (s, 1H, arH), 8.03 (s, 3H, arH + CH). ^13^C NMR (APT) (100 MHz, DMSO‐*d*_6_) *δ* (ppm): 21.22, 46.89, 122.03, 122.27, 122.67, 124.98, 127.63, 127.70, 128.78, 129.33, 130.57, 130.90, 133.19, 133.39, 134.07, 138.70, 139.25, 167.64, 194.55. MALDI‐TOF/MS (*m*/*z*): Calculated: 475.04992; Found: 475.03320.

(*Z*)‐3‐(3,5‐bis(trifluoromethyl)benzyl)‐5‐(3‐chlor‐4‐fluorobenzylidene)‐2‐thioxothiazolidin‐4‐one (**5c**)

Recrystallized from ethanol. Yield: 88%. FT‐IR (cm^−1^): 3068 (arH), 1719 (C=O), 1608 (C=N), 1166 (C=S). ^1^H NMR (400 MHz, DMSO‐*d*_6_) *δ* (ppm): 5.38 (s, 2H, CH_2_), 7.58 (d, 1H, *J* = 8.0 Hz, arH), 7.61–7.63 (m, 1H, arH), 7.83 (s, 1H, arH), 7.90 (dd, 1H, *J*_*1*_ = 2.0 Hz, *J*_*2*_ = 2.0 Hz, arH), 8.01 (s, 1H, CH), 8.03 (s, 2H, arH). ^13^C NMR (APT) (100 MHz, DMSO‐*d*_6_) *δ* (ppm): 21.22, 46.95, 118.10, 118.71, 121.19, 121.37, 122.05, 122.25, 124.53, 124.96, 129.34, 130.24–131.22, 131.03, 131.11, 131.48, 133.64, 138.57, 157.29–159.81 (d_C‐F_, *J* = 252.0 Hz), 167.51, 194.06. MALDI‐TOF/MS (*m*/*z*): Calculated: 498.97023; Found: 499.97361.

(*Z*)‐3‐(3,5‐bis(trifluoromethyl)benzyl)‐5‐(3,4‐dimethoxybenzylidene)‐2‐thioxothiazolidin‐4‐one (**5d**)

Recrystallized from ethanol. Yield: 93%. FT‐IR (cm^−1^): 3019 (arH), 1716 (C=O), 1588 (C=N), 1168 (C=S). ^1^H NMR (400 MHz, DMSO‐*d*_6_) *δ* (ppm): 3.83 (s, 6H, 2OCH_3_), 5.39 (s, 2H, CH_2_), 7.15 (s, 1H, arH), 7.23 (bs, 2H, arH), 7.82 (s, 1H, arH), 8.03 (bs, 3H, arH + CH). ^13^C NMR (APT) (100 MHz, DMSO‐*d*_6_) *δ* (ppm): 46.85, 53.06, 56.24, 112.75, 114.09, 119.79, 122.07, 122.27, 124.99, 125.41, 126.13, 129.24–129.28 (d, *J* = 4.0 Hz), 130.58, 130.91, 134.59, 138.80, 149.54, 151.98, 167.65, 194.18. MALDI‐TOF/MS (*m*/*z*): Calculated: 507.03975; Found: 507.04324.

(*Z*)‐3‐(3,5‐bis(trifluoromethyl)benzyl)‐5‐(4‐(dimethylamino)benzylidene)‐2‐thioxothiazolidin‐4‐one (**5e**)

Recrystallized from ethyl acetate. Yield: 90%. FT‐IR (cm^−1^): 3068 (arH), 1686 (C=O), 1612 (C=N), 1169 (C=S). ^1^H NMR (400 MHz, DMSO‐*d*_6_) *δ* (ppm): 3.03 (s, 6H, 2CH_3_), 5.38 (s, 2H, CH_2_), 6.83 (d, 2H, *J* = 8.4 Hz, arH), 7.47 (d, 2H, *J* = 8.8 Hz, arH), 7.72 (s, 1H, arH), 8.00 (s, 2H, arH), 8.03 (s, 1H, CH). ^13^C NMR (APT) (100 MHz, DMSO‐*d*_6_) *δ* (ppm): 42.59, 111.19, 113.14, 119.65, 124.62, 126.99, 128.12, 137.58, 142.42, 147.63, 148.40, 154.42, 169.46. MALDI‐TOF/MS (*m*/*z*): Calculated: 490.06082; Found: 490.06310.

(*Z*)‐3‐(3,5‐bis(trifluoromethyl)benzyl)‐5‐(4‐chlorobenzylidene)‐2‐thioxothiazolidin‐4‐one (**5f**)

Recrystallized from methanol. Yield: 94%. FT‐IR (cm^−1^): 3053 (arH), 1706 (C=O), 1604 (C=N), 1165 (C=S). ^1^H NMR (400 MHz, DMSO‐*d*_6_) *δ* (ppm): 5.39 (s, 2H, CH_2_), 7.65 (d, 2H, *J* = 8.4 Hz, arH), 7.67 (d, 2H, *J* = 8.0 Hz, arH), 7.85 (s, 1H, arH), 8.03 (s, 1H, CH), 8.04 (s, 2H, arH). ^13^C NMR (APT) (100 MHz, DMSO‐*d*_6_) *δ* (ppm): 46.95, 122.05, 122.26, 123.93, 124.98, 129.37, 130.06, 130.56, 130.89, 132.30, 132.70, 136.12, 18.62, 167.63, 194.22. MALDI‐TOF/MS (*m*/*z*): Calculated: 481.97965; Found: 481.98515.

(*Z*)‐3‐(3,5‐bis(trifluoromethyl)benzyl)‐5‐(4‐bromobenzylidene)‐2‐thioxothiazolidin‐4‐one (**5g**)

Recrystallized from ethanol. Yield: 87%. FT‐IR (cm^−1^): 3053 (arH), 1704 (C=O), 1593 (C=N), 1160 (C=S). ^1^H NMR (400 MHz, DMSO‐*d*_6_) *δ* (ppm): 5.38 (s, 2H, 2CH_2_), 7.38 (t, 2H, *J* = 8.0 Hz, arH), 7.70 (s, 2H, arH), 7.86 (s, 1H, arH), 8.01 (s, 1H, CH), 8.03 (s, 2H, arH). ^13^C NMR (APT) (100 MHz, DMSO‐*d*_6_) *δ* (ppm): 46.89, 117.06, 117.28, 119.53, 122.02, 122.24, 122.76–122.79 (d, *J* = 3.0 Hz), 124.96, 127.67, 129.33, 130.07, 130.10, 130.24, 130.57, 130.90, 131.23, 133.27, 133.61–133.70 (d, *J* = 3.0 Hz), 138.63, 162.41, 164.91, 167.63, 194.27. MALDI‐TOF/MS (*m*/*z*): Calculated: 524.92914; Found: 524.91218.

(*Z*)‐3‐(3,5‐bis(trifluoromethyl)benzyl)‐5‐(4‐fluorobenzylidene)‐2‐thioxothiazolidin‐4‐one (**5h**)

Recrystallized from ethanol. Yield: 88%. FT‐IR (cm^−1^): 3053 (arH), 1707 (C=O), 1603 (C=N), 1165 (C=S). ^1^H NMR (400 MHz, DMSO‐*d*_6_) *δ* (ppm): 5.39 (s, 2H, CH_2_), 7.58 (d, 2H, *J* = 8.0 Hz, arH), 7.74 (d, 2H, *J* = 8.0 Hz, arH), 7.83 (s, 1H, arH), 8.04 (bs, 3H, arH + CH). ^13^C NMR (APT) (100 MHz, DMSO‐*d*_6_) *δ* (ppm): 46.95, 122.04, 122.26, 124.00, 124.98, 125.12, 129.34, 130.56, 130.88, 132.39, 132.61, 132.81, 132.98, 138.61, 167.64, 194.19. MALDI‐TOF/MS (*m*/*z*): Calculated: 465.00920; Found: 465.00248.

(*Z*)‐3‐(3,5‐bis(trifluoromethyl)benzyl)‐5‐(3‐nitrobenzylidene)‐2‐thioxothiazolidin‐4‐one (**5i**)

Recrystallized from methanol. Yield: 85%. FT‐IR (cm^−1^): 3066 (arH), 1701 (C=O), 1603 (C=N), 1511 (‐NO_2_), 1167 (C=S). ^1^H NMR (400 MHz, DMSO‐*d*_6_) *δ* (ppm): 5.39 (s, 2H, CH_2_), 7.88 (d, 2H, *J* = 8.0 Hz, arH), 7.93 (s, 1H, arH), 8.01 (s, 1H, CH), 8.06 (s, 2H, arH), 8.32 (d, 2H, *J* = 8.0 Hz, arH). ^13^C NMR (APT) (100 MHz, DMSO‐*d*_6_) *δ* (ppm): 47.04, 122.01, 122.26, 124.82, 124.97, 127.60, 129.40, 130.55, 130.88, 131.90, 136.46, 139.50, 148.08, 167.53, 194.07. MALDI‐TOF/MS (*m*/*z*): Calculated: 492.00370; Found: 492.03876.

(*Z*)‐3‐(3,5‐bis(trifluoromethyl)benzyl)‐5‐(3‐hydroxybenzylidene)‐2‐thioxothiazolidin‐4‐one (**5j**)

Recrystallized from ethanol. Yield: 83%. FT‐IR (cm^−1^): 3243 (‐OH), 3070 (arH), 1733 (C=O), 1643 (C=N), 1163 (C=S). ^1^H NMR (400 MHz, DMSO‐*d*_6_) *δ* (ppm): 5.39 (s, 2H, CH_2_), 7.18 (d, 1H, arH), 7.34 (s, 2H, arH), 7.43 (d, 2H, *J* = 4.0 Hz, arH), 7.97 (s, 1H, CH), 8.06 (d, 2H, *J* = 8.0 Hz, arH), 9.63 (s, 1H, OH). ^13^C NMR (APT) (100 MHz, DMSO‐*d*_6_) *δ* (ppm): 47.03, 122.32, 122.58, 124.99, 125.39, 125.57, 128.64, 129.36, 130.52, 130.88, 131.08, 131.57, 132.35, 134.91, 136.38, 138.37, 156.37, 168.94, 194.02. MALDI‐TOF/MS (*m*/*z*): Calculated: 463.01354; Found: 463.00350.

(*Z*)‐5‐(benzo[*b*]thiophen‐2‐ylmethylene)‐3‐(3,5‐bis(trifluoromethyl)benzyl)‐2‐thioxothiazolidin‐4‐one (**5k**)

Recrystallized from ethyl acetate. Yield: 86%. FT‐IR (cm^−1^): 3094 (arH), 1705 (C=O), 1593 (C=N), 1170 (C=S). ^1^H NMR (400 MHz, DMSO‐*d*_6_) *δ* (ppm): 5.41 (s, 2H, CH_2_), 7.45–7.54 (m, 2H, arH), 8.04 (s,1H, CH), 8.07 (s, 3H, arH), 8.09 (dd, 1H, *J*_*1*_ = 2.0 Hz, *J*_*2*_ = 2.0 Hz, arH), 8.18 (dd, 1H, *J*_*1*_ = 2.0 Hz, *J*_*2*_ = 2.0 Hz, arH), 8.22 (s, 1H, CH). ^13^C NMR (APT) (100 MHz, DMSO‐*d*_6_) *δ* (ppm): 46.97, 122.23, 123.53, 123.62, 124.15, 125.87, 126.17, 129.36, 129.67, 130.59, 130.91, 133.10, 138.05, 138.72, 139.56, 167.36, 194.17. MALDI‐TOF/MS (*m*/*z*): Calculated: 502.99070; Found: 502.95804.

(*Z*)‐3‐(3,5‐bis(trifluoromethyl)benzyl)‐5‐(2‐bromobenzylidene)‐2‐thioxothiazolidin‐4‐one (**5l**)

Recrystallized from ethanol. Yield: 81%. FT‐IR (cm^−1^): 3066 (arH), 1714 (C=O), 1592 (C=N), 1171 (C=S). ^1^H NMR (400 MHz, DMSO‐*d*_6_) *δ* (ppm): 5.38 (s, 2H, CH_2_), 7.39–7.43 (m, 1H, arH), 7.55 (s, 1H, arH), 7.56 (d, 1H, *J* = 2.0 Hz, arH), 7.80 (d, 1H, *J* = 8.0 Hz, arH), 7.87 (s, 1H, arH), 8.01 (s, 1H, CH), 8.06 (s, 2H, arH). ^13^C NMR (APT) (100 MHz, DMSO‐*d*_6_) *δ* (ppm): 47.04, 122.01, 122.26, 124.97, 126.23, 126.80, 129.29, 129.39, 129.98, 130.23, 130.56, 130.73, 130.89, 132.89, 132.91, 134.26, 138.53, 167.38, 194.44. MALDI‐TOF/MS (*m*/*z*): Calculated: 524.92914; Found: 525.97594.

(*Z*)‐3‐(3,5‐bis(trifluoromethyl)benzyl)‐5‐(4‐phenoxybenzylidene)‐2‐thioxothiazolidin‐4‐one (**5m**)

Recrystallized from ethanol. Yield: 82%. FT‐IR (cm^−1^): 3090 (arH), 1702 (C=O), 1583 (C=N), 1159 (C=S), 1121 (C‐O). ^1^H NMR (400 MHz, DMSO‐*d*_6_) *δ* (ppm): 5.38 (s, 2H, CH_2_), 7.10 (t, 4H, *J* = 8.0 Hz, arH), 7.23 (t, 1H, *J* = 7.2 Hz, arH), 7.44 (t, 2H, *J* = 8.0 Hz, arH), 7.65 (d, 2H, *J* = 8.0 Hz, arH), 7.83 (s, 1H, arH), 8.01 (s, 1H, CH), 8.03 (s, 2H, arH). ^13^C NMR (APT) (100 MHz, DMSO‐*d*_6_) *δ* (ppm): 46.86, 118.72, 119.53, 120.40, 121.17, 122.04, 122.24, 124.95, 125.23, 128.02, 129.35, 130.26, 130.58, 130.77, 130.91, 131.24, 133.47–133.52 (d, *J* = 5.0 Hz), 138.69, 155.35, 159.98, 167.67, 194.22. MALDI‐TOF/MS (*m*/*z*): Calculated: 539.04484; Found: 538.98622.

(*Z*)‐3‐(3,5‐bis(trifluoromethyl)benzyl)‐5‐(3,4‐dimethylbenzylidene)‐2‐thioxothiazolidin‐4‐one (**5n**)

Recrystallized from ethanol. Yield: 89%. FT‐IR (cm^−1^): 3052 (arH), 1705 (C=O), 1596 (C=N), 1163 (C=S). ^1^H NMR (400 MHz, DMSO‐*d*_6_) *δ* (ppm): 2.25 (s, 6H, 2CH_3_), 5.37 (s, 2H, CH_2_), 7.32 (d, 2H, *J* = 12.2 Hz, arH), 7.37 (s, 1H, arH), 7.74 (s, 1H, arH), 8.02 (bs, 3H, arH + CH). ^13^C NMR (APT) (100 MHz, DMSO‐*d*_6_) *δ* (ppm): 19.77, 19.96, 46.83, 122.03, 124.95, 128.77, 129.32, 130.90, 131.00, 131.09, 132.13, 134.17, 138.13, 138.70, 141.06, 167.66, 194.39. MALDI‐TOF/MS (*m*/*z*): Calculated: 475.04992; Found: 475.03521.

#### 2.1.2 Computational analysis and molecular docking of compounds

##### Computational analysis

Compounds were evaluated for their molecular and druglikeness characteristics. Using DataWarrior, we calculated their molecular weight (g/mol), cLogP (octanol/water), water solubility (cLogS), topological polar surface area (TPSA), fragment‐based druglikeness value, and toxicity risk assessment for mutagenicity, tumorigenicity, irritating effects, and reproductive effects.

##### Ligand preparation

SDF files of ligands were generated using DataWarrior (Table 1). Briefly, SMILES codes are used to generate conformers with settings as following: Random, low energy bias, Torsions based on crystallographic database, energy minimization based on MMFF94s + Forcefield. SD file version 3 with 3D atom coordinates has been used.

**TABLE 1:** SMILES of molecules used in the docking study.

Name	Molecule ID	SMILES
c‐Myc‐i1	**5a**	S=C3S\C(=C/c1ccc(cc1)[N+]([O‐])=O)C(=O)N3Cc2cc(cc(c2)C(F)(F)F)C(F)(F)F
c‐Myc‐i2	**5b**	S=C3S\C(=C/c1cc(C)cc(C)c1)C(=O)N3Cc2cc(cc(c2)C(F)(F)F)C(F)(F)F
c‐Myc‐i3	**5c**	S=C3S\C(=C/c1ccc(F)c(Cl)c1)C(=O)N3Cc2cc(cc(c2)C(F)(F)F)C(F)(F)F
c‐Myc‐i4	**5d**	S=C3S\C(=C/c1ccc(OC)c(OC)c1)C(=O)N3Cc2cc(cc(c2)C(F)(F)F)C(F)(F)F
c‐Myc‐i5	**5e**	S=C3S\C(=C/c1ccc(cc1)N(C)C)C(=O)N3Cc2cc(cc(c2)C(F)(F)F)C(F)(F)F
c‐Myc‐i6	**5f**	S=C3S\C(=C/c1ccc(Cl)cc1)C(=O)N3Cc2cc(cc(c2)C(F)(F)F)C(F)(F)F
c‐Myc‐i7	**5g**	S=C3S\C(=C/c1ccc(Br)cc1)C(=O)N3Cc2cc(cc(c2)C(F)(F)F)C(F)(F)F
c‐Myc‐i8	**5h**	S=C3S\C(=C/c1ccc(F)cc1)C(=O)N3Cc2cc(cc(c2)C(F)(F)F)C(F)(F)F
c‐Myc‐i9	**5i**	S=C3S\C(=C/c1cccc(c1)[N+]([O‐])=O)C(=O)N3Cc2cc(cc(c2)C(F)(F)F)C(F)(F)F
c‐Myc‐i10	**5j**	S=C3S\C(=C/c1cccc(O)c1)C(=O)N3Cc2cc(cc(c2)C(F)(F)F)C(F)(F)F
c‐Myc‐i11	**5k**	S=C4S\C(=C/c1cc2ccccc2s1)C(=O)N4Cc3cc(cc(c3)C(F)(F)F)C(F)(F)F
c‐Myc‐i12	**5l**	S=C3S\C(=C/c1ccccc1Br)C(=O)N3Cc2cc(cc(c2)C(F)(F)F)C(F)(F)F
c‐Myc‐i13	**5m**	S=C4S\C(=C/c2ccc(Oc1ccccc1)cc2)C(=O)N4Cc3cc(cc(c3)C(F)(F)F)C(F)(F)F
c‐Myc‐i14	**5n**	S=C3S\C(=C/c1ccc(C)c(C)c1)C(=O)N3Cc2cc(cc(c2)C(F)(F)F)C(F)(F)F

##### Docking parameters and protein preparation

The Myc–Max structure in complex with DNA (PDB ID 1NKP) has been used for docking studies. Briefly, crystal structure of protein was downloaded in pdb format from https://www.rcsb.org. Receptor structure was checked for incorrect charges and missing atoms and was optimized by removing water molecules, DNA, and ions in the PDB ID: 1NKP. A grid box with 30^o^A × 30^o^A × 30^o^A and coordinates of *x* = 68.457, *y* = 84.469, *z* = 22.076 and spacing of 1^o^A around the DNA binding pocket for PDB ID 1NKP has been generated using AutoDockTools 1.5.6. Automated docking of ligands was performed with PaDelADV.

##### Structural clustering

ChemBioServer 2.0 was used for clustering of compounds (https://chembioserver.vi‐seem.eu/index.php) based on the distance form of Soergel (Tanimoto Coefficient) and average linkage.

#### 2.1.3 Cell culture

We utilized a panel of cancer cell lines, including A549 (lung cancer), Panc1 (pancreatic cancer), DU‐145 (prostate cancer), and MCF7 (breast cancer), in addition to the healthy cell line, human dermal fibroblasts (HDF). These cells were cultured in high‐glucose Dulbecco's modified Eagle's medium (DMEM) supplemented with 10% fetal bovine serum (FBS) and 1% penicillin/streptomycin mix (Thermo Fisher Scientific, cat. no.: 5240062). Incubation was carried out at 37°C under an atmosphere comprising 5% CO_2_.

#### 2.1.4 Cell viability and IC50 analysis

Cancer or healthy HDF cells were seeded at suitable densities (around 70%) in triplicate wells and incubated for 24 h at 37°C 5% CO_2_ before the addition of compounds or DMSO as a vehicle control. Compounds were diluted in DMSO and initially tested at 25 μM doses. MTS (10% v/v) (Promega, cat. no.: G3582) was applied to test cell viability after 48 h of treatments. Absorbance was measured 2 h after MTS addition using a Varioskan LUX Multimode Microplate Reader (Thermo Fisher Scientific) at 490 nm. To build dose–response curves, raw absorbance values were employed. Four or three parameter logistic regression model analysis was used. IC50 Calculation Tool/AATBioquest was used to obtain half maximal inhibitory concentration (IC50) values for compounds.

#### 2.1.5 Apoptosis analysis

For the apoptosis assay, we employed the ‘eBioscience Annexin V Apoptosis Detection Kit’. In six‐well plates, DU‐145 cells in logarithmic growth phase were seeded at a density of 400,000 cells/well, and MCF7 cells at a density of 500,000 cells/well, to investigate the impact of chemicals on cell apoptosis. Following a 24‐h incubation period at 37°C and 5% CO_2_, cells were treated with either DMSO (0.1%) or effective dosages (2 μM) of small molecules. After 72 h of incubation, cells were harvested from the six‐well plates using trypsin and subsequently centrifuged at 1500 rpm for 5 min. The resulting pellet was re‐suspended in 400 μL of 1X binding buffer. For apoptosis assessment, 2 μL of Annexin V‐FITC was added to the remaining cell suspension, followed by mixing and a 10‐min incubation at room temperature. Apoptosis detection was conducted with analysis of 10,000 cells per event performed on the FITC‐A and PC5.5 channels. For apoptosis assessment, the cells were re‐suspended in 190 μL of 1X binding buffer, followed by the addition of propidium iodide (PI). Flow cytometry analysis was performed using a Cytoflex S flow cytometer (Beckman).

#### 2.1.6 Cell cycle analysis

For cell cycle analysis, 5 μL of Hoechst 33342 was added to the isolated cells and incubated at 37°C for 45 min. Following this incubation, 3 μL of Pyronin Y was added directly, and the mixture was incubated at room temperature for an additional 15 min. After completing the incubation, the cells were subjected to flow cytometry analysis (Cytoflex S), utilizing the DAPI and PE channels.

#### 2.1.7 Statistical analysis

For statistical analysis, Student's *t*‐test was used. *p* < 0.05 is regarded as statistically significant.

## 3 RESULTS

### 3.1 Design strategy

**SCHEME 1:**
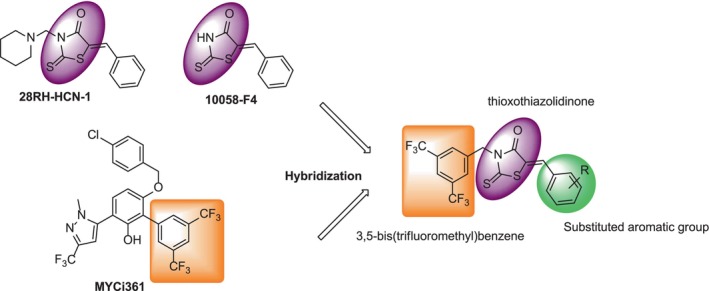
Design of novel rhodanine derivatives.

The structure of target rhodanine derivatives was designed by the molecular hybridization approach based on the structure of some small molecule inhibitors of c‐Myc as reported in the literature (compounds A–C; Scheme 1). The compound 10058‐F4 was found to be c‐Myc inhibitor against HL60 cells with an IC50 value of 49.0 μM, while the compound 28RH‐NCN‐1 demonstrated more potential in inhibiting the binding of c‐Myc/Max to E‐box motifs on the same cells.^19^, ^20^ Further, the compound MYCi361 inhibits MYC‐dependent cancer cell proliferation and tumorigenicity, for example, it showed potency against MycCaP cell with an IC50 value of 2.9 μM.^13^ Considering these structural features, we designed a series of c‐Myc inhibitors by integrating both 3,5‐bis(trifluoromethyl)benzene and thioxothiazolidinone ring.

### 3.2 Synthesis

**SCHEME 2:**
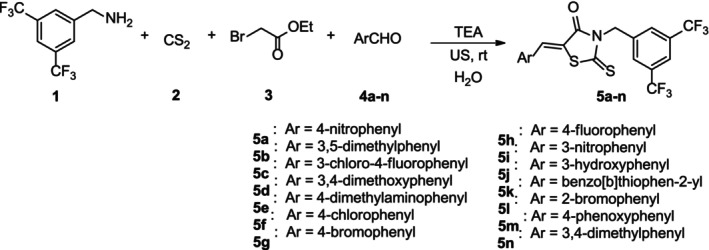
One‐pot four‐component synthesis of rhodanine derivatives.

A series of arylidene thioxothiazolidin‐4‐one derivatives was synthesized as a one‐pot four‐component reaction according to the general synthetic pathway described in Scheme 2. Initially, commercially available 3,5‐bis(trifluoromethyl)benzylamine (1) was reacted with carbon disulphide (2) and ethyl bromoacetate (3) in the presence of H_2_O under sonication for 5 min to give the intermediate.^21^ Then, triethylamine and corresponding aromatic aldehyde (4) was added to the reaction mixture and sonicated for 5 min in order to carry out the final step of intramolecular ring closure. Considering the optimization studies examining the effect of the base on the reaction and our groups' previous studies,^22^, ^23^ it was achieved high reaction yields by using TEA in the range of 81%–94%. The structures of purified compounds were verified by FTIR, MALDI‐TOF/MS, ^1^H‐NMR, and ^13^C‐NMR spectroscopy (Supplementary File).

### 3.3 Effect of novel c‐Myc/Max inhibitors in pathologically distinct cancer cell viability

We evaluated the efficacy of compounds across distinct cancer types using the PANC1 pancreatic adenocarcinoma cell line, A549 lung cancer line, DU‐145 prostate cancer line, and MCF7 breast cancer line. These cancer cells were seeded into 96‐well plates and exposed to 25 μM concentrations of various small molecules, spanning from c‐Myc‐i1 to c‐Myc‐i14. DMSO and 10058‐F4 were included as control treatments. After 3 days of exposure, we assessed the viability of the cancer cells.

**FIGURE 1:**
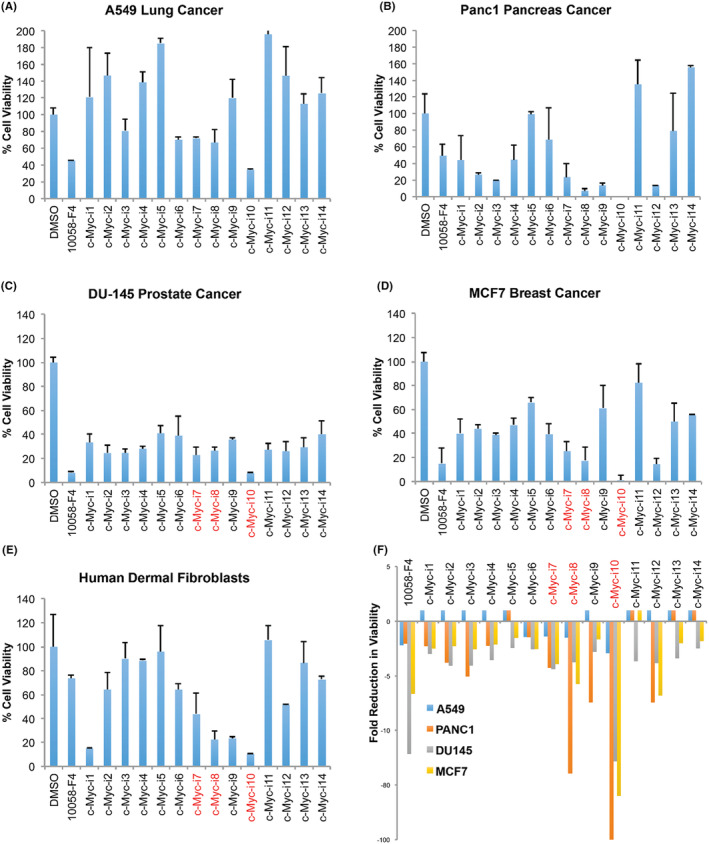
Analysis of novel c‐Myc derivatives in pathologically distinct cancers. Cell viability post treatment with 25 μM of novel c‐Myc inhibitors (c‐Myc‐i) in (A) A549 lung cancer, (B) Panc1 pancreatic cancer, (C) Du‐145 prostate cancer, (D) MCF7 breast cancer, and (E) human dermal fibroblasts. (F) Fold reduction in cancer cell viability post c‐Myc‐i treatments compared to DMSO control. *n* = 3.

Investigating the effect of c‐Myc inhibitors at a concentration of 25 μM on A549 lung cancer cells revealed that four compounds c‐Myc‐i6, c‐Myc‐i7, c‐Myc‐i8 and c‐Myc‐i10 significantly reduced cell viability by at least 30%, similar to the reference compound 10058‐F4 (Figure 1A). In the case of PANC1 pancreatic adenocarcinoma cells treated with c‐Myc inhibitors at a 25 μM final dose, four compounds c‐Myc‐i7, c‐Myc‐i8, c‐Myc‐i10 and c‐Myc‐i12 displayed substantial reductions in cell viability, exceeding 50% and outperforming the reference compound 10058‐F4 (Figure 1B). Furthermore, DU‐145 prostate cancer cells treated with c‐Myc inhibitors at a concentration of 25 μM exhibited a significant decrease in cell viability, with all compounds causing reductions of at least 60%. Notably, c‐Myc‐i7, c‐Myc‐i8 and c‐Myc‐i10 achieved reductions ranging from 80% to 90% (Figure 1C). MCF7 breast cancer cells treated with c‐Myc inhibitors at 25 μM demonstrated a substantial reduction in viability, with reductions of up to 95%. Specifically, c‐Myc‐i7, c‐Myc‐i8 and c‐Myc‐i12 exhibited reductions comparable to or better than those achieved by 10058‐F4, the positive control (Figure 1D). We also evaluated the cytotoxicity of c‐Myc inhibitors on HDF. While c‐Myc‐i3, c‐Myc‐i4, and c‐Myc‐i5 did not display cytotoxic effects, both 10058‐F4 (resulting in at least a 20% reduction in viability) and several other c‐Myc inhibitors, including c‐Myc‐i7, c‐Myc‐i8 and c‐Myc‐i10, exhibited cytotoxicity (Figure 1E). Overall, our findings highlight the anti‐cancer properties of c‐Myc‐i7, c‐Myc‐i7 and c‐Myc‐i10 across four different cancer types, as evidenced by their remarkable reductions in cell viability (Figure 1G).

### 3.4 Determination of IC50 values of selected c‐Myc inhibitors in healthy and cancer cell lines

**FIGURE 2:**
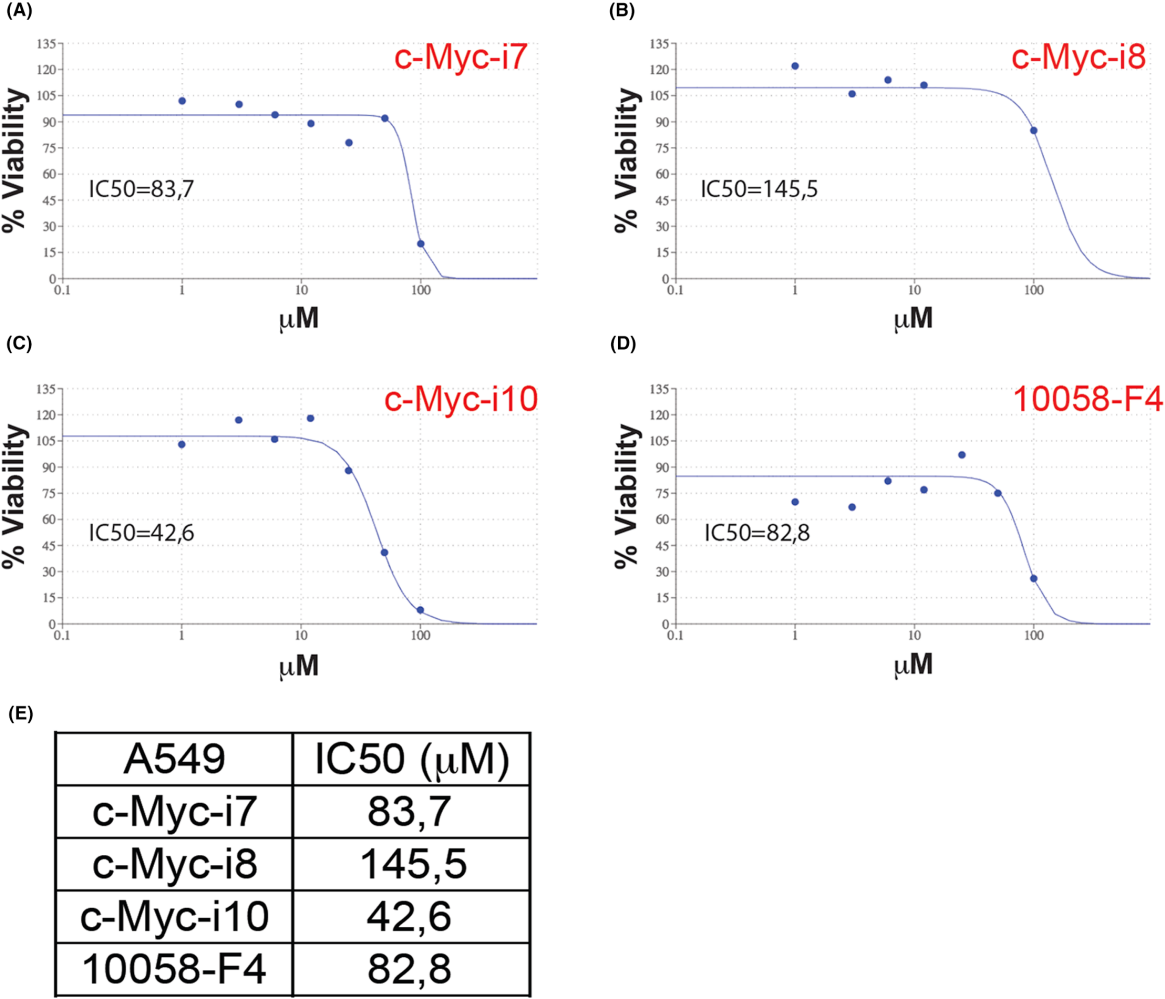
Determination of IC50 values in A549 cells post c‐Myc‐i treatments. IC50 curve of (A) c‐Myc‐i7, (B) c‐Myc‐i8, (C) c‐Myc‐i10, and (D) 10058‐F4. (E) Quantification of IC50 values.

To determine the IC50 values of c‐Myc inhibitors in both cancer and healthy cells, we plated the cells in triplicate wells at appropriate densities and incubated them for 24 h. Subsequently, compounds were diluted in DMSO across a range of concentrations from 0.01 to 100 μM. After 48 h of treatment, cell viability was assessed using MTS assays. The raw absorbance data were used to generate dose–response curves, and analysis was performed using a four or three‐parameter logistic regression model. Testing of c‐Myc‐i7, c‐Myc‐i8 and c‐Myc‐i10, in addition to the positive control 10058‐F4, in A549 lung cancer cells revealed IC50 values spanning from 42 to 145 μM (Figure 2A–E). Particularly noteworthy, c‐Myc‐i10 exhibited approximately twice the efficacy in lung cancer (IC50 of 42.6 μM) compared to 10058‐F4 (IC50 of 82.8 μM). While c‐Myc‐i7 (IC50 of 83.7 μM) demonstrated efficacy similar to that of 10058‐F4, c‐Myc‐i8 displayed reduced efficacy in lung cancer (IC50 of 145.5 μM).

**FIGURE 3:**
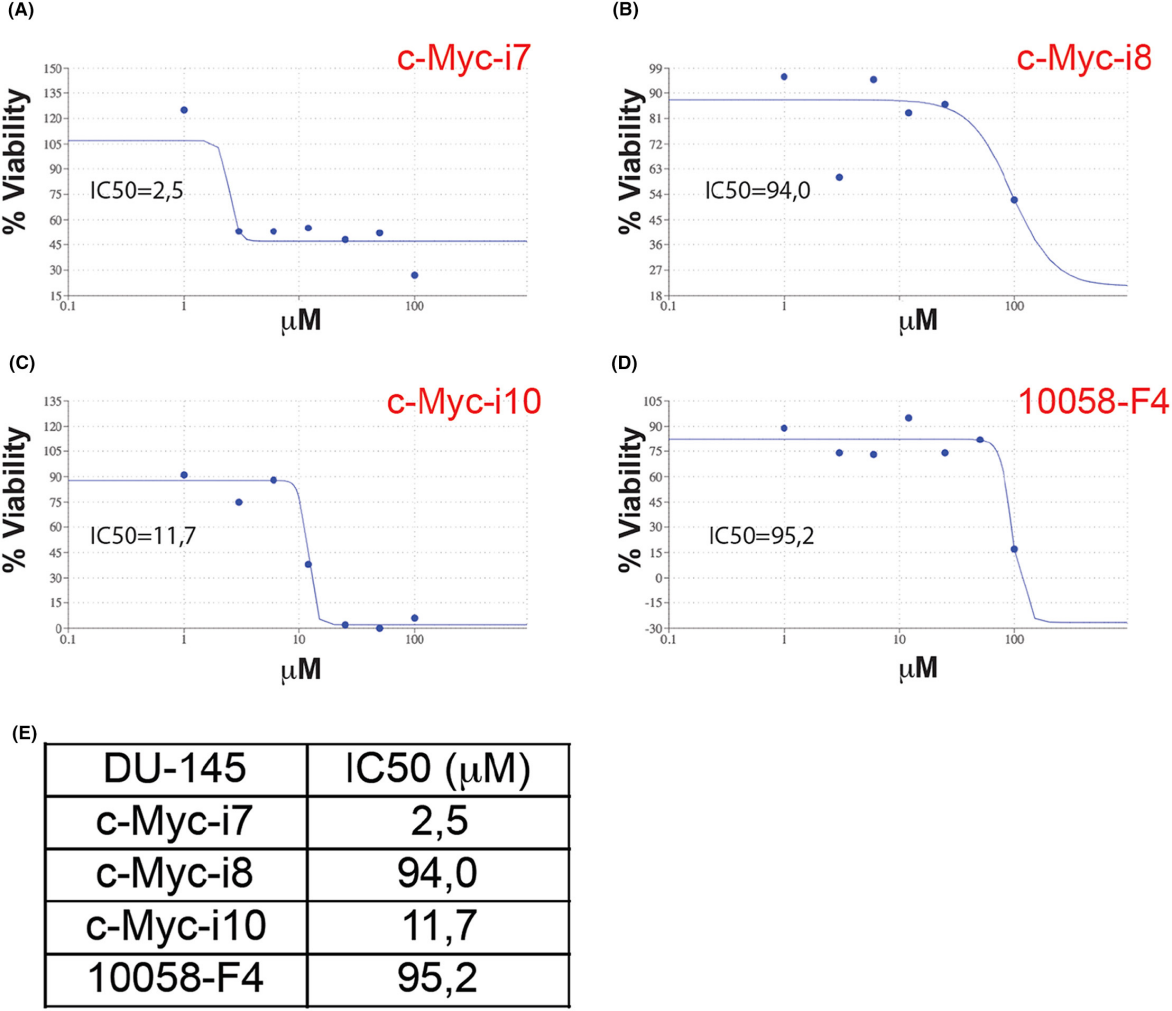
Determination of IC50 values in DU‐145 cells post c‐Myc‐i treatments. IC50 curve of (A) c‐Myc‐i7, (B) c‐Myc‐i8, (C) c‐Myc‐i10 and (D) 10058‐F4. (E) Quantification of IC50 values.

Our investigation, conducted on DU‐145 prostate cancer cells, aimed to determine the IC50 values for c‐Myc‐i7, c‐Myc‐i8, and c‐Myc‐i10, in addition to the positive control 10058‐F4. The results exhibited a wide range, spanning from 2.5 to 95 μM (Figure 3A–E). Notably, c‐Myc‐i7 demonstrated an exceptional approximately 40‐fold increase in effectiveness against prostate cancer (IC50 of 2.5 μM) when compared to 10058‐F4 (IC50 of 95.2 μM). While both c‐Myc‐i8 (IC50 of 94.0 μM) and c‐Myc‐i10 (11.7 μM) exhibited considerable efficacy, the latter showed an eight‐fold improvement in its performance against prostate cancer compared to 10058‐F4.

**FIGURE 4:**
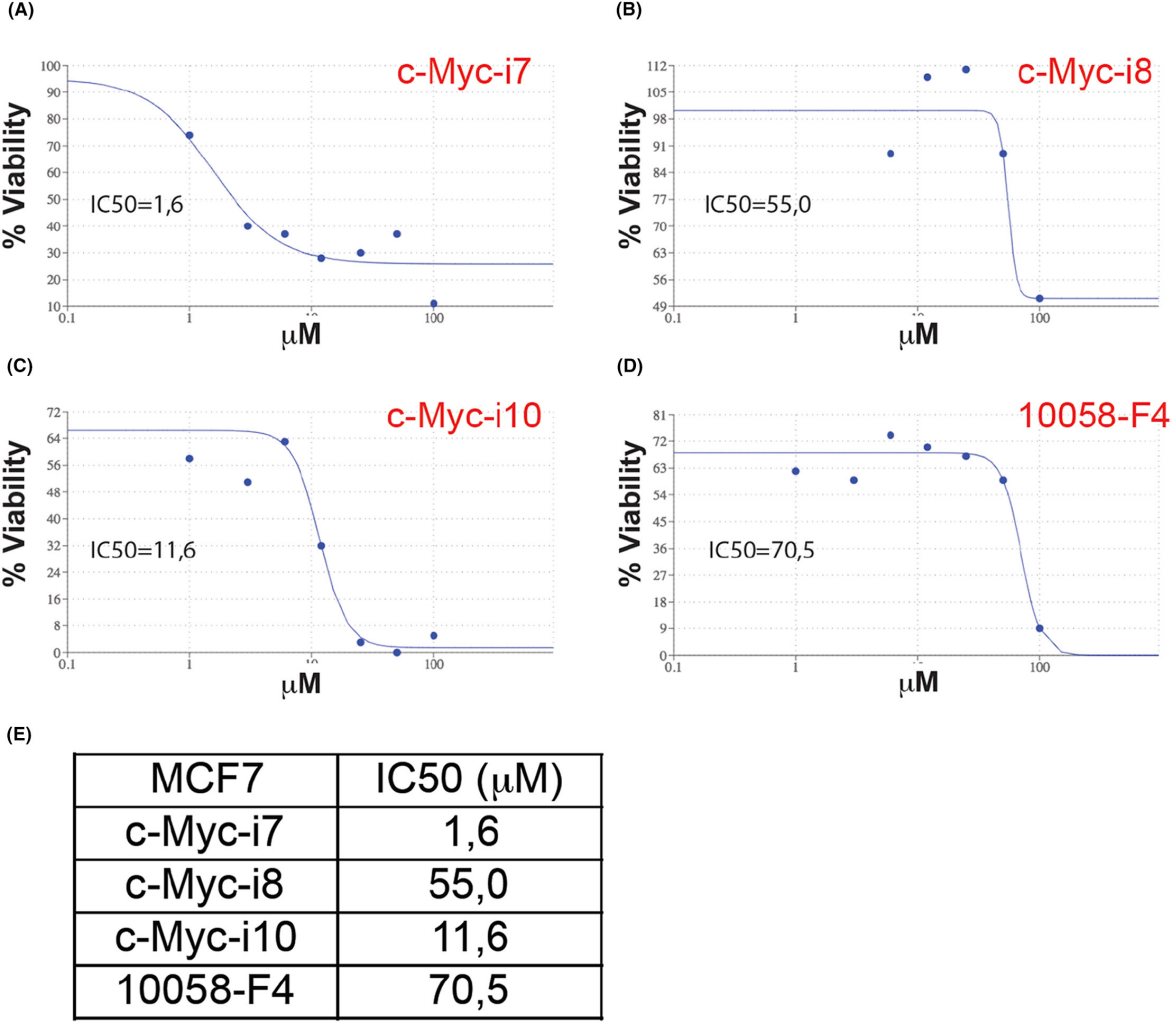
Determination of IC50 values in MCF7 cells post c‐Myc‐i treatments. IC50 curve of (A) c‐Myc‐i7, (B) c‐Myc‐i8, (C) c‐Myc‐i10, and (D) 10058‐F4. (E) Quantification of IC50 values.

Experiments on MCF7 breast cancer cells determined the IC50 values for c‐Myc‐i7, c‐Myc‐i8 and c‐Myc‐i10, alongside 10058‐F4, ranging from 1.6 to 70.5 μM (Figure 4A–E). Notably, c‐Myc‐i7 and c‐Myc‐i10 showed a 44‐ and six‐fold increase in efficacy (IC50 of 1.6 and 11.6 μM, respectively) compared to 10058‐F4 (IC50 of 70.5 μM). c‐Myc‐i8 (55.0 μM) also displayed improved efficacy against breast cancer relative to 10058‐F4.

**FIGURE 5:**
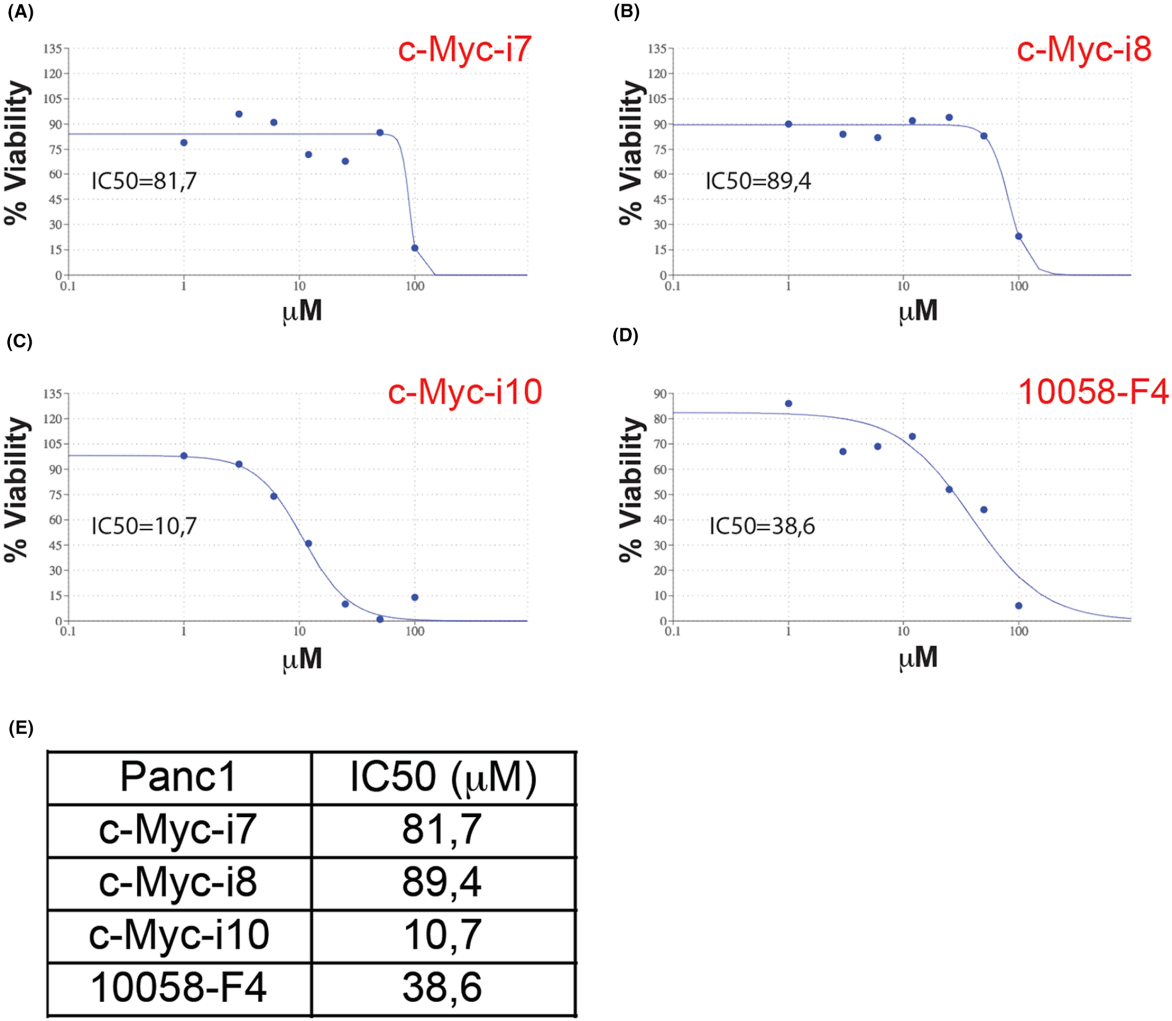
Determination of IC50 values in Panc1 cells post c‐Myc‐i treatments. IC50 curve of (A) c‐Myc‐i7, (B) c‐Myc‐i8, (C) c‐Myc‐i10, and (D) 10058‐F4. (E) Quantification of IC50 values.

Experiments conducted on Panc1 pancreatic cancer cells assessed the IC50 values for c‐Myc‐i7, c‐Myc‐i8 and c‐Myc‐i10, alongside the positive control 10058‐F4, with values spanning from 10 to 89 μM (Figure 5A–E). Particularly noteworthy was the performance of c‐Myc‐i10, which exhibited approximately a four‐fold increase in efficacy against pancreatic cancer (IC50 of 10.7 μM) compared to 10058‐F4 (IC50 of 38.6 μM). Conversely, both c‐Myc‐i7 (IC50 of 81.7 μM) and c‐Myc‐i8 (89.4 μM) displayed reduced effectiveness in treating pancreatic cancer when compared to 10058‐F4.

We further explored the selectivity of novel c‐Myc inhibitors in targeting cancer cells compared to healthy ones. We employed HDF as a representative of healthy cells to assess selectivity across various cancer types. Selectivity was determined by calculating the ratio of IC50 values for HDFs to cancer cells (Table 2). c‐Myc‐i10 displayed high cytotoxicity to normal cells with an IC50 of 11.6 μM. The selectivity ratios for 10058‐F4 and all other compounds tested typically ranged from 0.3 to 2.4 in pathologically distinct cancers. Notably, c‐Myc‐i7 exhibited an impressive selectivity of up to 59 for breast cancer. Additionally, c‐Myc‐i7 exhibited notable selectivity towards DU‐145 prostate cancer cells, displaying a selectivity ratio of 37.4.

**TABLE 2:** Selectivity analysis against healthy cells. We performed in vitro cytotoxicity to healthy cells (HDFs) to determine selectivity of compounds to cancer cells in comparison. Selectivity to cancer cells is determined by calculating the ratio of IC50 values for HDF/cancer cells. Note that c‐Myc‐i7 demonstrated up to 59‐fold selectivity to cancer cells.

Healthy cells (HDF)		Selectivity to cancer cell lines (IC50 ratios of HDF cancer cells)			
Compound	IC50 value	A549	DU‐145	MCF7	Panc1
c‐Myc‐i7	91.5 μM	1.1	37.4	59.7	1.1
c‐Myc‐i8	66.4 μM	0.5	0.7	1.2	0.7
c‐Myc‐i10	11.6 μM	0.3	1.0	1.0	1.1
10058‐F4	91.5 μM	1.1	1.0	1.3	2.4

In conclusion, this study highlights c‐Myc‐i7 as a promising candidate among c‐Myc inhibitors. It demonstrated significant efficacy across various cancer types, including lung, prostate, breast, and pancreatic cancers. Notably, c‐Myc‐i7 showed exceptional selectivity for breast cancer and DU‐145 prostate cancer cells. These findings emphasize the potential of c‐Myc‐i7 as a valuable therapeutic agent in treating these cancers, contributing to the advancement of cancer therapy.

### 3.5 The impact of c‐Myc‐i7 on apoptosis and cell cycle regulation in breast and prostate cancer cells

**FIGURE 6:**
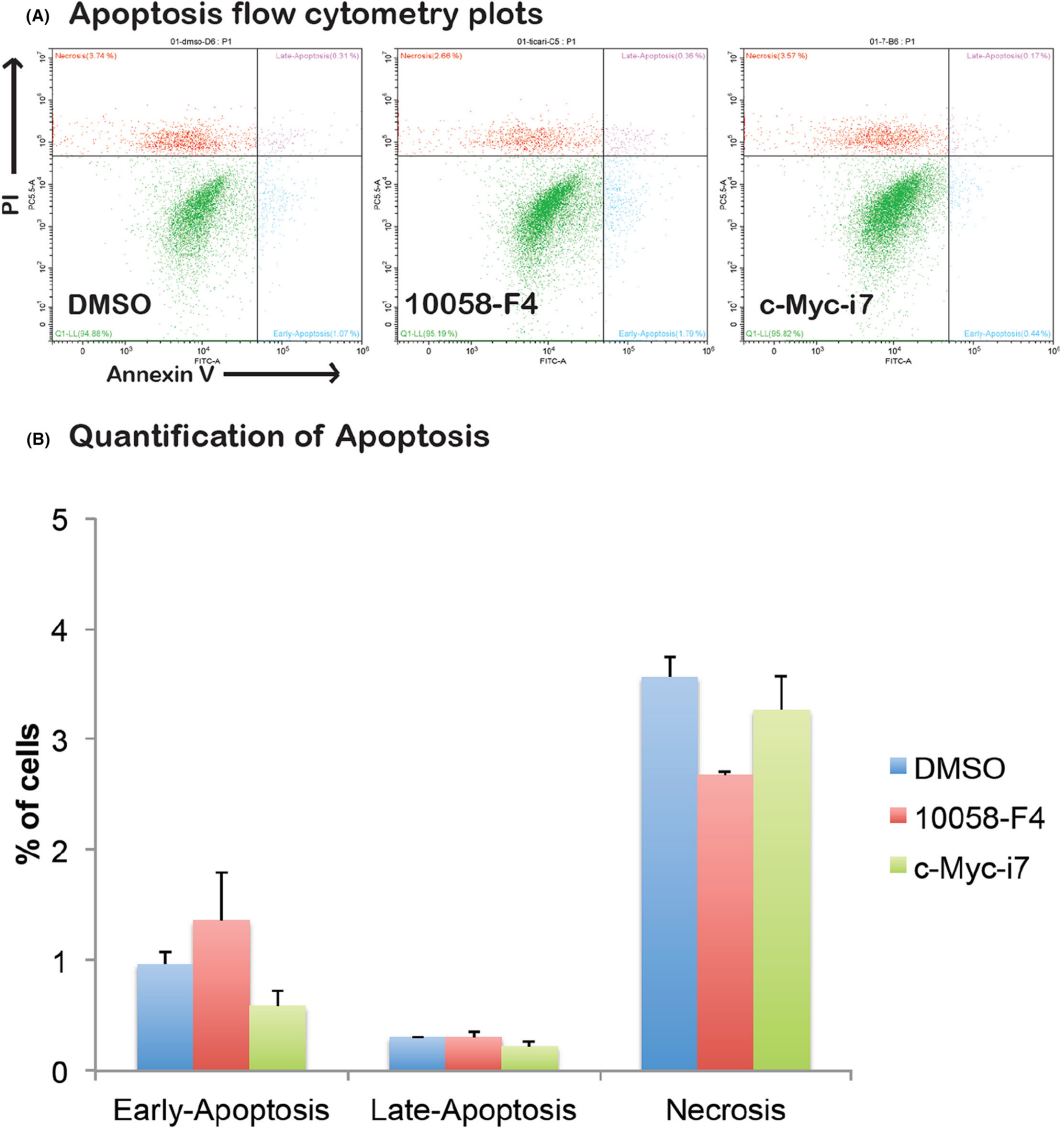
Apoptosis analysis of MCF7 Cells. MCF7 cells are treated with 2 μM of 10058‐F4 and c‐Myc‐i7 and tested for their apoptotic effect. (A) Apoptosis flow cytometry plots. (B) Quantification of early apoptotic, late apoptotic and necrotic cells.

**FIGURE 7:**
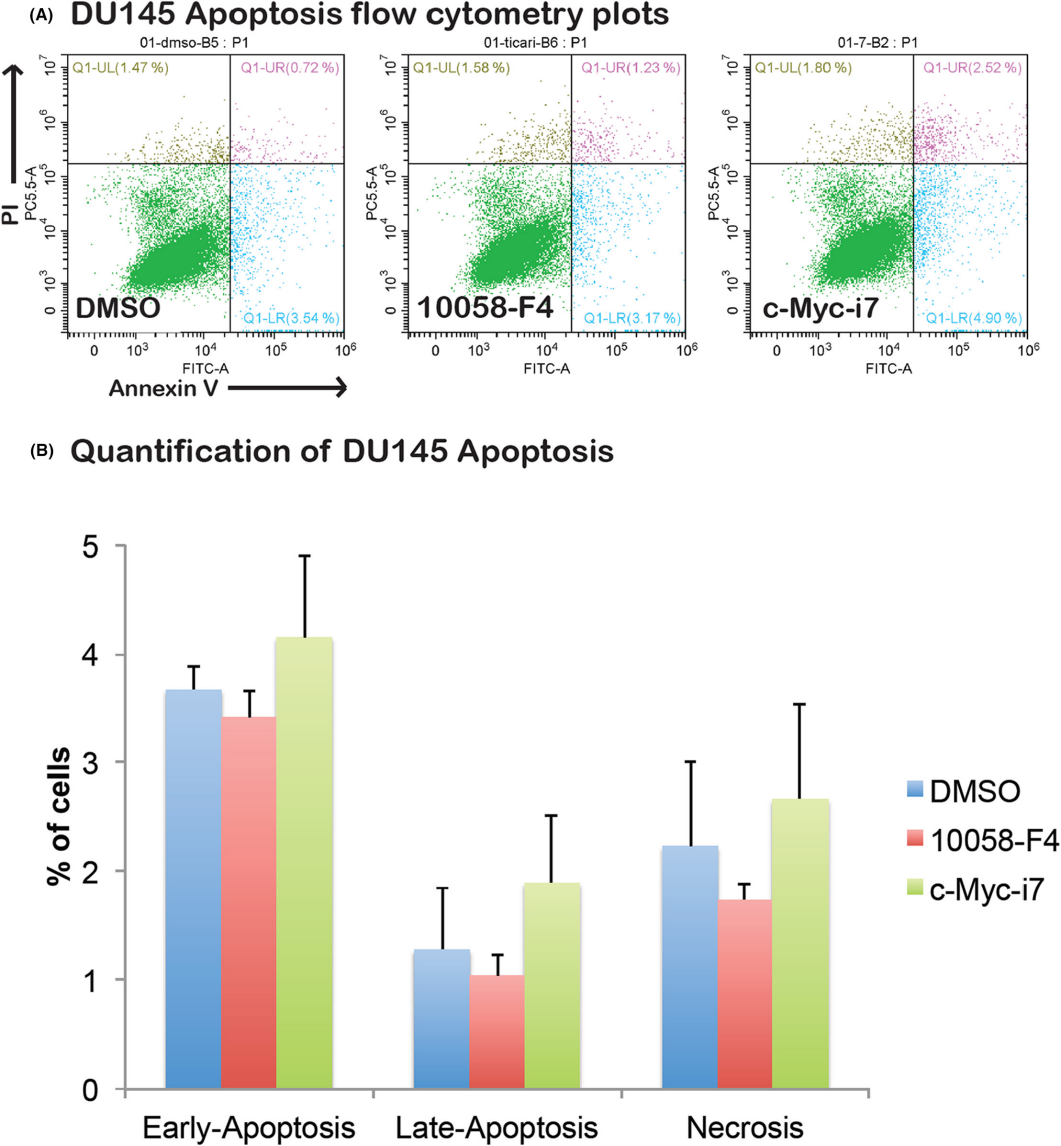
Apoptosis analysis of DU145 Cells. DU145 cells are treated with 2 μM of 10058‐F4 and c‐Myc‐i7 and tested for their apoptotic effect. (A) Apoptosis flow cytometry plots. (B) Quantification of early apoptotic, late apoptotic and necrotic cells.

**FIGURE 8:**
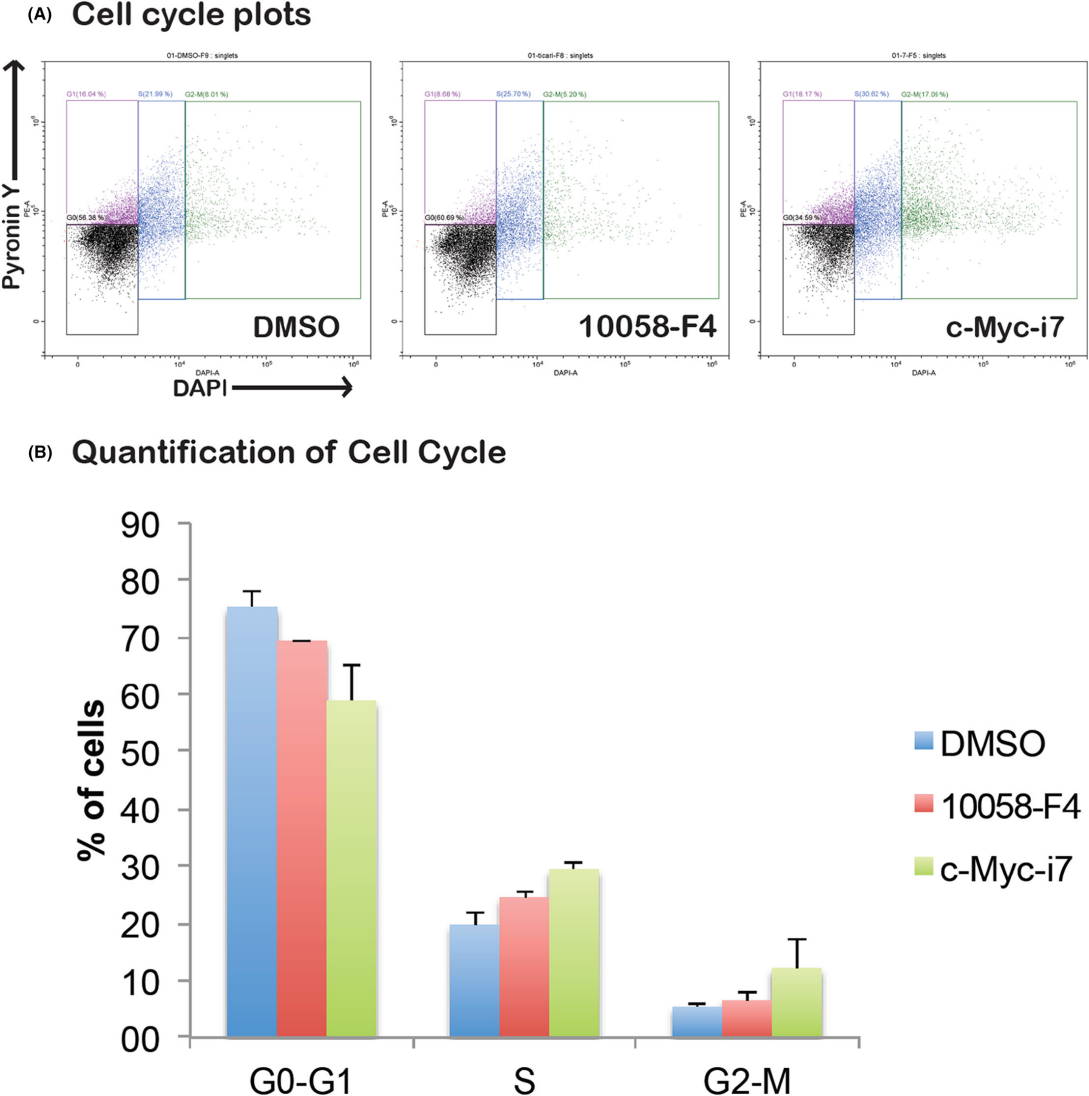
Cell cycle analysis of MCF7 cells. MCF7 cells are treated with 10058‐F4 and c‐Myc‐i7 and tested for their effect in the cell cycle. (A) Cell cycle flow cytometry plots. (B) Quantification of cells in G0‐G1, S and G2‐M phases of cell cycle.

**FIGURE 9:**
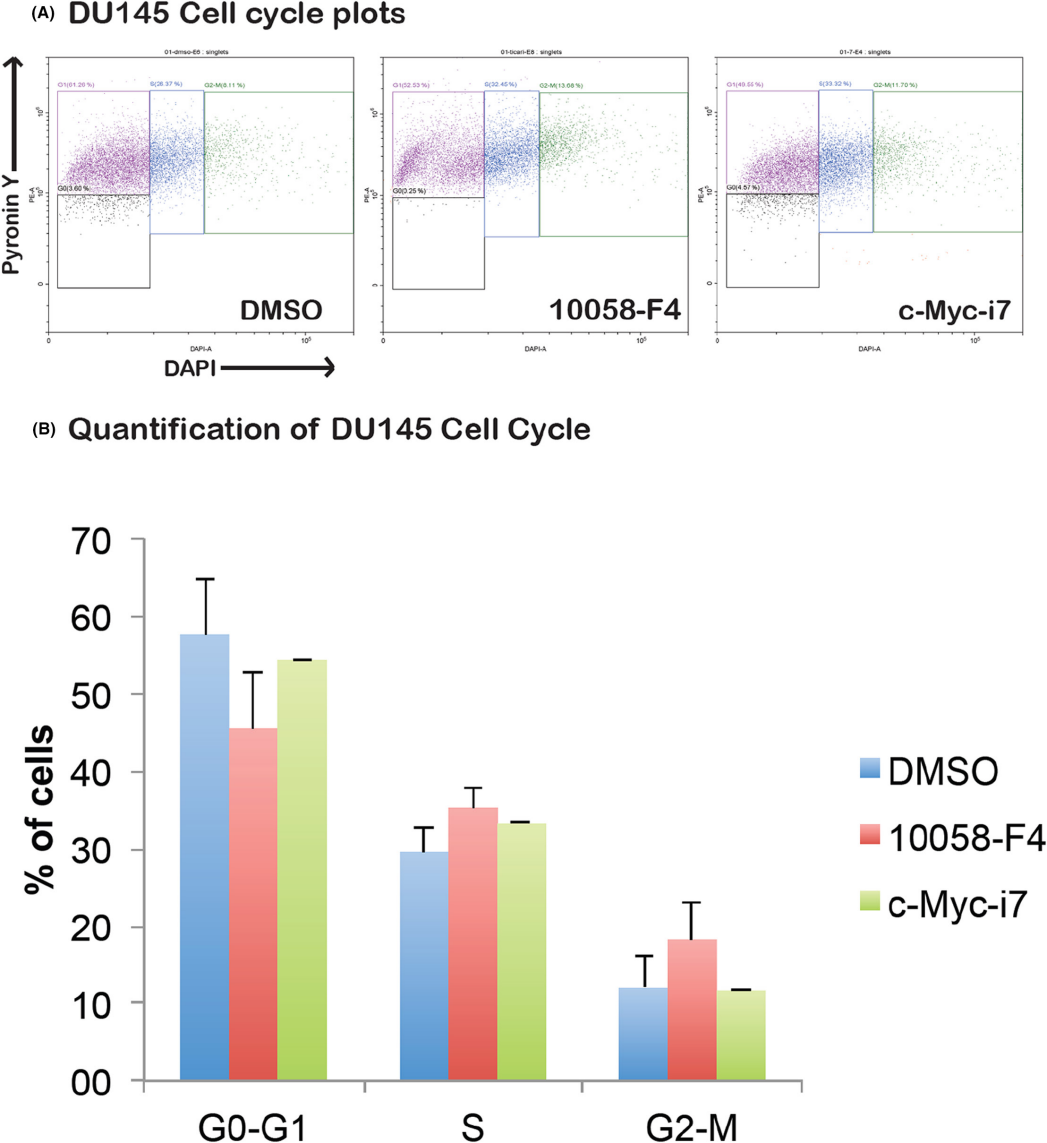
Cell cycle analysis of DU145 Cells. DU145 cells are treated with 10058‐F4 and c‐Myc‐i7 and tested for their effect in the cell cycle. (A) Cell cycle flow cytometry plots. (B) Quantification of cells in G0‐G1, S and G2‐M phases of cell cycle.

Examining the impact of c‐Myc‐i7 on apoptosis and cell cycle regulation in breast and prostate cancer cells, we observed distinct outcomes. In MCF7 cells, c‐Myc‐i7 did not induce apoptosis (Figure 6A,B). However, in DU‐145 cells, c‐Myc‐i7 treatment led to a notable increase in apoptosis compared to both DMSO and 10058‐F4 treatments (Figure 7A,B). We also assessed the influence of c‐Myc‐i7 on cell cycle profiles in these cancer cell lines. In MCF7 breast cancer cells, c‐Myc‐i7 treatment resulted in an increase in the S1 and G2‐M cell populations (Figure 8A,B). Conversely, DU145 cells exhibited a slight rise in the percentage of cells in the S phase of the cell cycle when treated with c‐Myc‐i7, similar to the effect observed with 10058‐F4 (Figure 9A,B). In summary, our findings reveal that c‐Myc‐i7 treatment elicits distinct alterations in cell cycle and apoptosis dynamics in breast and prostate cancer cell lines, underscoring its differential impact on these malignancies.

### 3.6 Molecular docking and druglikeness of compounds

A computational analysis was conducted to evaluate the molecular and druglikeness characteristics of the compounds of interest. Key parameters, including molecular weight (g/mol), cLogP (octanol/water partition coefficient), water solubility (cLogS), topological polar surface area (TPSA), fragment‐based druglikeness value, and toxicity risk assessments (covering mutagenicity, tumorigenicity, irritating effects and reproductive effects), were meticulously calculated using DataWarrior (Table 3).

**TABLE 3:** Computational analysis of druglikeness of compounds.

Compound number	Molecular weight	cLogP	cLogS	Polar Surface	Druglikeness	Mutagenicity prediction	Tumorigenicity prediction	Reproductive effect	Irritant prediction
c‐Myc‐i1	492.419	35.902	−6.477	123.52	−82.913	None	None	None	None
c‐Myc‐i2	475.476	51.996	−6.705	77.7	−32.773	None	None	None	None
c‐Myc‐i3	499.857	52.186	−7.067	77.7	−3.185	None	None	None	None
c‐Myc‐i4	507.474	43.718	−6.053	96.16	−32.024	None	None	None	None
c‐Myc‐i5	490.491	44.082	−6.053	80.94	−2.475	High	High	None	None
c‐Myc‐i6	481.867	51.178	−6.753	77.7	−3.185	None	None	None	None
c‐Myc‐i7	526.318	5.237	−6.851	77.7	−5.027	None	None	Low	None
c‐Myc‐i8	465.412	46.126	−6.331	77.7	−3.237	None	None	None	None
c‐Myc‐i9	492.419	35.902	−6.477	123.52	−82.913	None	None	None	None
c‐Myc‐i10	463.421	41.661	−5.721	97.93	−32.268	None	None	None	None
c‐Myc‐i11	503.510	5.433	−7.366	105.94	−3.237	None	None	None	None
c‐Myc‐i12	526.318	5.237	−6.851	77.7	−5.027	None	None	None	None
c‐Myc‐i13	539.519	59.074	−8.314	86.93	−32.638	None	None	None	None
c‐Myc‐i14	475.476	51.996	−6.705	77.7	−32.773	None	None	None	None

**FIGURE 10:**
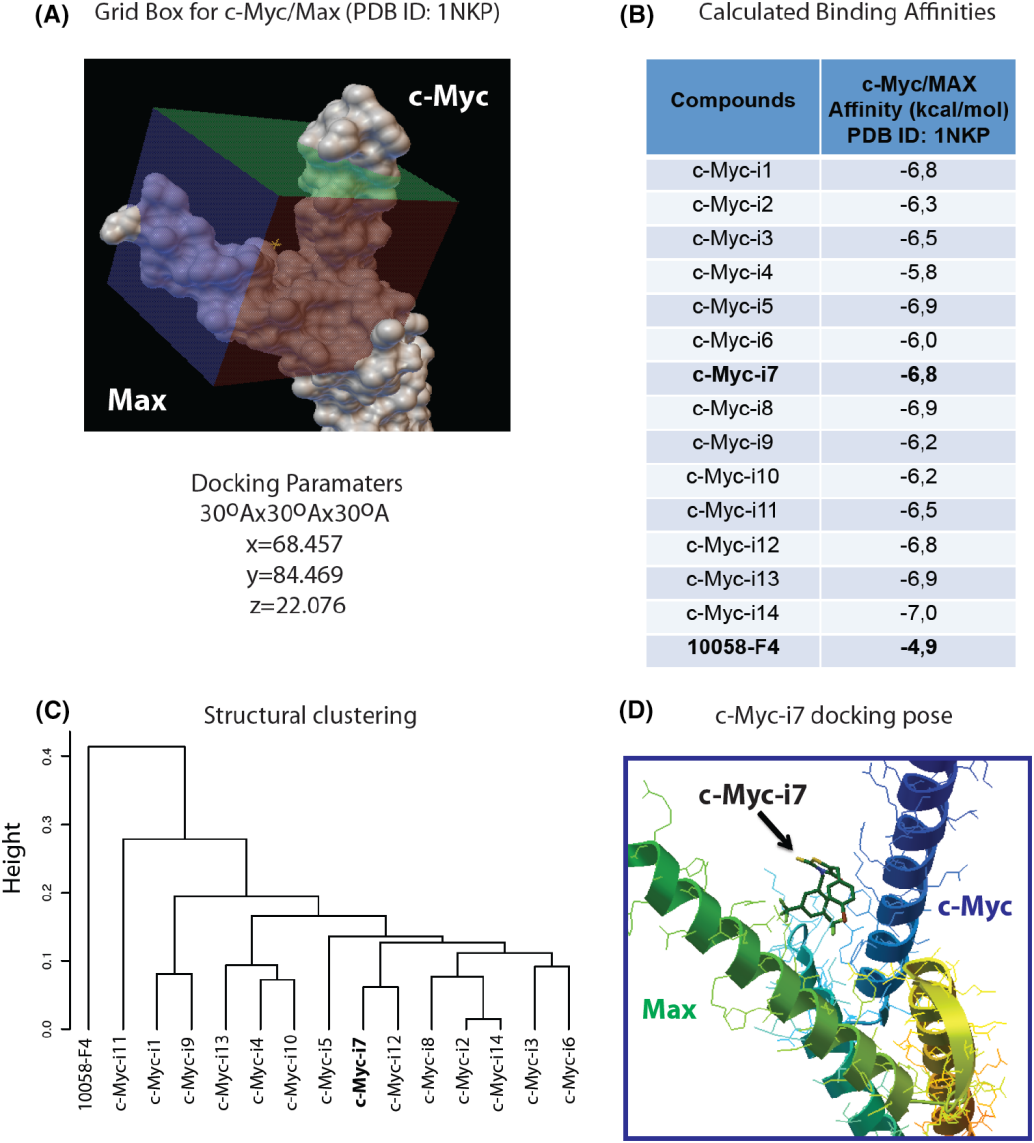
Molecular docking and structural clustering of compounds. (A) Grid box and docking parameters used in the study. (B) Calculated binding affinities for compounds in the c‐Myc/Max DNA binding pocket (DBD). (C) Structural clustering of compounds based on the Soergel distance. (D) Docking pose of c‐Myc‐i7 in the c‐Myc/Max DBD pocket.

To facilitate subsequent affinity calculations and docking analyses, SDF files for ligands were generated through DataWarrior, employing SMILES codes to create conformers with specific settings for randomization, low energy bias, and torsions based on a crystallographic database. For the docking studies, we utilized the crystal structure of the Myc–Max complex with DNA (PDB ID 1NKP) as the receptor. A grid box was generated around the DNA binding pocket of PDB ID 1NKP with specific dimensions (30 Å × 30 Å × 30 Å) and coordinates (Figure 10A). Automated docking of ligands and affinity calculations was performed using PaDelADV and AutodockVina (Figure 10B).

Highest affinity of tested compounds to Myc–Max complex was −7.0 kcal/mol. Notably, 10058‐F4 displayed an affinity of −4.9 kcal/mol, while c‐Myc‐i7 demonstrated a substantially higher affinity of −6.8 kcal/mol towards the Myc–Max complex. This highlights the promising binding capabilities of c‐Myc‐i7 in comparison to the reference compound. Additionally, structural clustering of the compounds was carried out using the Soergel distance form (tanimoto coefficient) and employing an average linkage method. This clustering approach provided valuable insights into the structural relationships among the compounds, enhancing our understanding of their properties and potential interactions (Figure 10C). We also confirmed c‐Myc‐i7 interacts with the Myc–Max complex (Figure 10D).

The identification of highly bioactive compounds often coincides with an increase in their molecular weights, presenting a challenge for drug developers. Larger molecules tend to have reduced absorption and efficacy, making it imperative for pharmaceutical developers to prioritize minimizing molecular weights in drug design. This attribute served as a crucial indicator of a potential medicine in this study. In our study, we have successfully synthesized a range of small molecules with the potential to act as c‐Myc inhibitors, each demonstrating varying levels of potency. Further investigation revealed that some of these compounds possess unique structural features, rendering them promising candidates for lead compounds. These findings underscore the significance of conducting additional in vitro analyses on c‐Myc inhibitors across diverse cancer types, drug‐resistant models, or other phenotypic assays, potentially unveiling novel, and potent therapeutic compounds.

## 4 DISCUSSION

In the present study, we employed a molecular hybridization approach, drawing inspiration from known c‐Myc inhibitors reported in the literature (compounds A‐C), such as 10058‐F4, 28RH‐NCN‐1, and MYCi361. These compounds have demonstrated varying levels of c‐Myc inhibition against different cancer cell lines.^1^, ^2^, ^3^ With these structural insights in mind, we designed a series of novel c‐Myc inhibitors by incorporating 3,5‐bis(trifluoromethyl)benzene and thioxothiazolidinone ring moieties into their structures. Following the synthesis of arylidene thioxothiazolidin‐4‐one derivatives, we embarked on evaluating their effectiveness against a range of cancer cell lines. Our results indicated that several compounds, including c‐Myc‐i7, c‐Myc‐i8, and c‐Myc‐i10, exhibited remarkable reductions in cell viability across various cancer types, including lung, pancreatic, prostate, and breast cancers. Notably, c‐Myc‐i7 demonstrated exceptional selectivity towards breast cancer and DU‐145 prostate cancer cells. These findings underscore the potential of c‐Myc‐i7 as a promising therapeutic agent for the treatment of these cancers. Moreover, our computational analysis delved into the molecular and druglikeness characteristics of the synthesized compounds. These analyses revealed that c‐Myc‐i7 exhibited a substantially higher binding affinity to the Myc‐Max complex compared to the reference compound, 10058‐F4. The structural clustering of the compounds provided valuable insights into their relationships and interactions, enhancing our understanding of their properties and potential modes of action. This study comprehensively assessed the IC50 values of c‐Myc inhibitors across diverse cancer and healthy cell lines, unveiling their therapeutic potential. Remarkably, c‐Myc‐i7 emerged as a standout candidate, displaying exceptional efficacy against various cancer types. Notably, in A549 lung cancer cells, c‐Myc‐i10 outperformed the positive control, 10058‐F4, while c‐Myc‐i7 exhibited similar efficacy. DU‐145 prostate cancer cells showed an extraordinary 40‐fold increase in sensitivity to c‐Myc‐i7 compared to 10058‐F4, with c‐Myc‐i10 also demonstrating significant improvements. Additionally, in MCF7 breast cancer cells, both c‐Myc‐i7 and c‐Myc‐i10 exhibited substantial efficacy enhancements over 10058‐F4. Importantly, these findings extended to pancreatic cancer cells, with c‐Myc‐i10 displaying a four‐fold increase in efficacy. Furthermore, our selectivity analysis highlighted c‐Myc‐i7's impressive specificity for breast cancer and DU‐145 prostate cancer cells, emphasizing its potential as a promising therapeutic agent for these malignancies. These results underscore c‐Myc‐i7's significance and its potential to advance cancer therapeutics.

In conclusion, this study highlights c‐Myc‐i7 as a promising c‐Myc inhibitor with significant efficacy against multiple cancer types and exceptional selectivity. Additionally, our findings underscore the importance of minimizing molecular weight in drug design to enhance drug absorption and efficacy. The synthesis of novel compounds with varying levels of potency opens up exciting possibilities for the development of potent therapeutic agents, paving the way for further investigations in diverse cancer models and phenotypic assays, which may lead to the discovery of novel and highly effective compounds in cancer therapy.

## AUTHOR CONTRIBUTIONS

**Arif Mermer:** Conceptualization (equal); data curation (equal); formal analysis (equal); investigation (equal); methodology (equal); writing – original draft (equal); writing – review and editing (equal). **Sümbül Yıldırım:** Data curation (equal); investigation (equal); software (equal); validation (equal); writing – original draft (equal). **Fatih Kocabaş:** Conceptualization (equal); data curation (equal); formal analysis (equal); investigation (equal); methodology (equal); software (equal); validation (equal); writing – original draft (equal); writing – review and editing (equal).

## CONFLICT OF INTEREST STATEMENT

The authors declare that they have no conflicts of interest to disclose.

## Supporting information


Supplementary File 1:

## Data Availability

No data availability.
