# Supplementary material for: Development, synthesis and validation of improved c‐Myc/Max inhibitors
Source: J Cell Mol Med. 2024 Apr 3;28(8):e18272. doi: 10.1111/jcmm.18272 (PMC10989597; doi:10.1111/jcmm.18272)
Supplement: Supplementary file 1 [file JCMM-28-e18272-s001.docx]

**Development, synthesis and validation of improved c-Myc/Max inhibitors**

Sümbül Yıldırım^1,2,3^, Fatih Kocabaş^1,2*^, Arif Mermer^4,5,6*^

^1^Department of Genetics and Bioengineering, Faculty of Engineering, Yeditepe University, Istanbul, Türkiye

^2^Graduate School of Natural and Applied Sciences, Yeditepe University, Istanbul, Türkiye

^3^Graduate School of Natural and Applied Sciences, Yeditepe University, Istanbul, Germany

^4^Department of Biotechnology, University of Health Sciences-Turkey, Uskudar, 34662, Istanbul, Türkiye

^5^Experimental Medicine Application and Research Center, University of Health Sciences-Turkey, Uskudar, 34662, Istanbul, Türkiye

^6^UR22722, LABCİS, Faculty of Science and Technology, University of Limoges, F-87000, Limoges, France

*Co-Correspondence:

Fatih Kocabas, Email: [fatih.kocabas@yeditepe.edu.tr](mailto:fatih.kocabas@yeditepe.edu.tr)

Arif Mermer, Email: [arif.mermer@sbu.edu.tr](mailto:arif.mermer@sbu.edu.tr)

**FTIR, NMR and MALDI-TOF/MS spectra of the compounds**

**
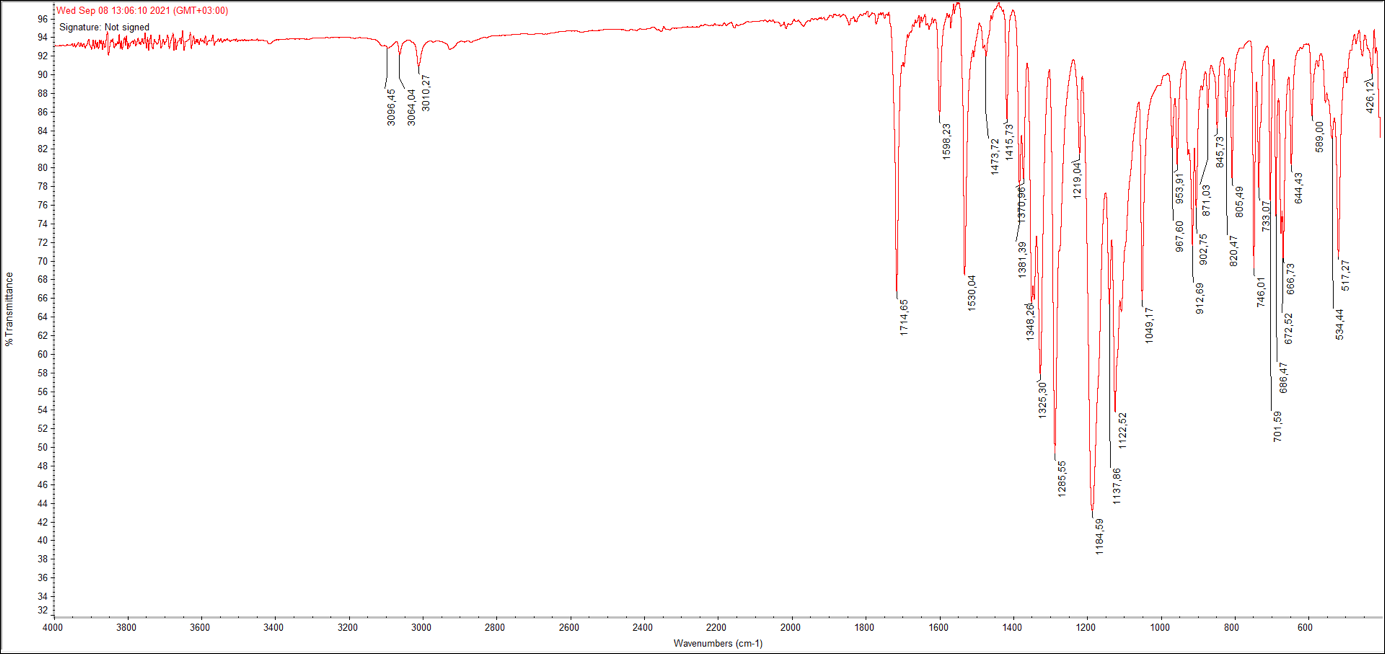
**

FTIR spectra of compound **5a**

**
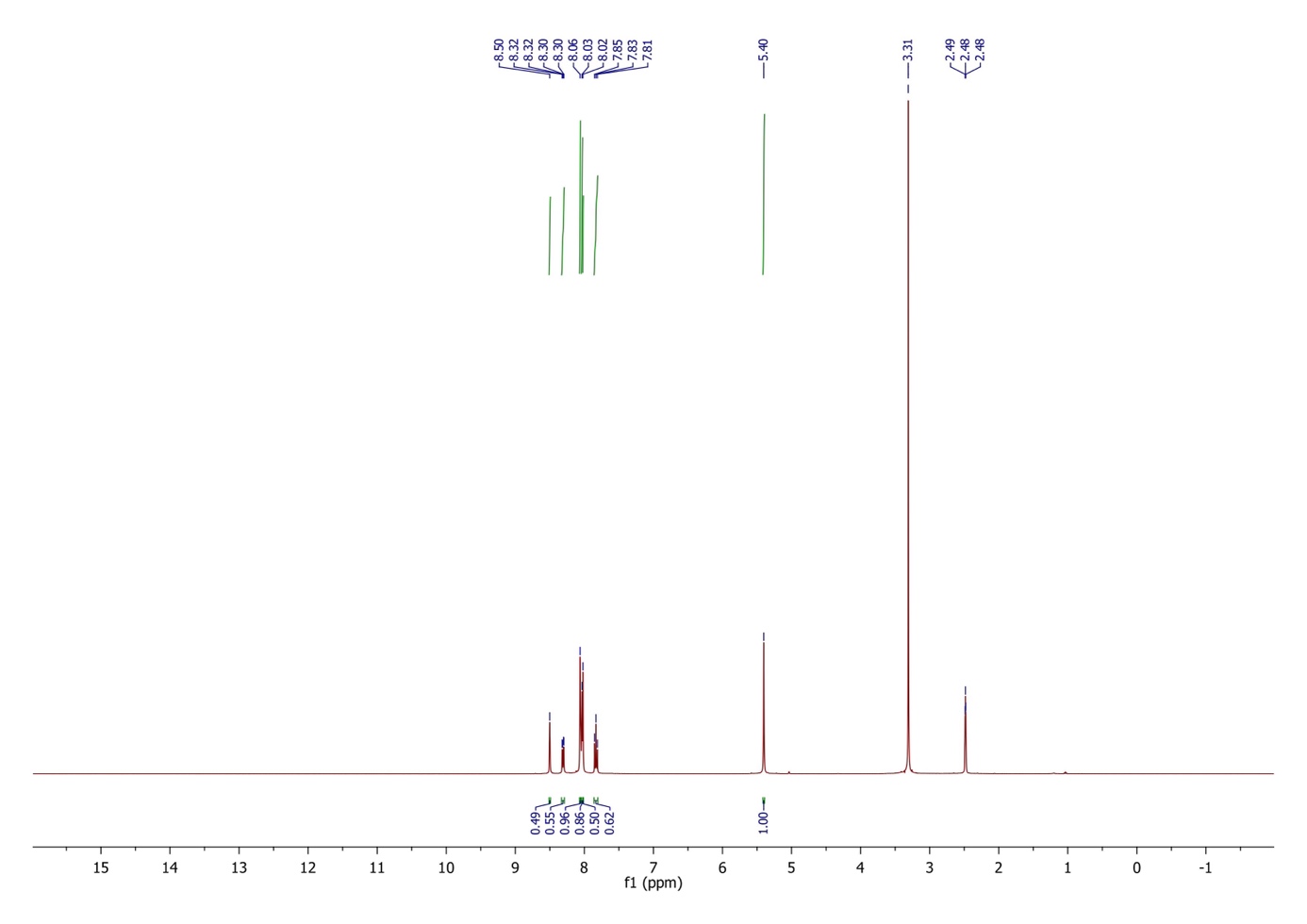
**

^1^H NMR spectra of compound **5a**

**
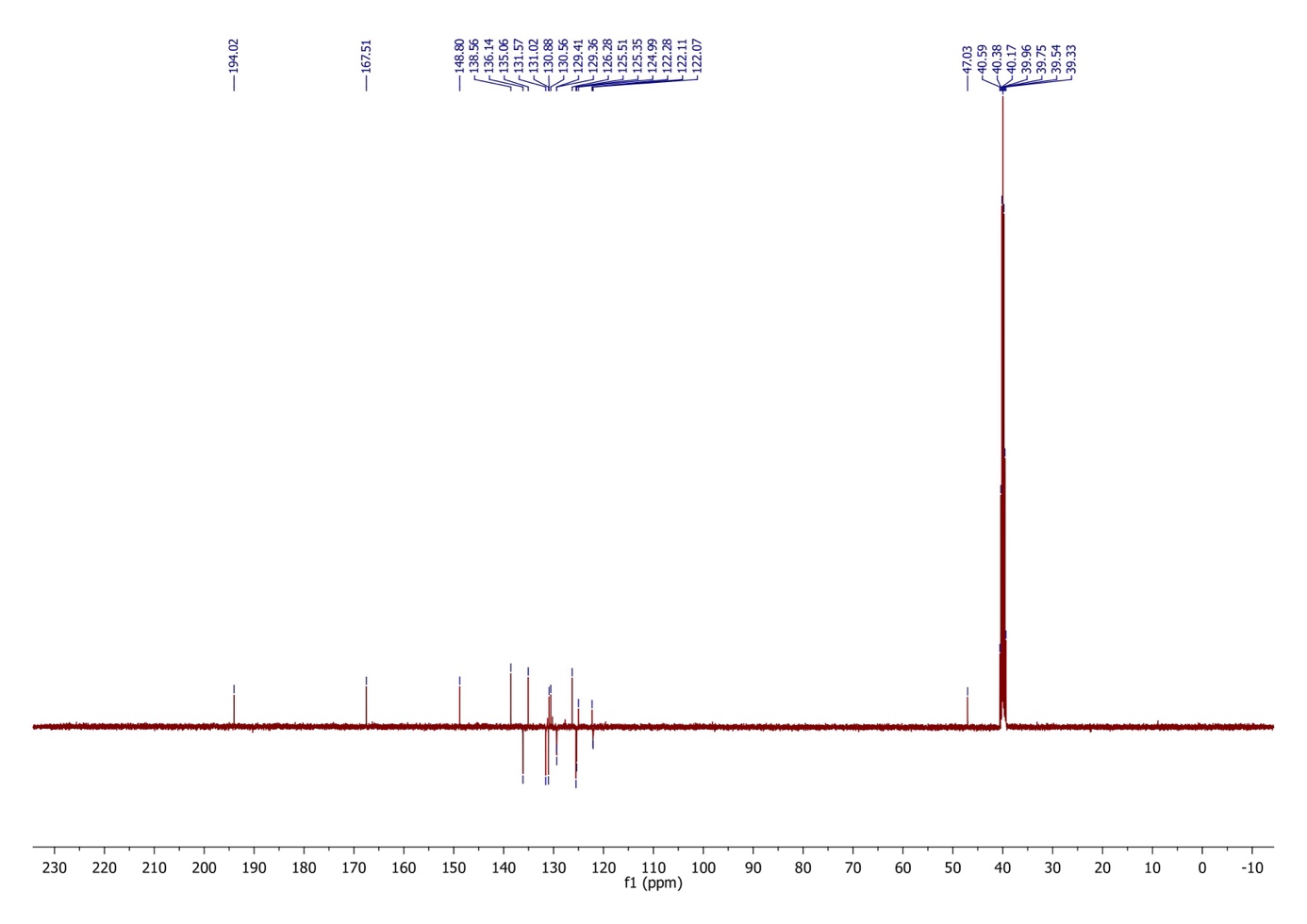
**

^13^C NMR (APT) spectra of compound **5a**

**
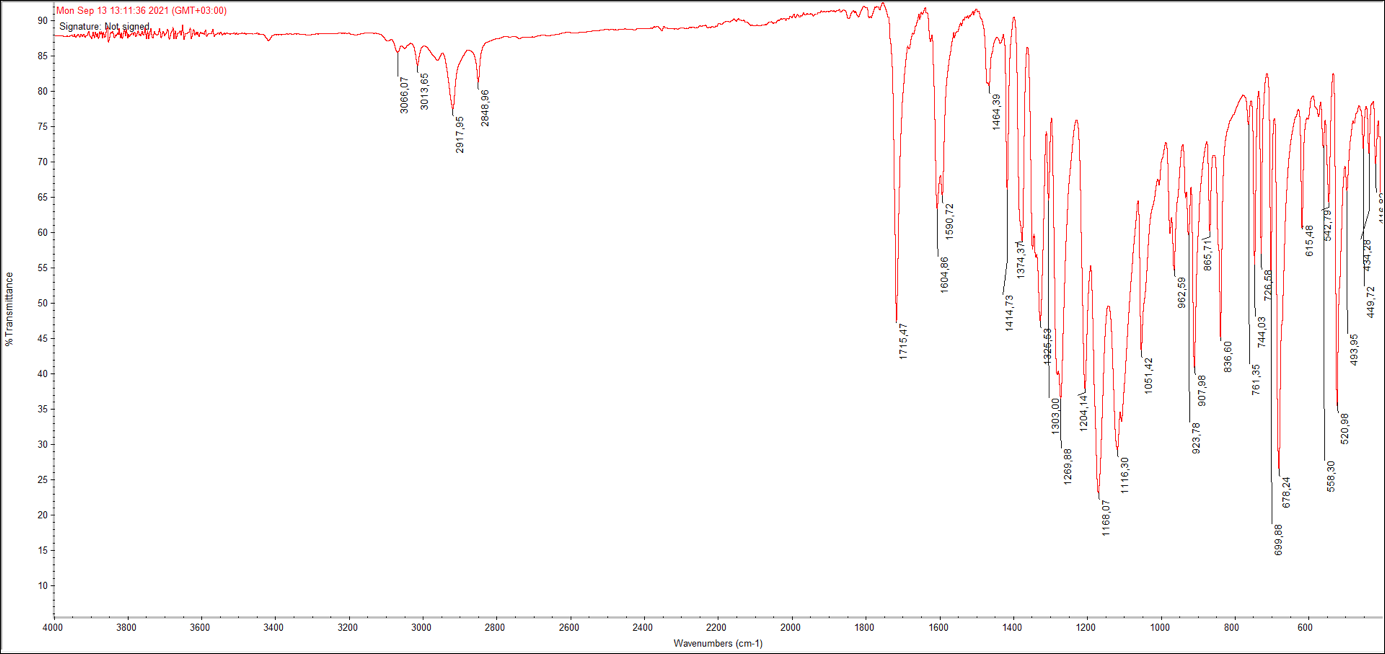
**

FTIR spectra of compound **5b**

**
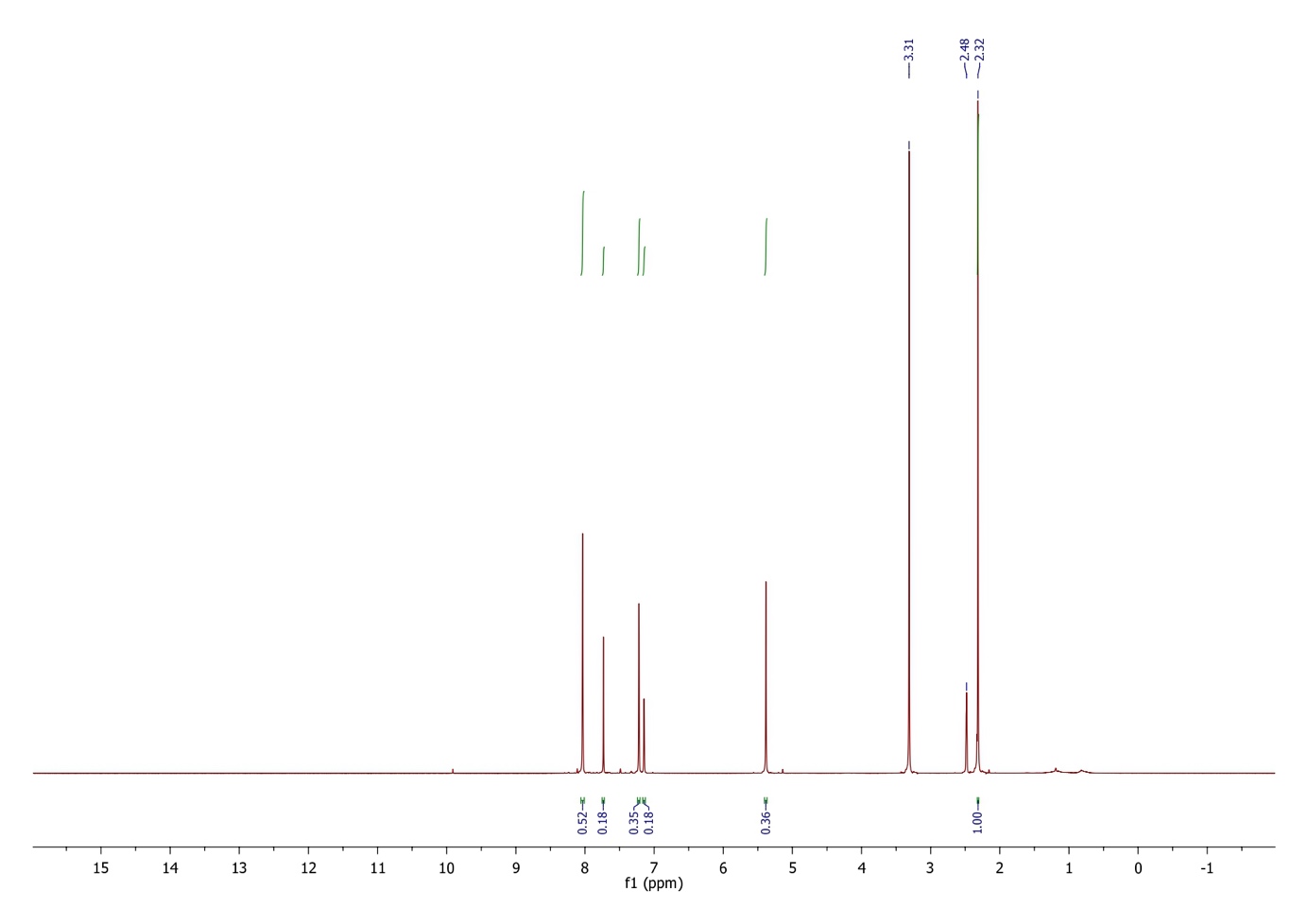
**

^1^H NMR spectra of compound **5b**

**
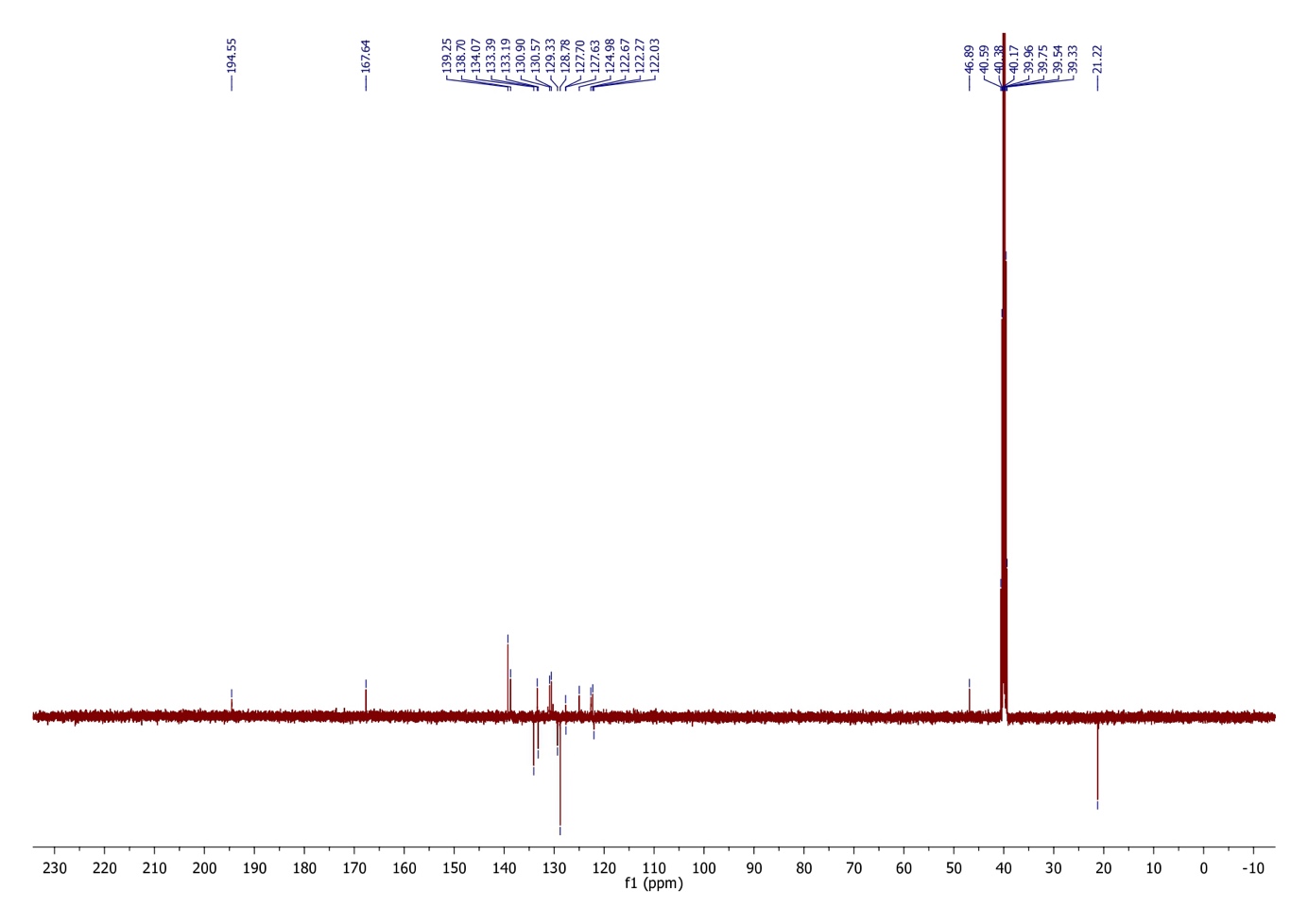
**

^13^C NMR (APT) spectra of compound **5b**

**
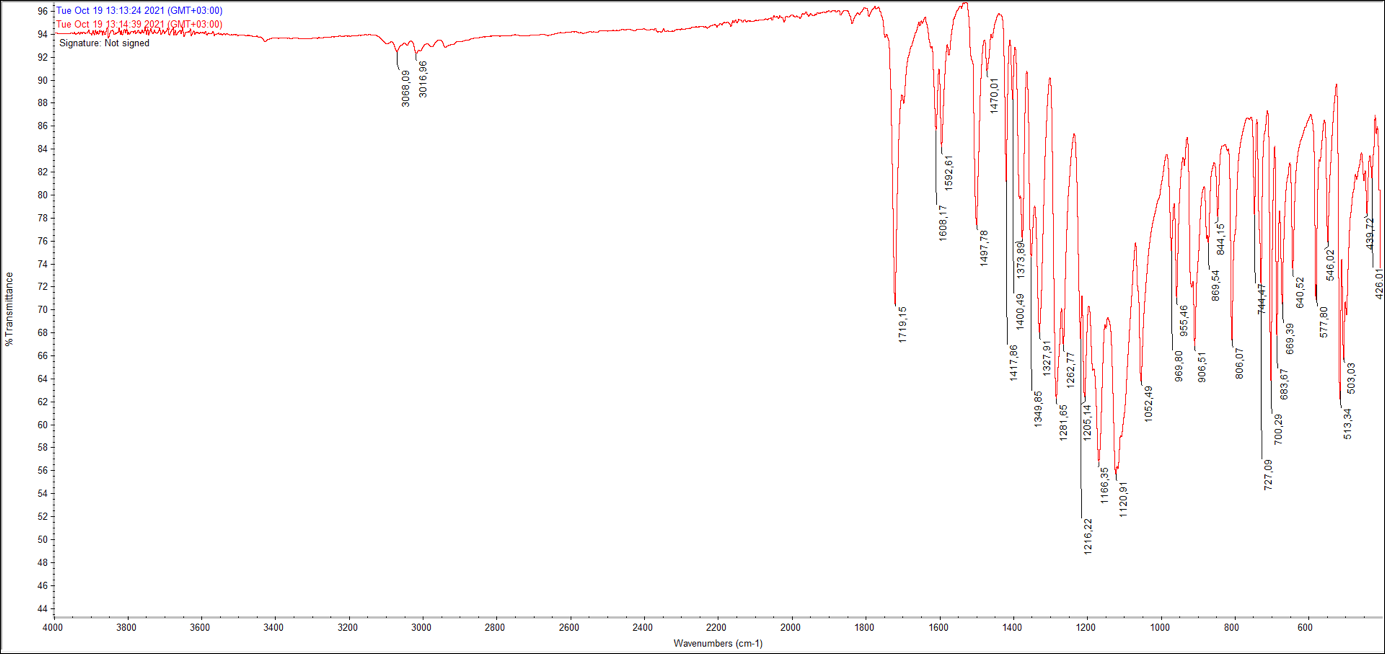
**

FTIR spectra of compound **5c**

**
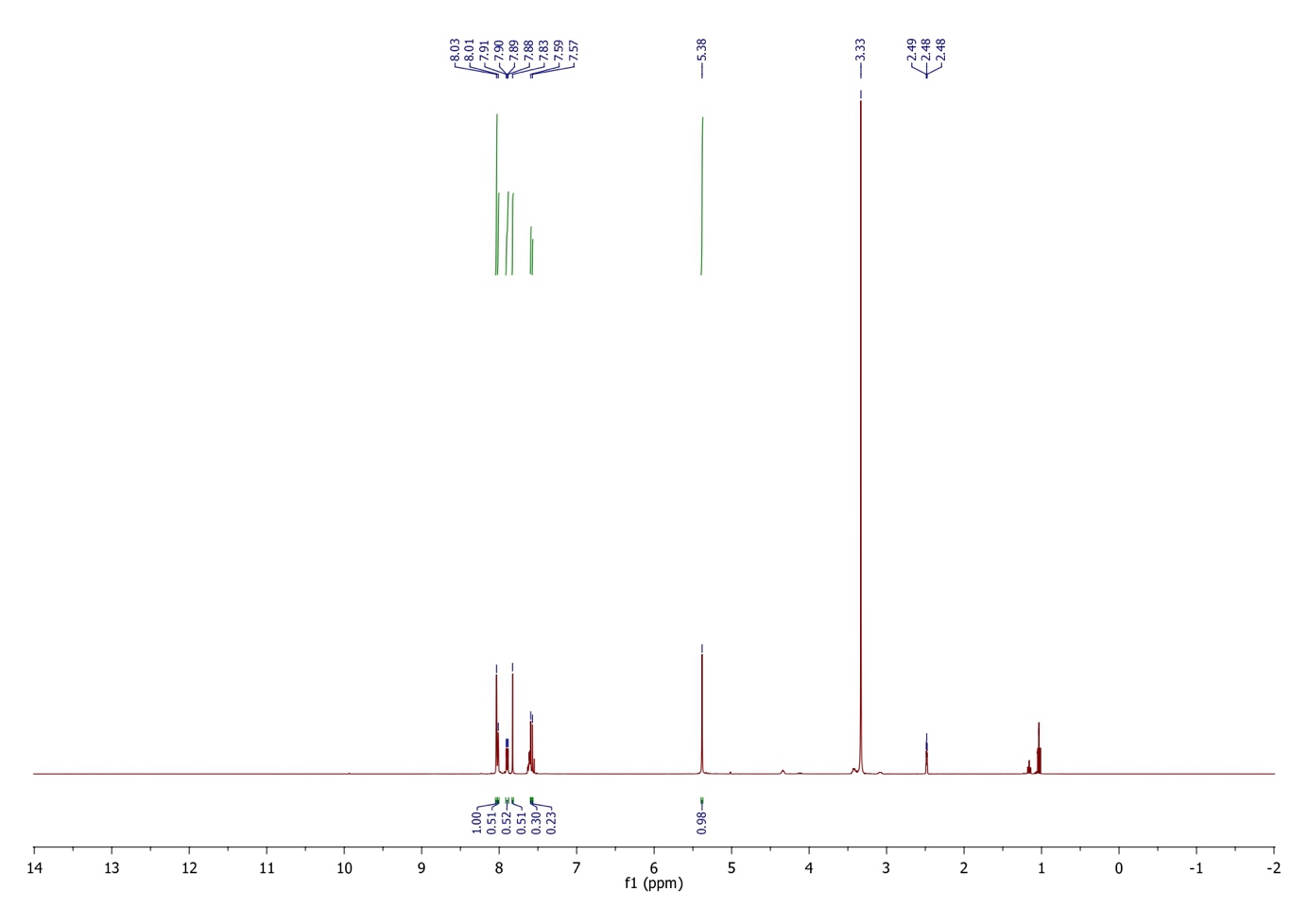
**

^1^H NMR spectra of compound **5c**

**
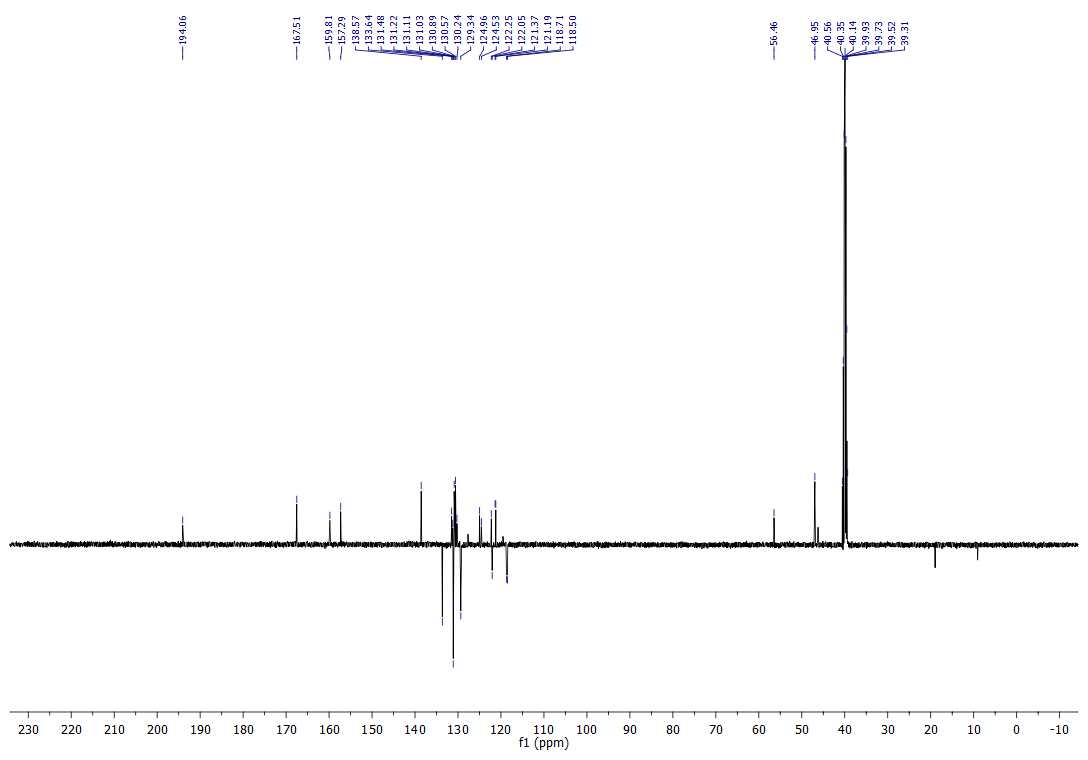
**^13^C NMR (APT) spectra of compound **5c**

**
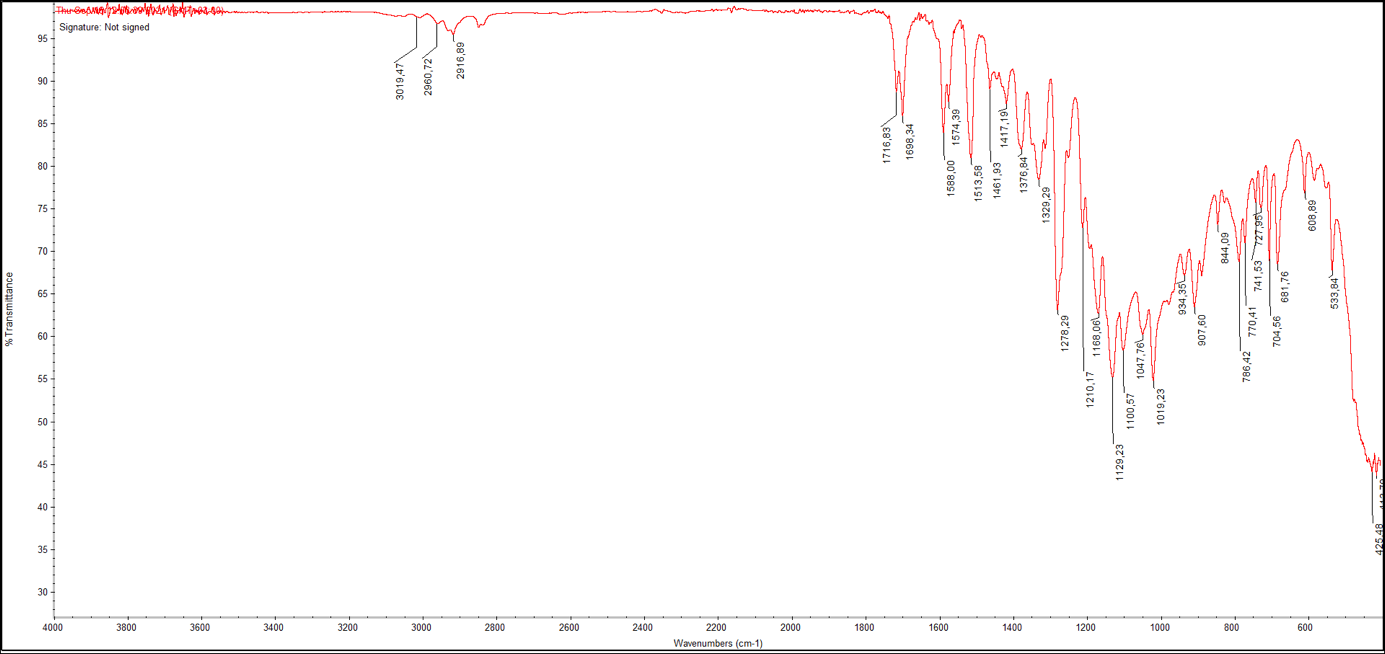
**

FTIR spectra of compound **5d**

**
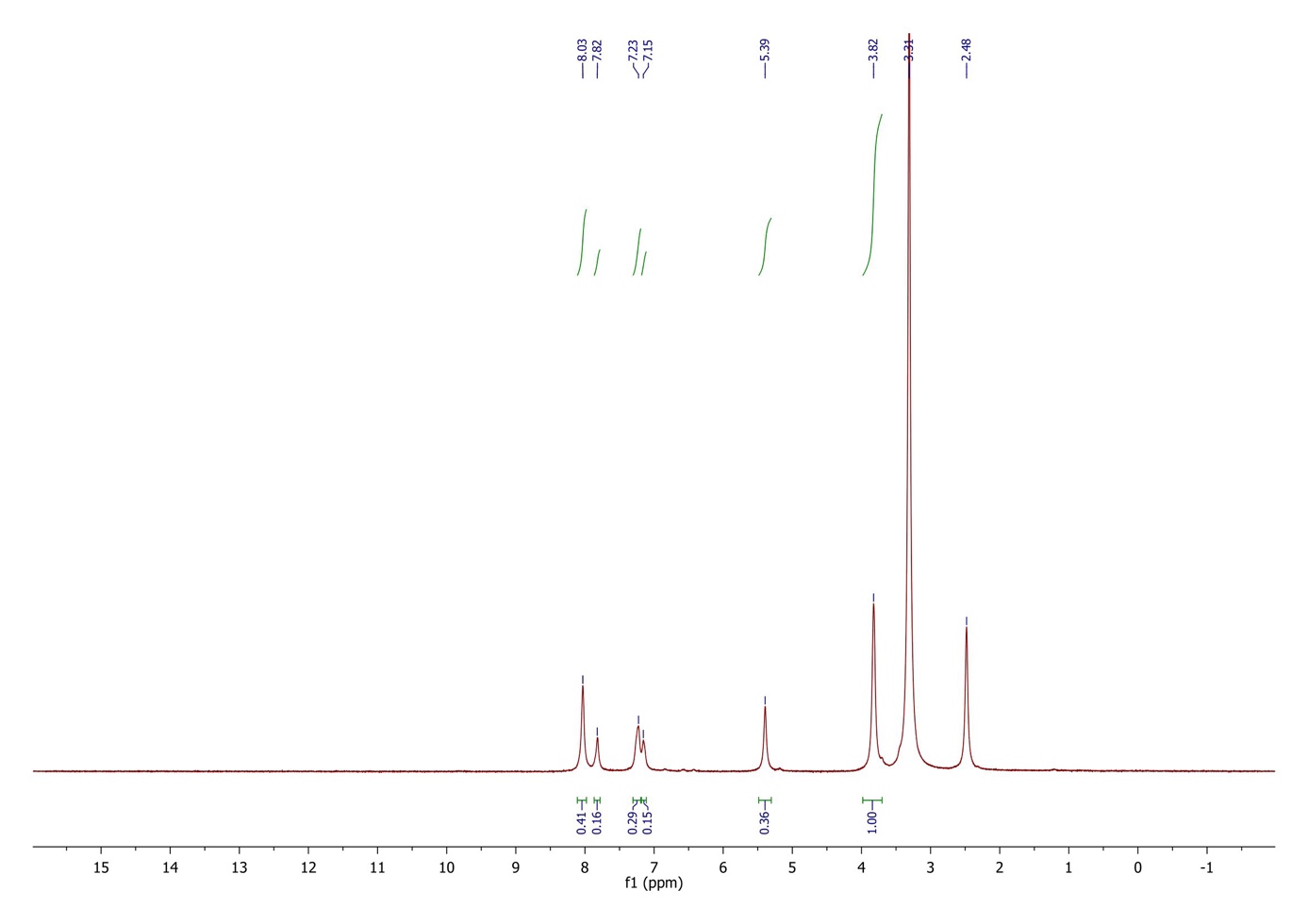
**

^1^H NMR spectra of compound **5d**

**
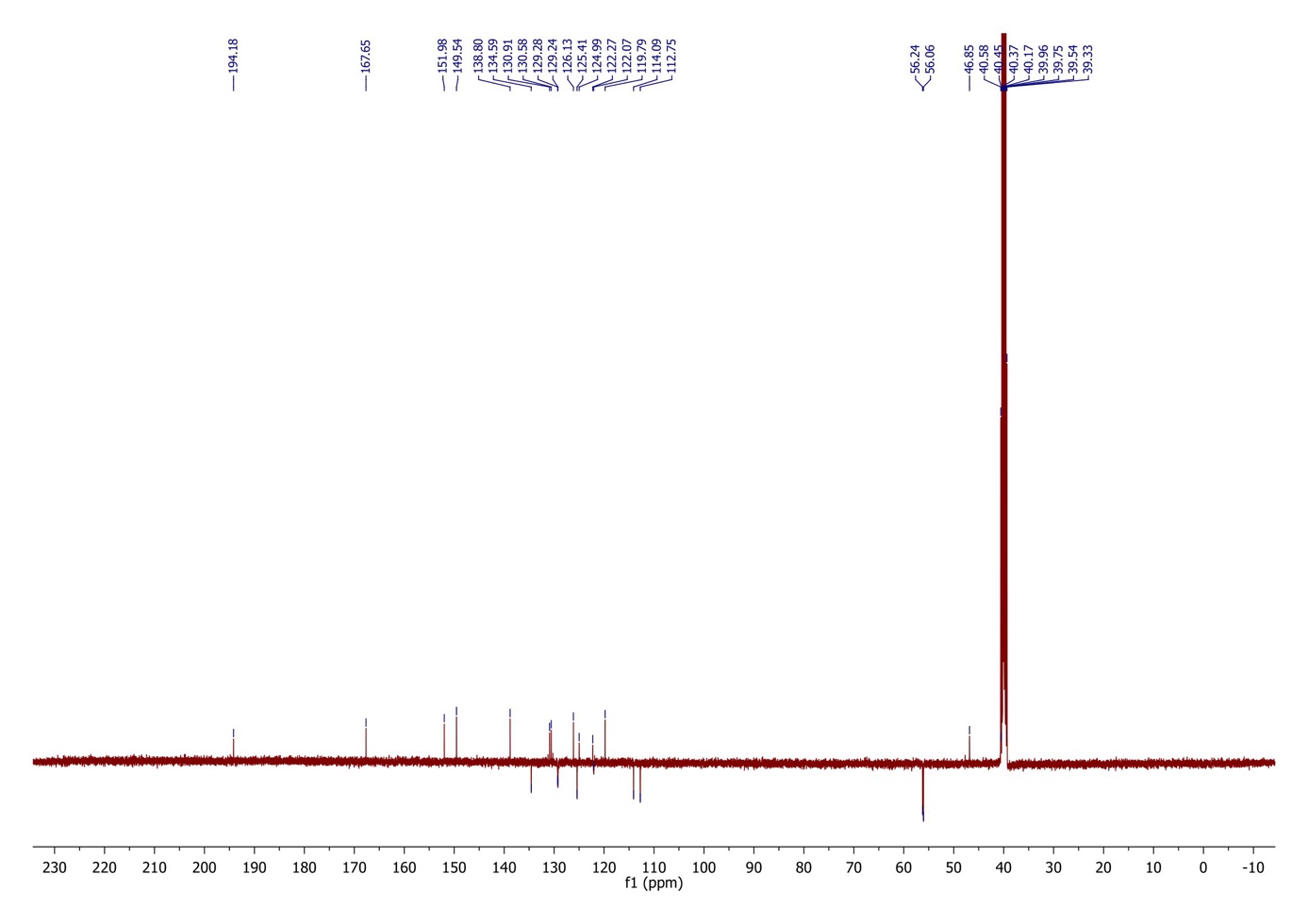
**

^13^C NMR (APT) spectra of compound **5d**

**
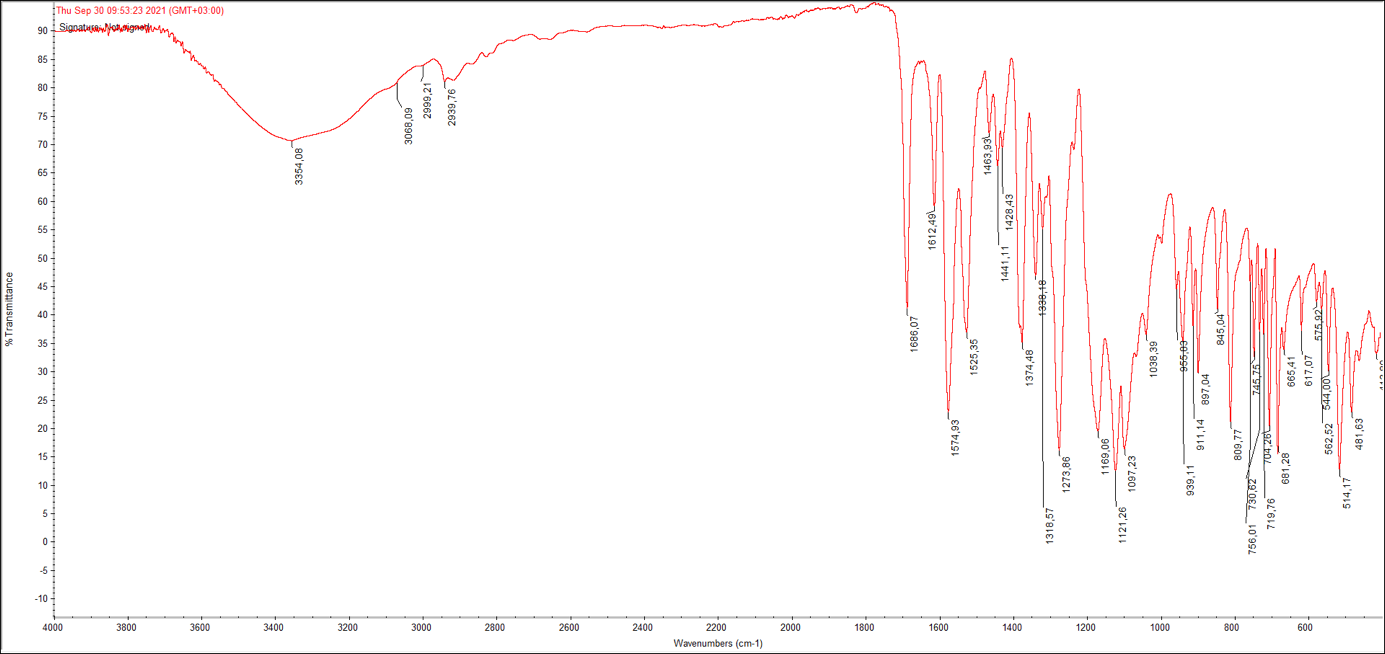
**

FTIR spectra of compound **5e**

**
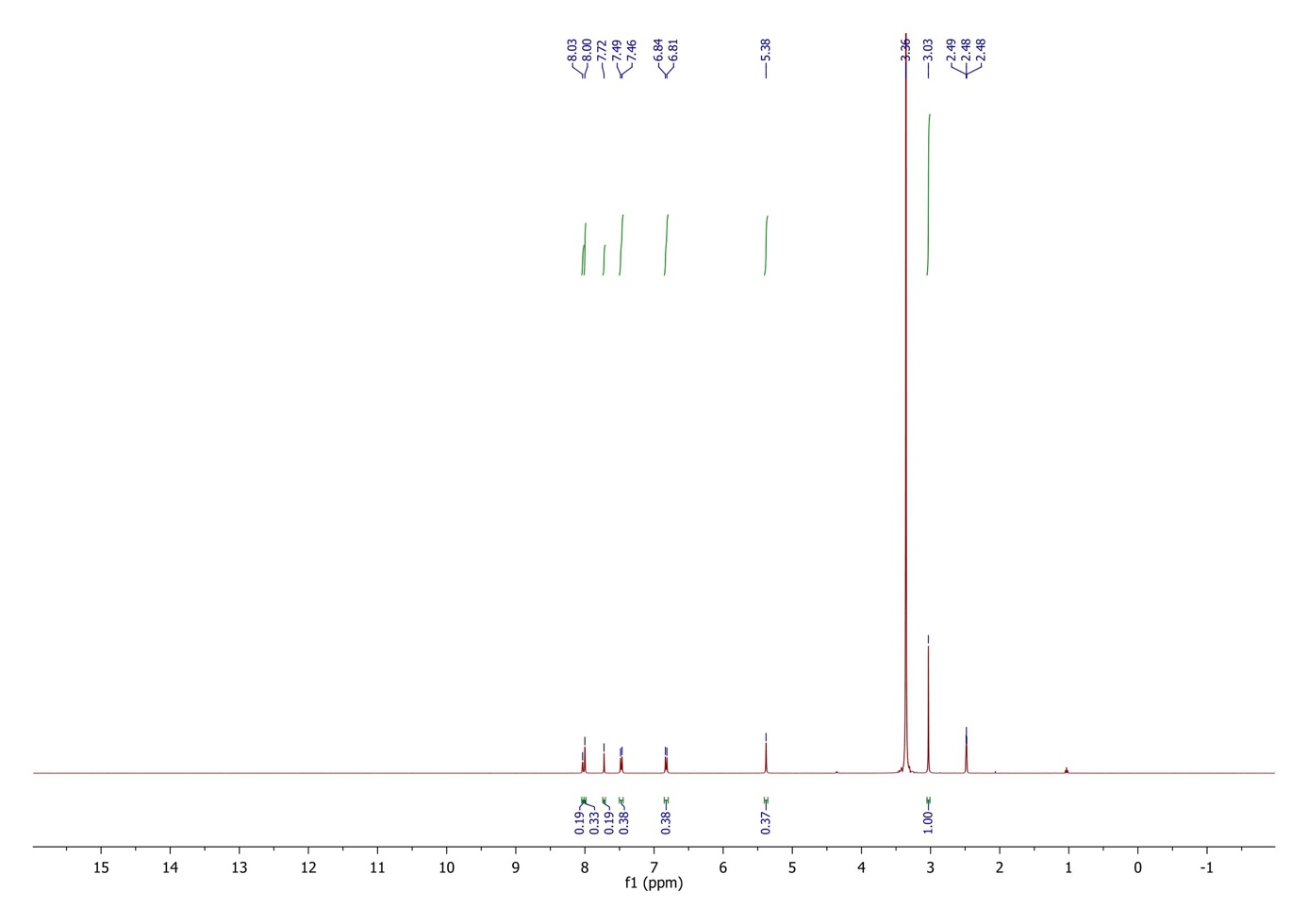
**

^1^H NMR spectra of compound **5e**

**
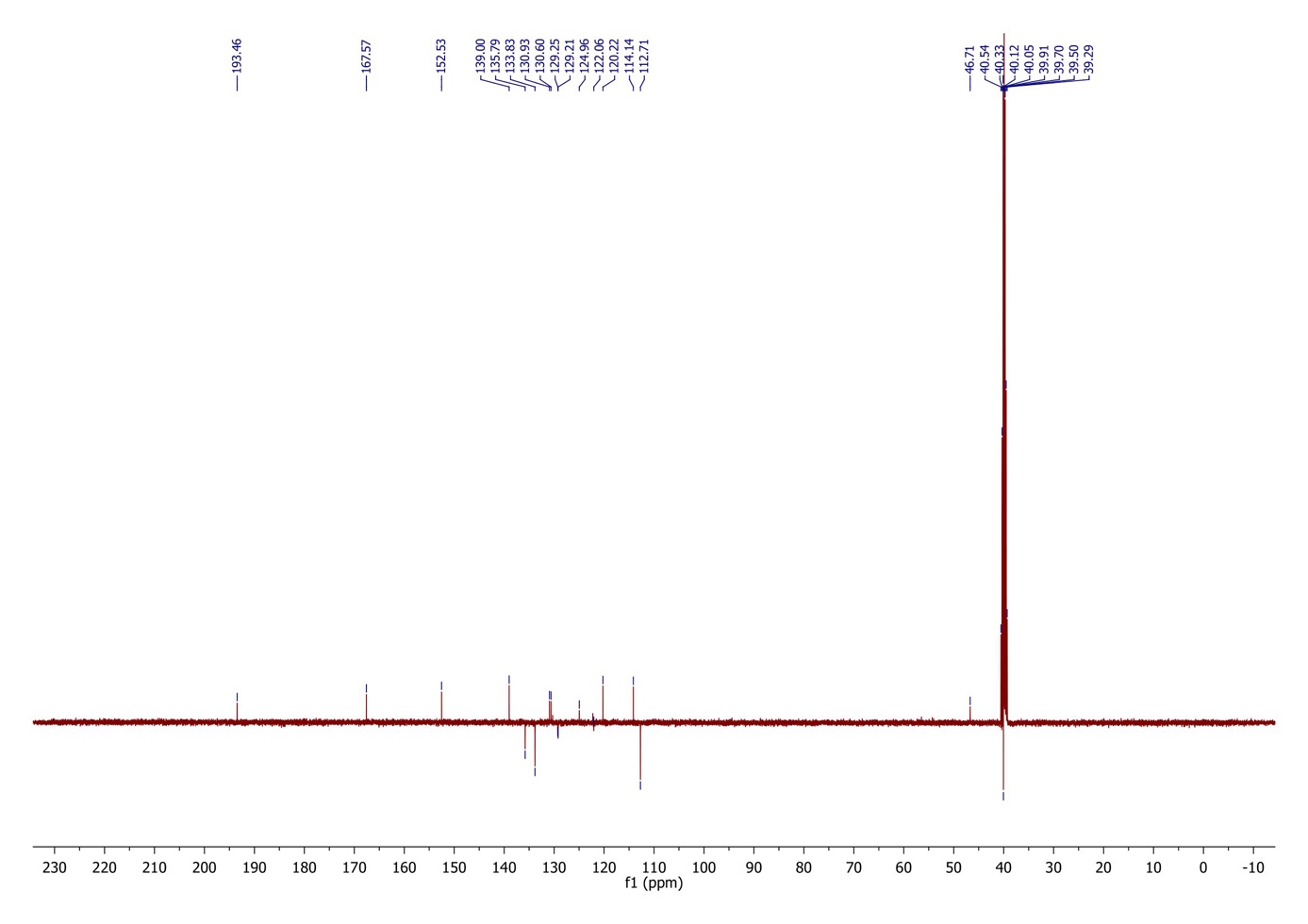
**

^13^C NMR (APT) spectra of compound **5e**

**
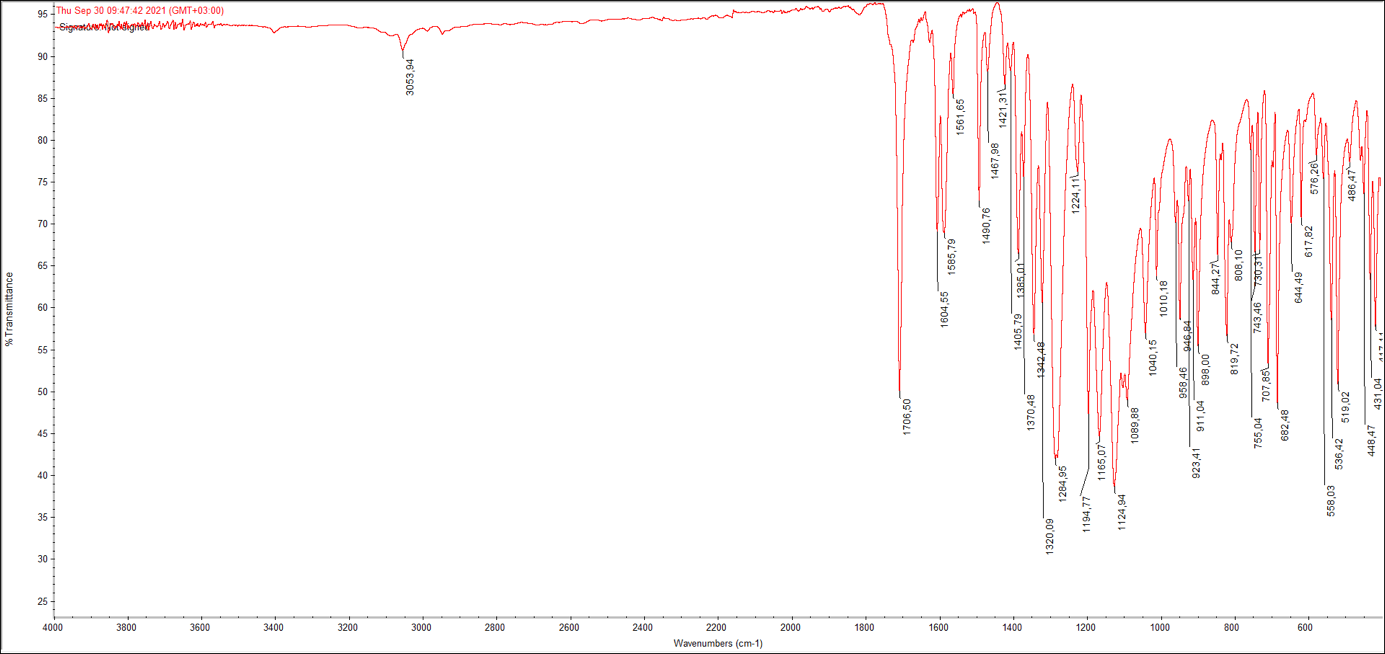
**

FTIR spectra of compound **5f**

**
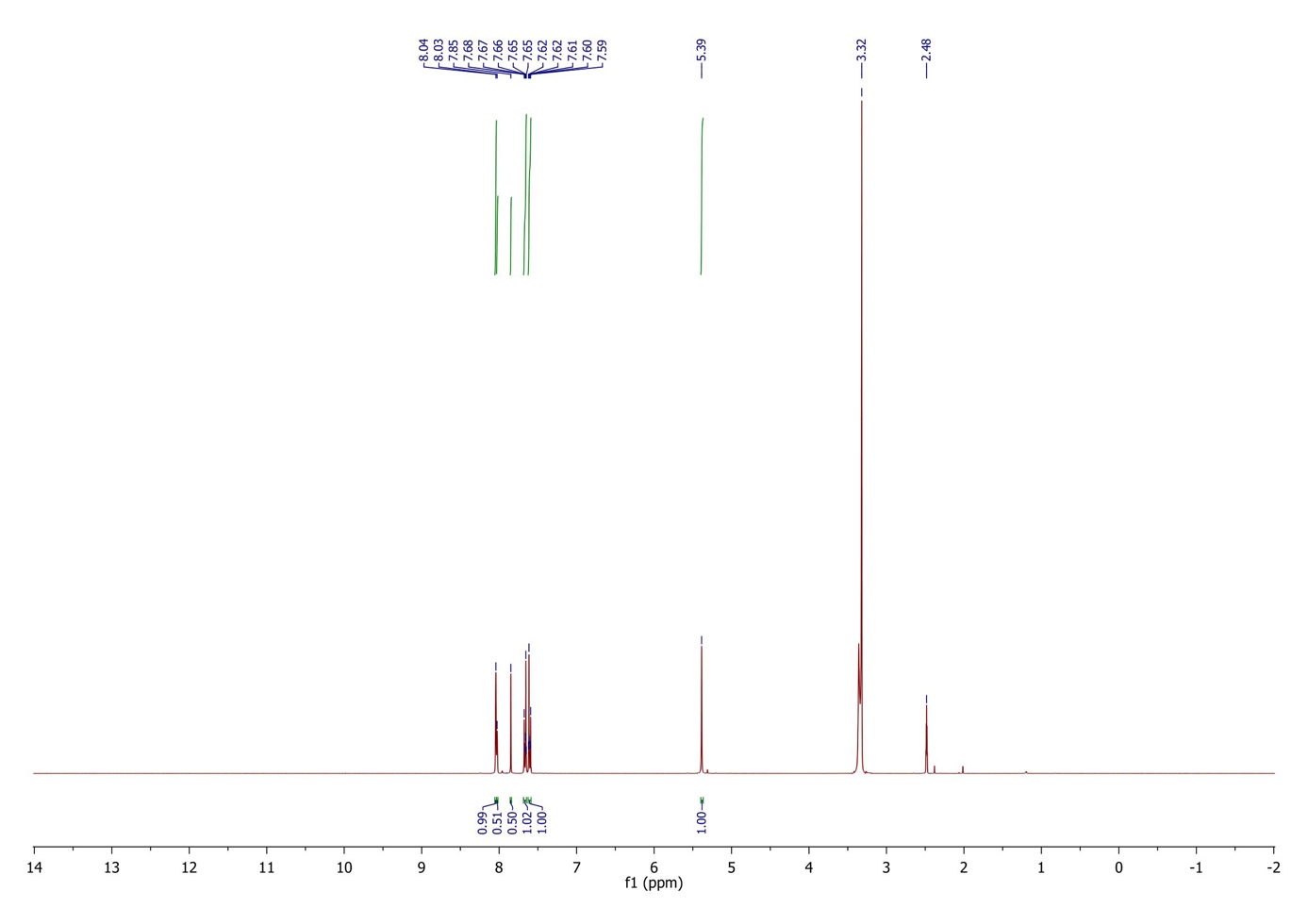
**

^1^H NMR spectra of compound **5f**

**
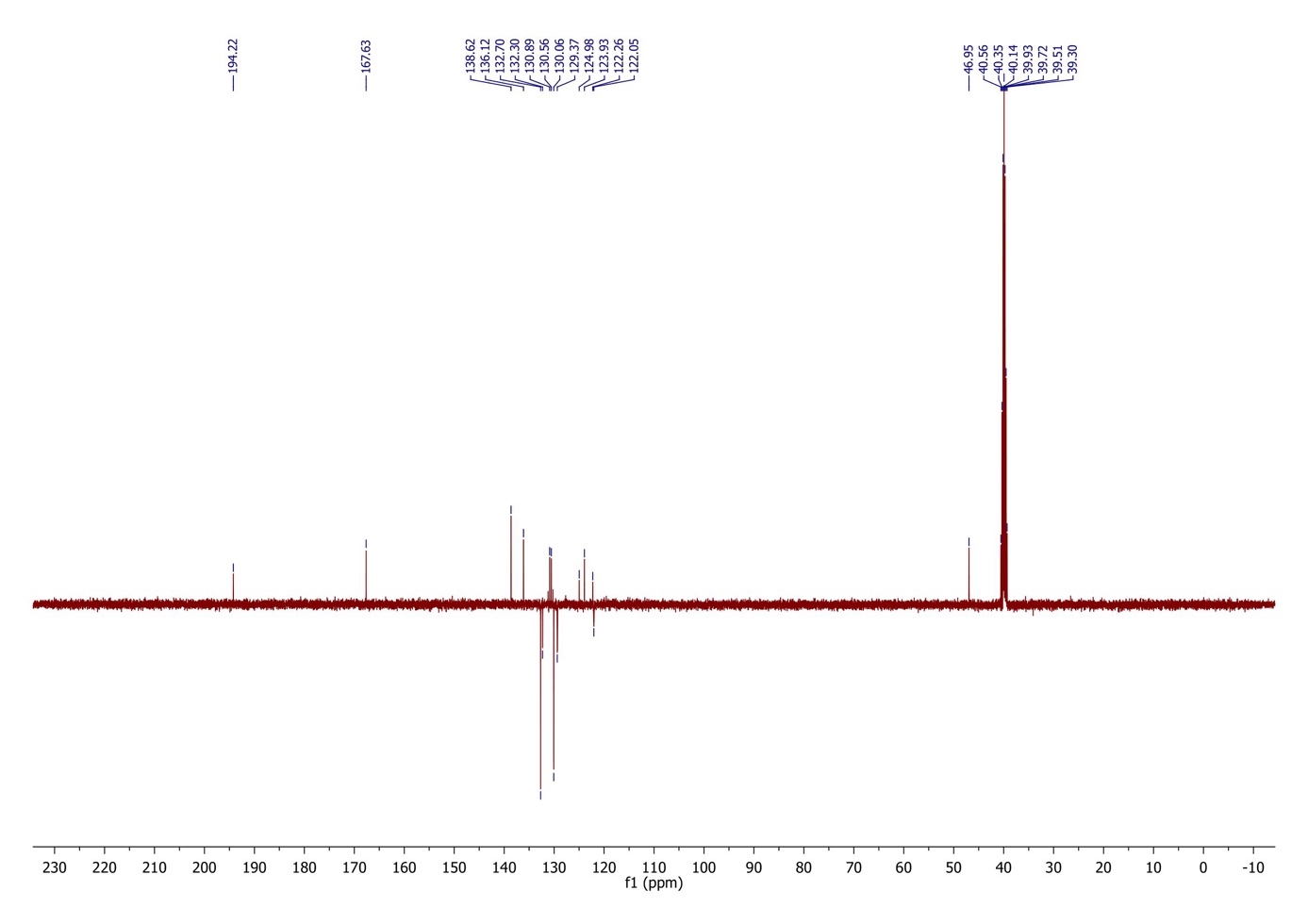
**

^13^C NMR (APT) spectra of compound **5f**

**
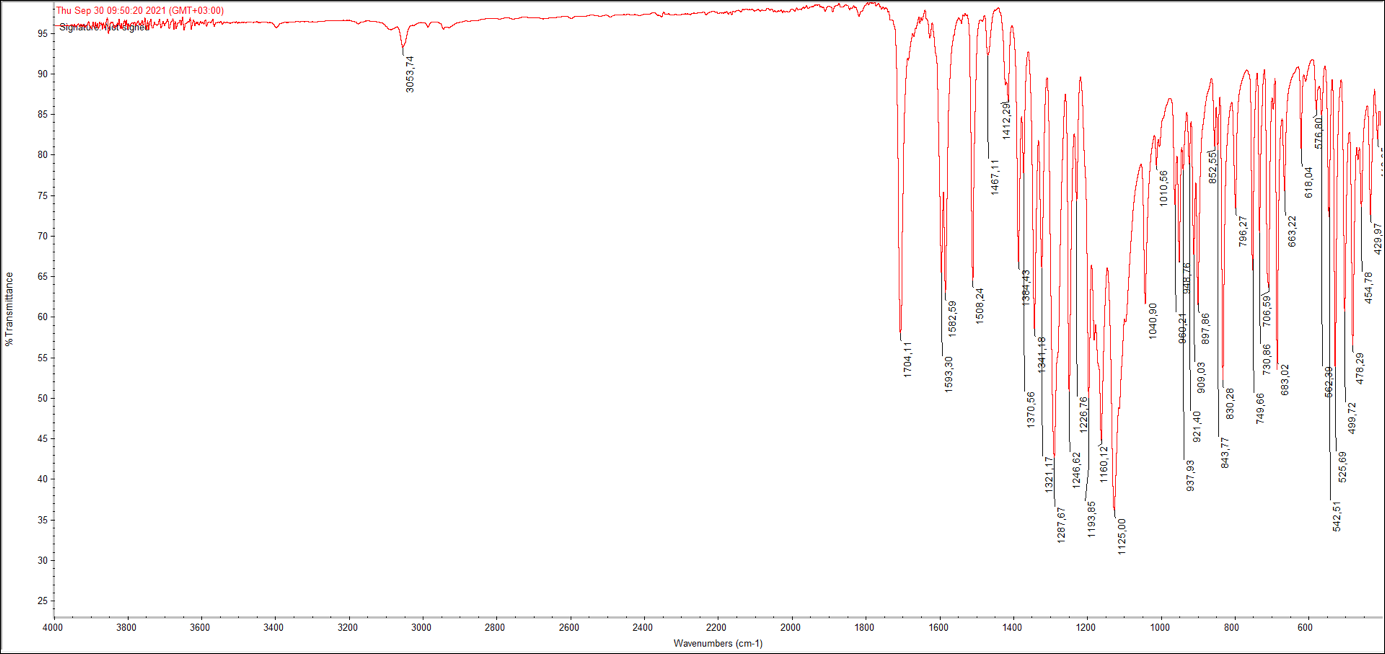
**

FTIR spectra of compound **5g**

**
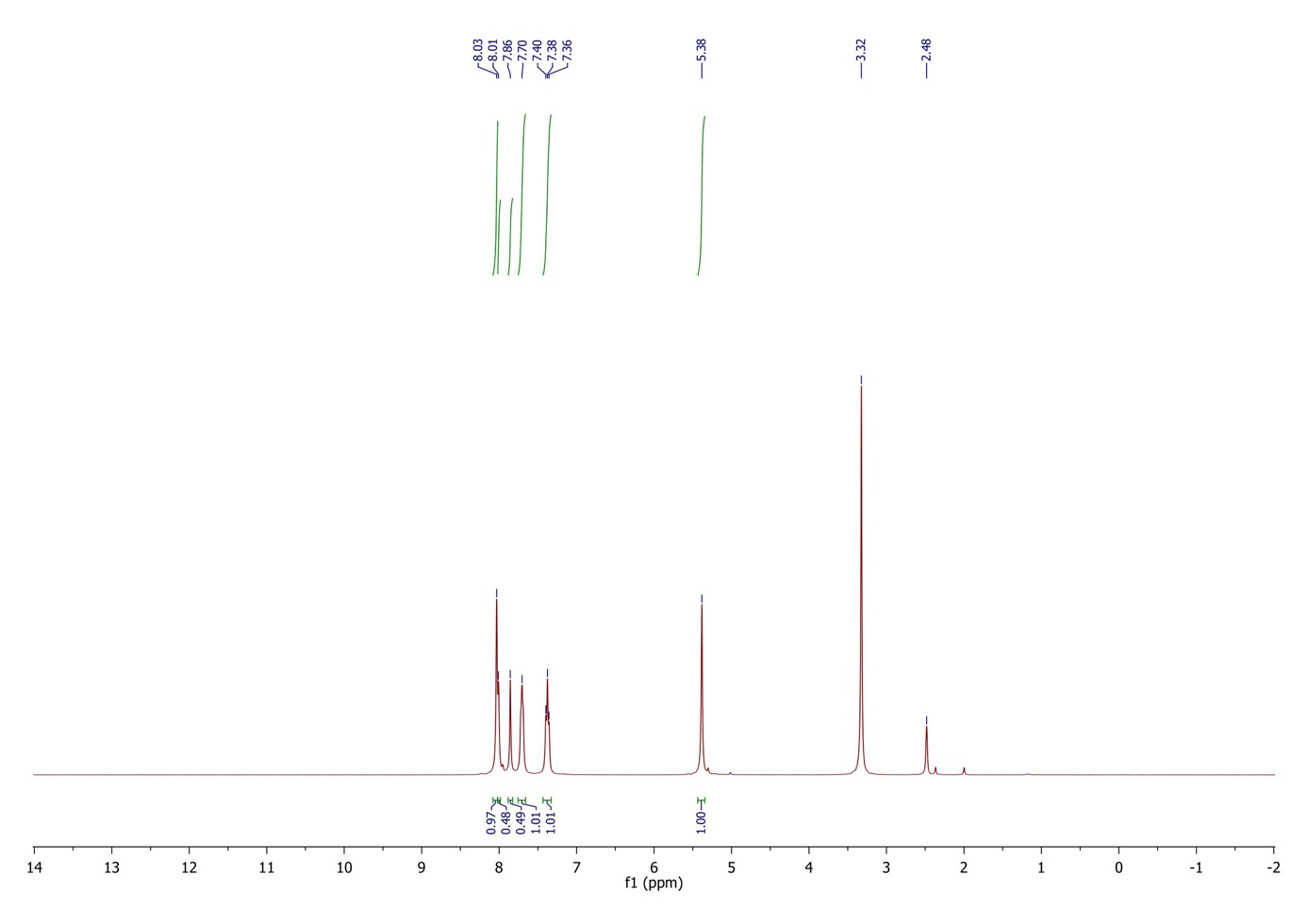
**

^1^H NMR spectra of compound **5g**

**
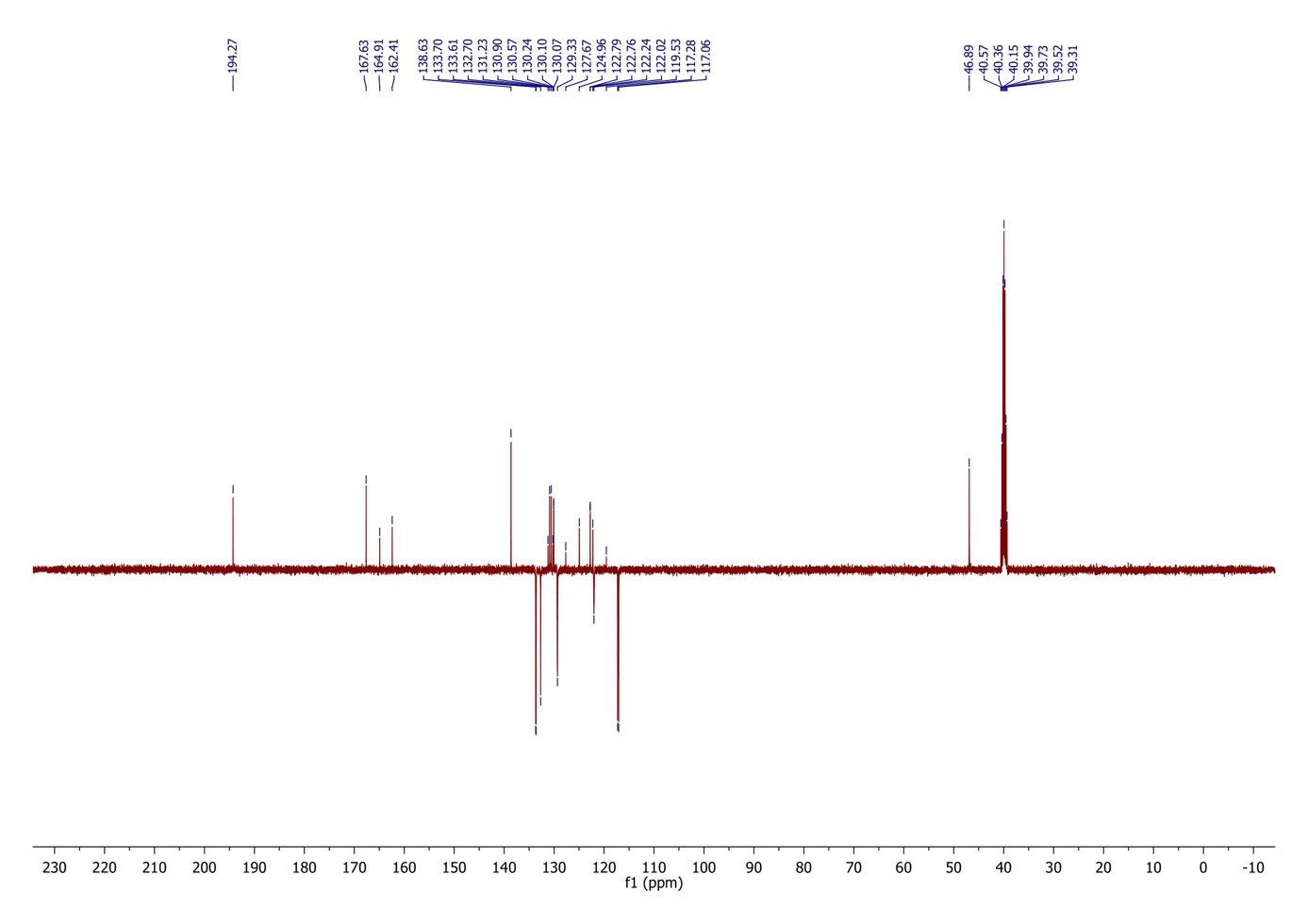
**

^13^C NMR (APT) spectra of compound **5g**

**
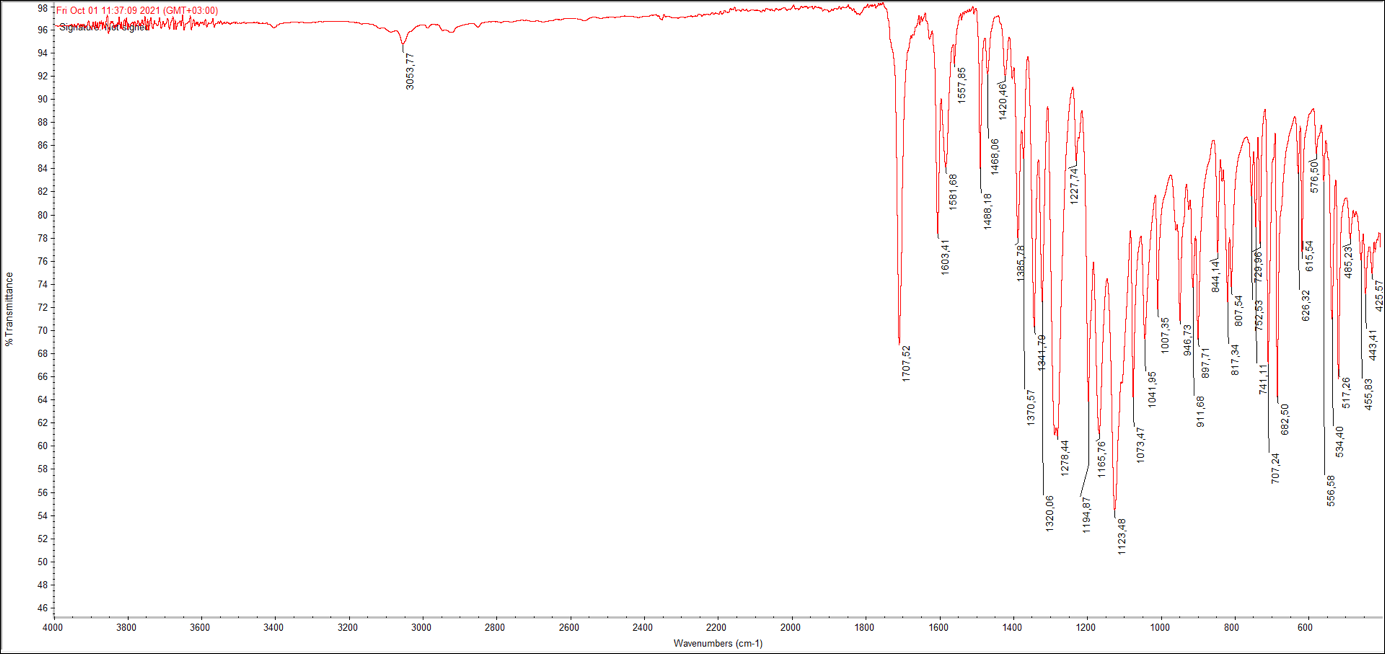
**

FTIR spectra of compound **5h**

**
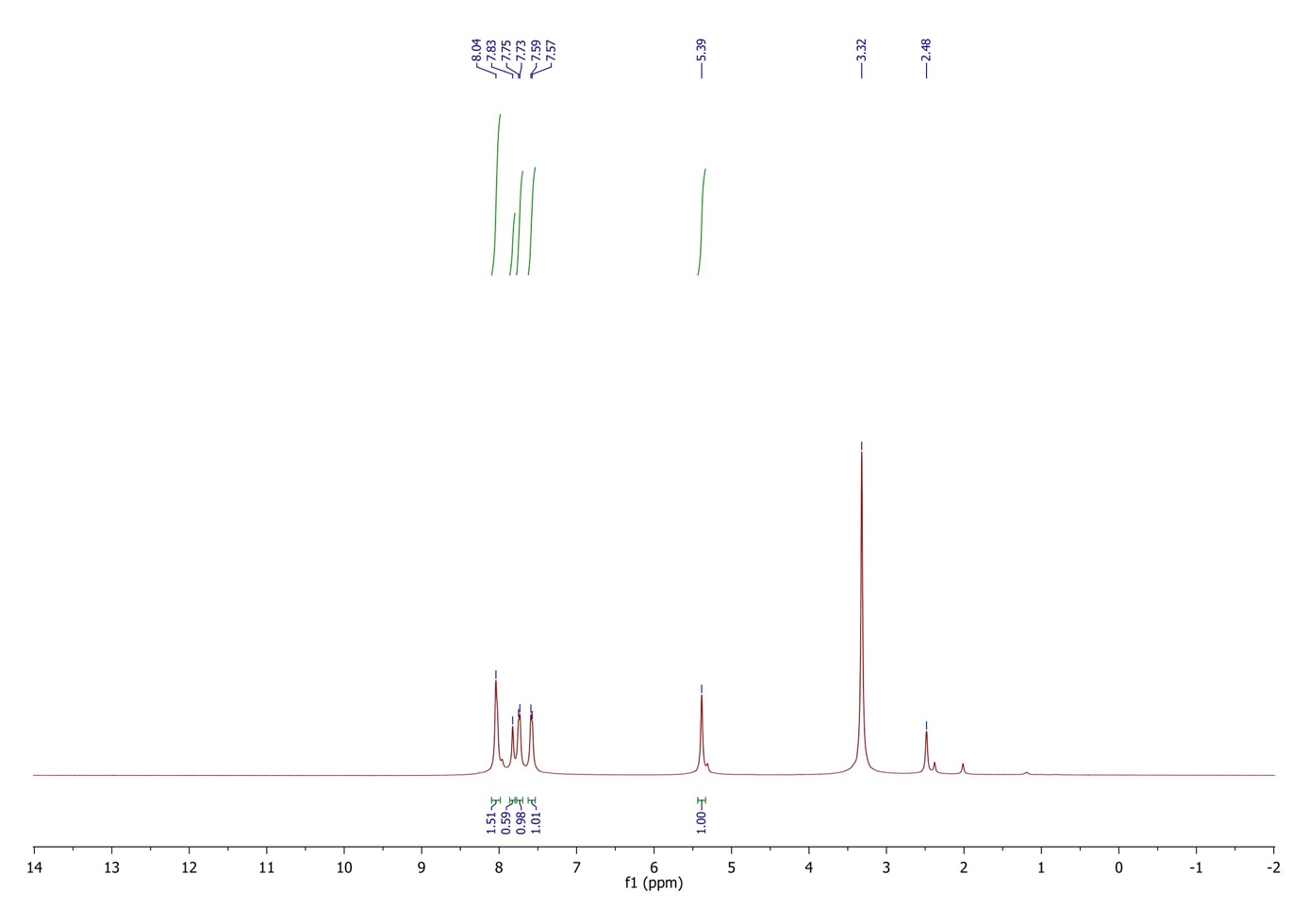
**

^1^H NMR spectra of compound **5h**

**
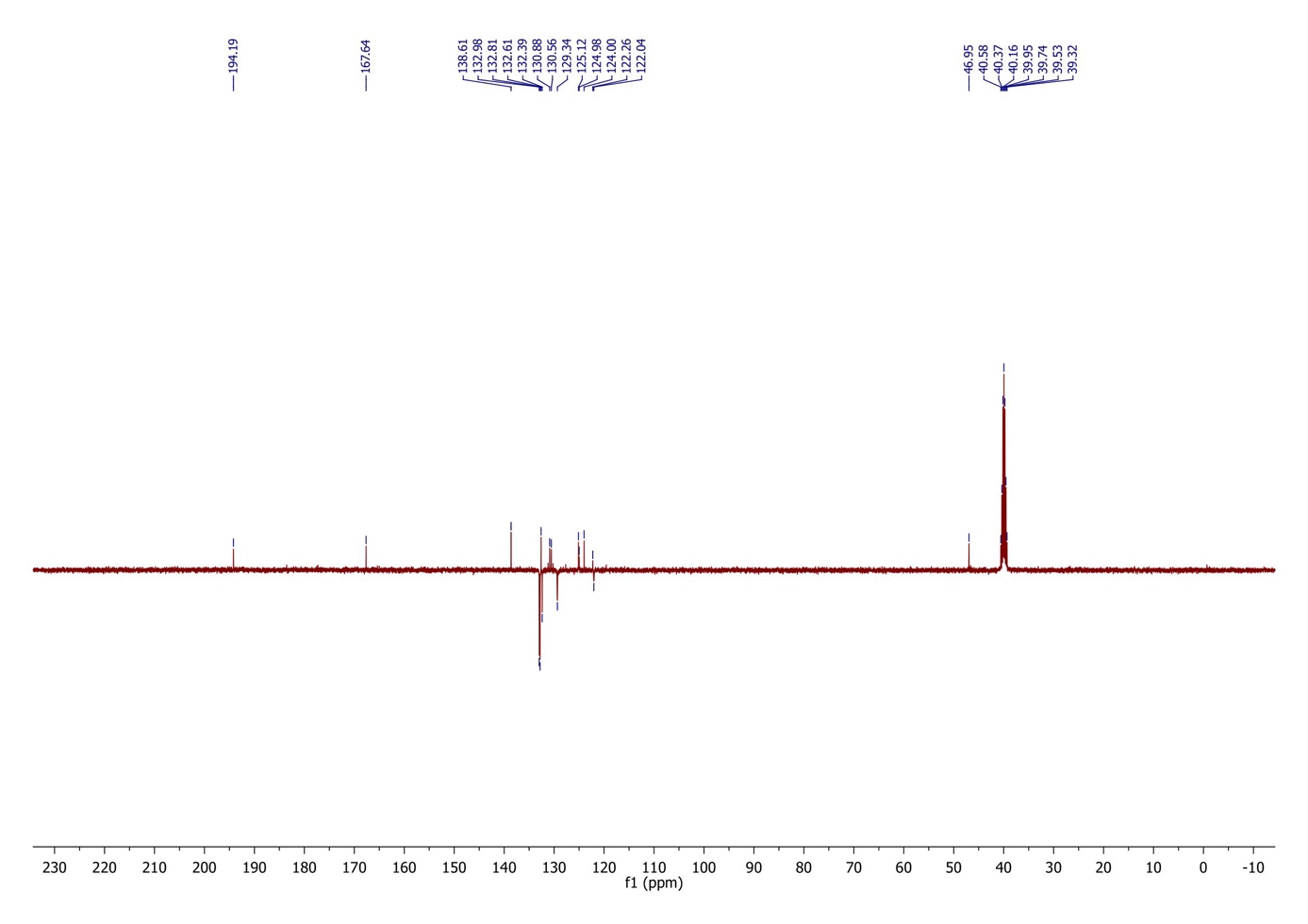
**

^13^C NMR (APT) spectra of compound **5h**

**
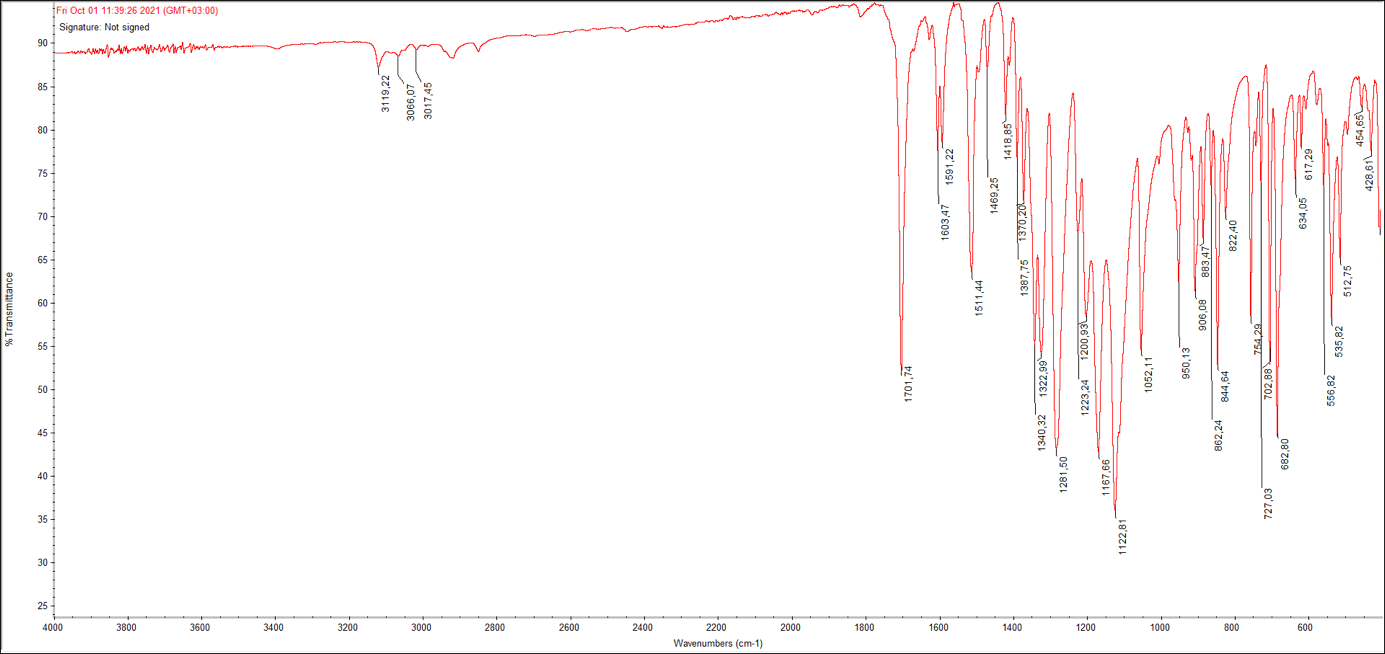
**

FTIR spectra of compound **5i**

**
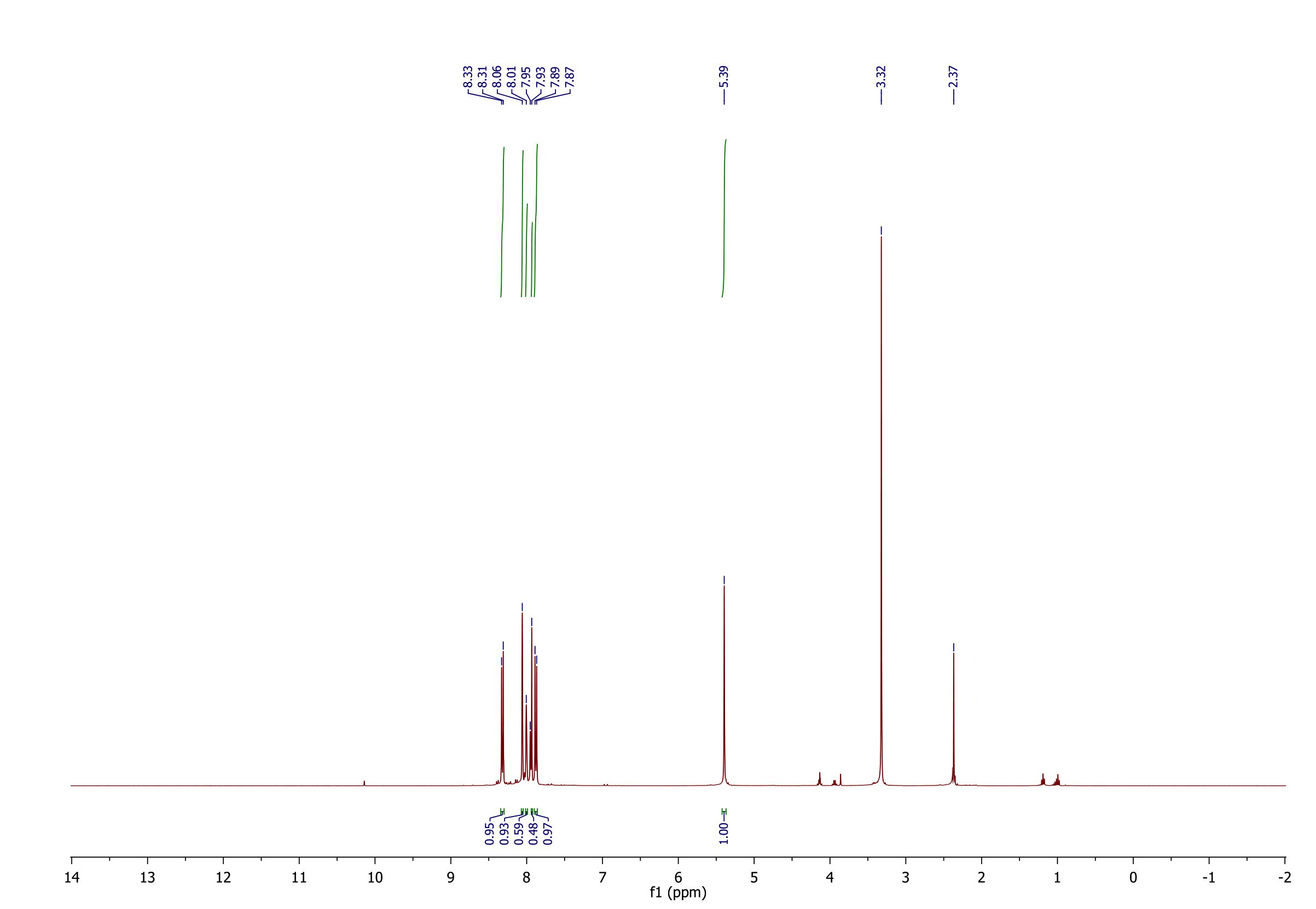
**

^1^H NMR spectra of compound **5i**

**
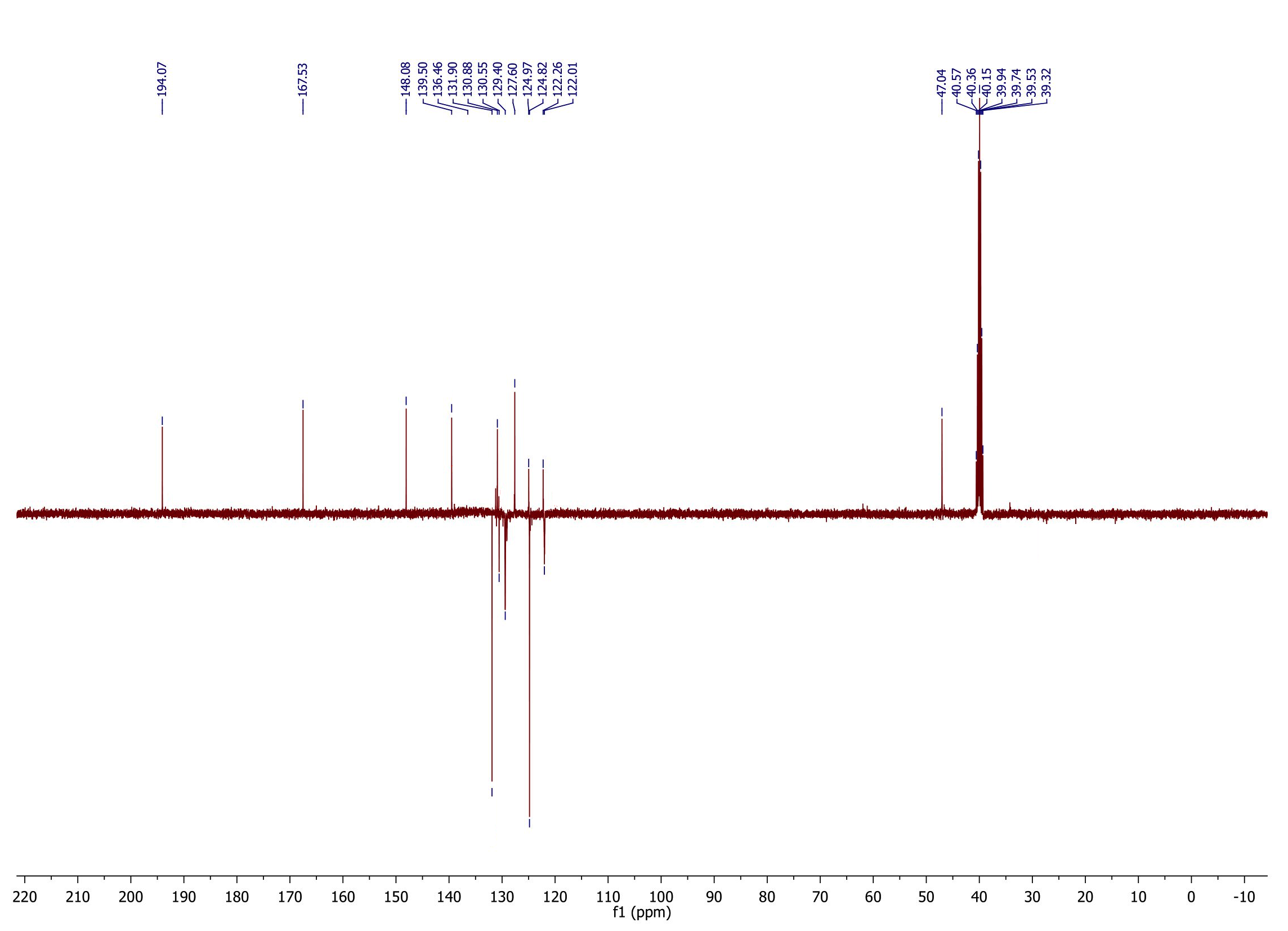
**

^13^C NMR (APT) spectra of compound **5i**

**
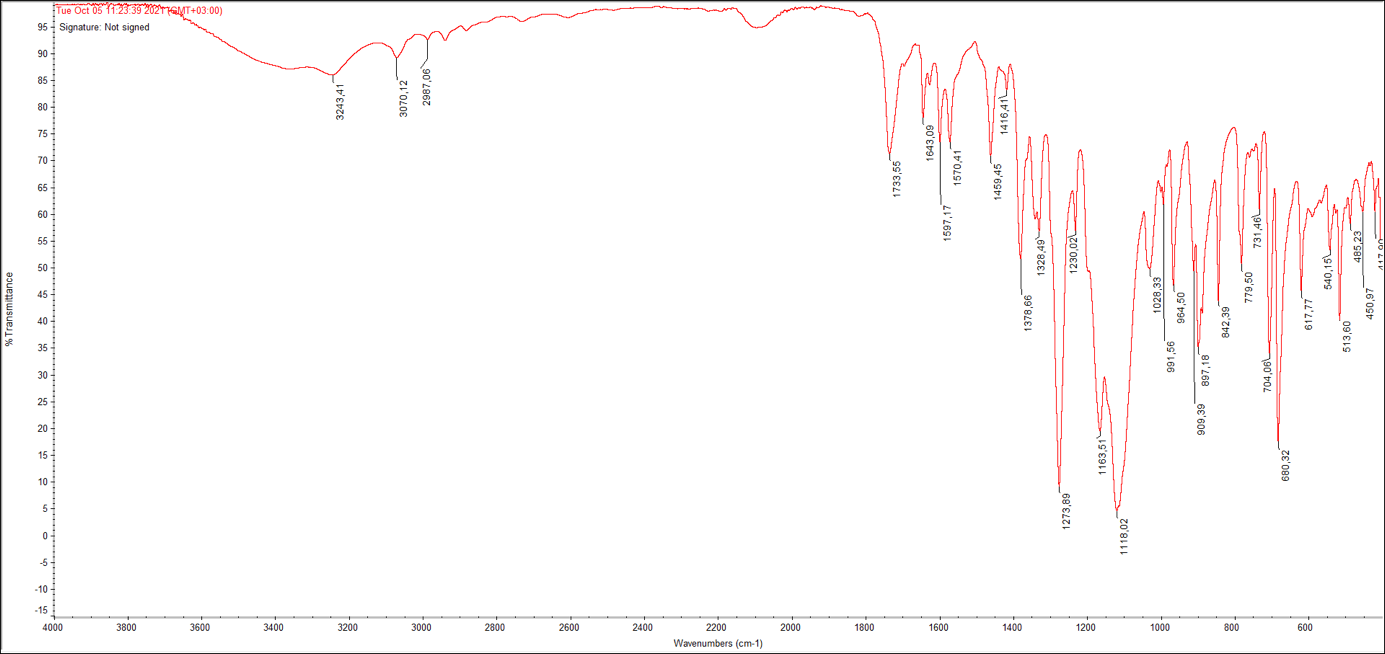
**

FTIR spectra of compound **5j**

**
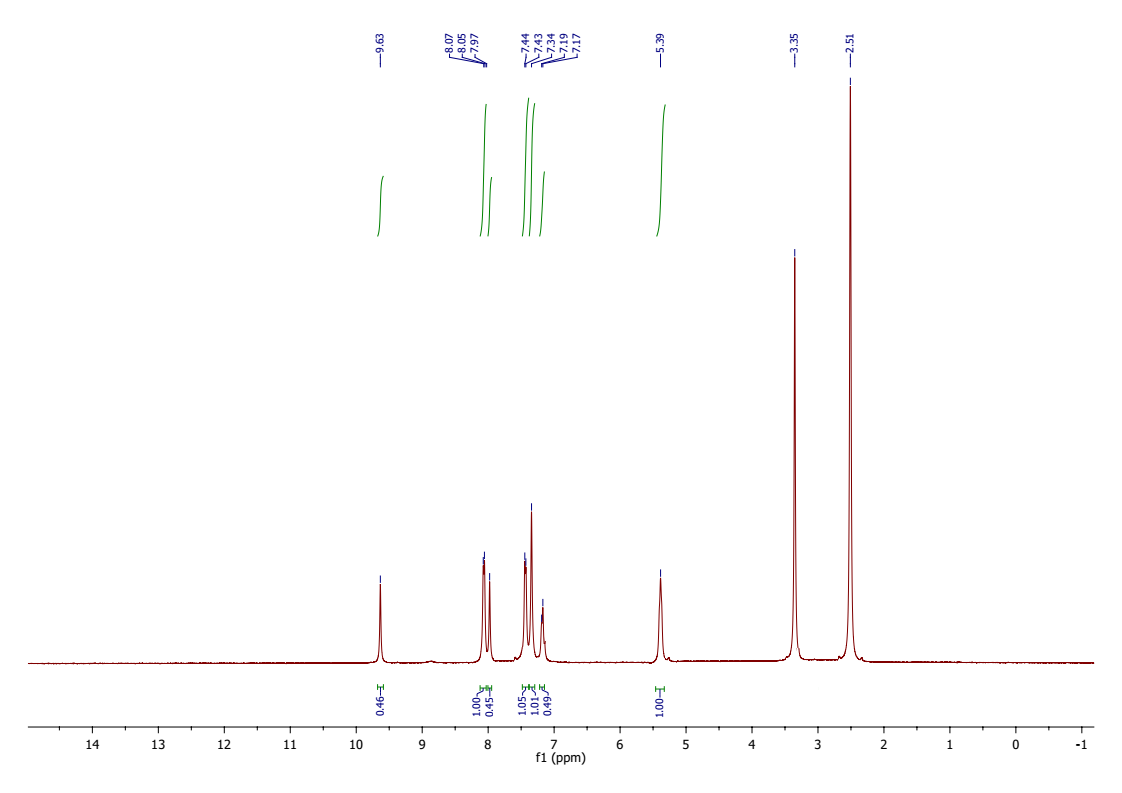
**

^1^H NMR spectra of compound **5j**

**
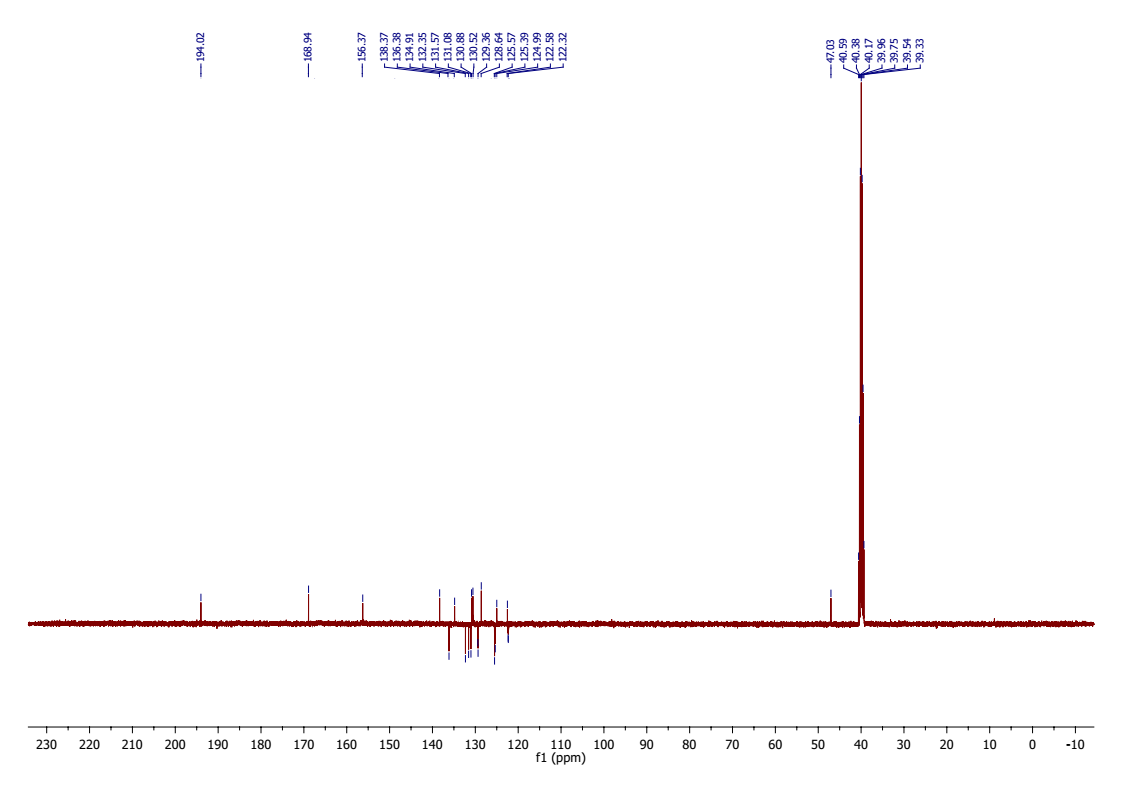
**

^13^C NMR (APT) spectra of compound **5j**

**
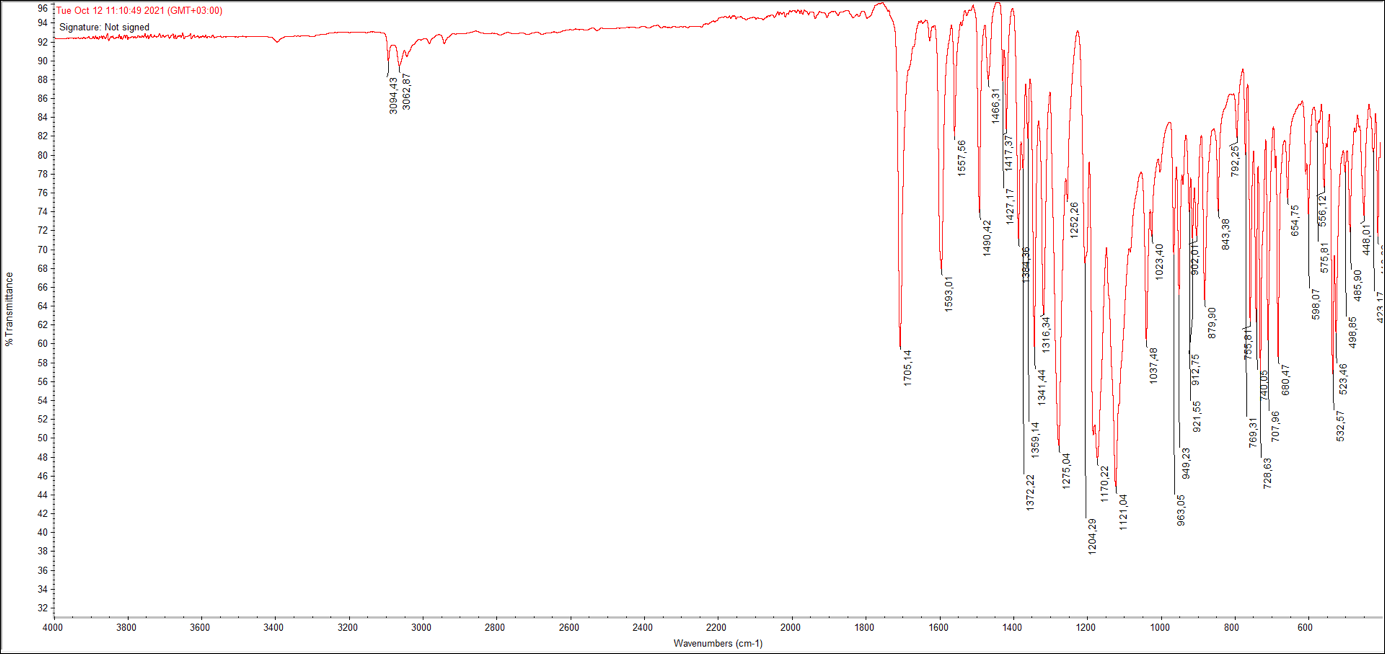
**

FTIR spectra of compound **5k**

**
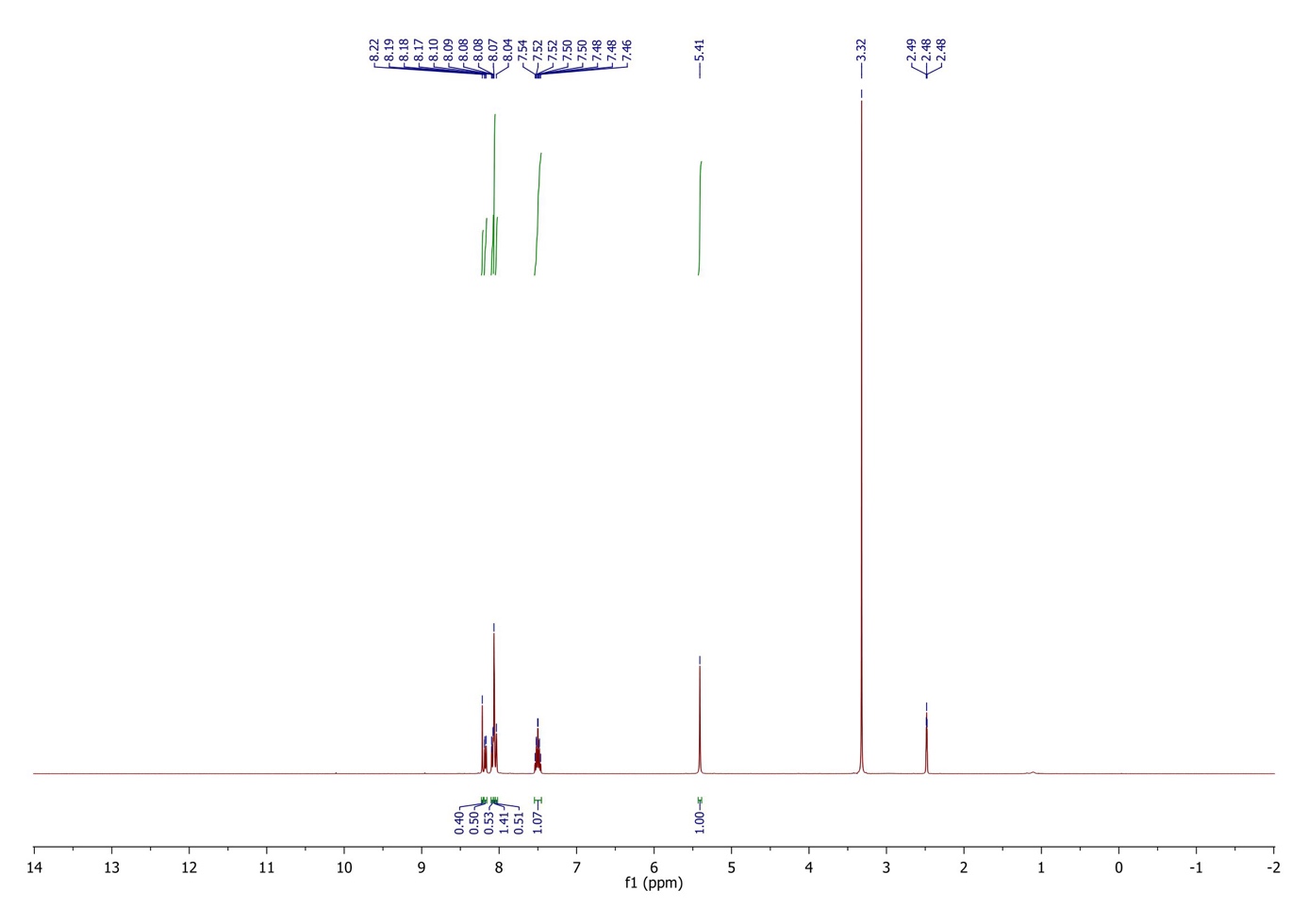
**

^1^H NMR spectra of compound **5k**

**
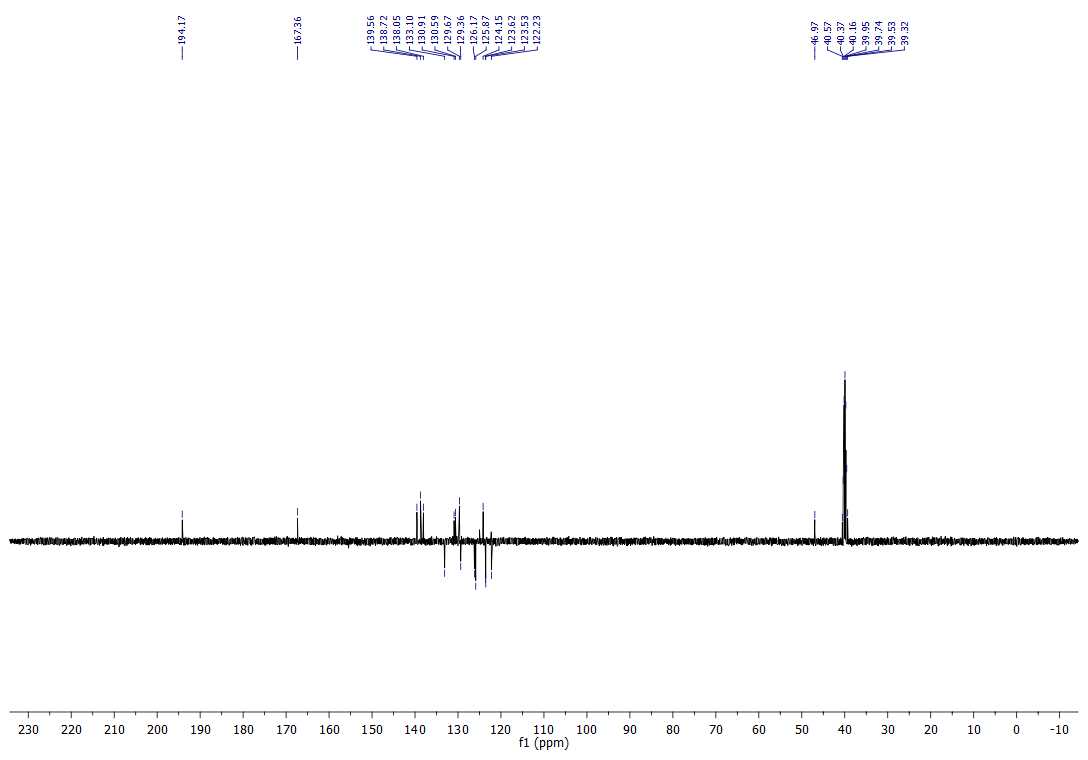
**

^13^C NMR (APT) spectra of compound **5k**

**
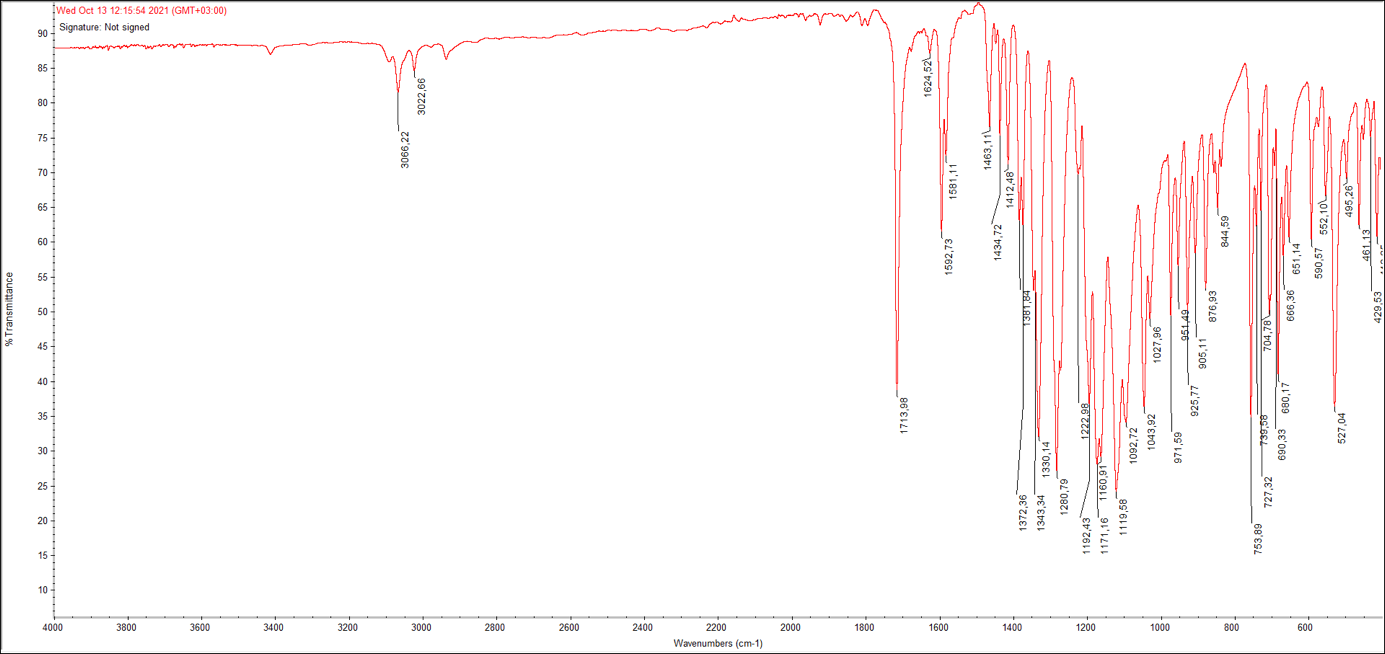
**

FTIR spectra of compound **5l**

**
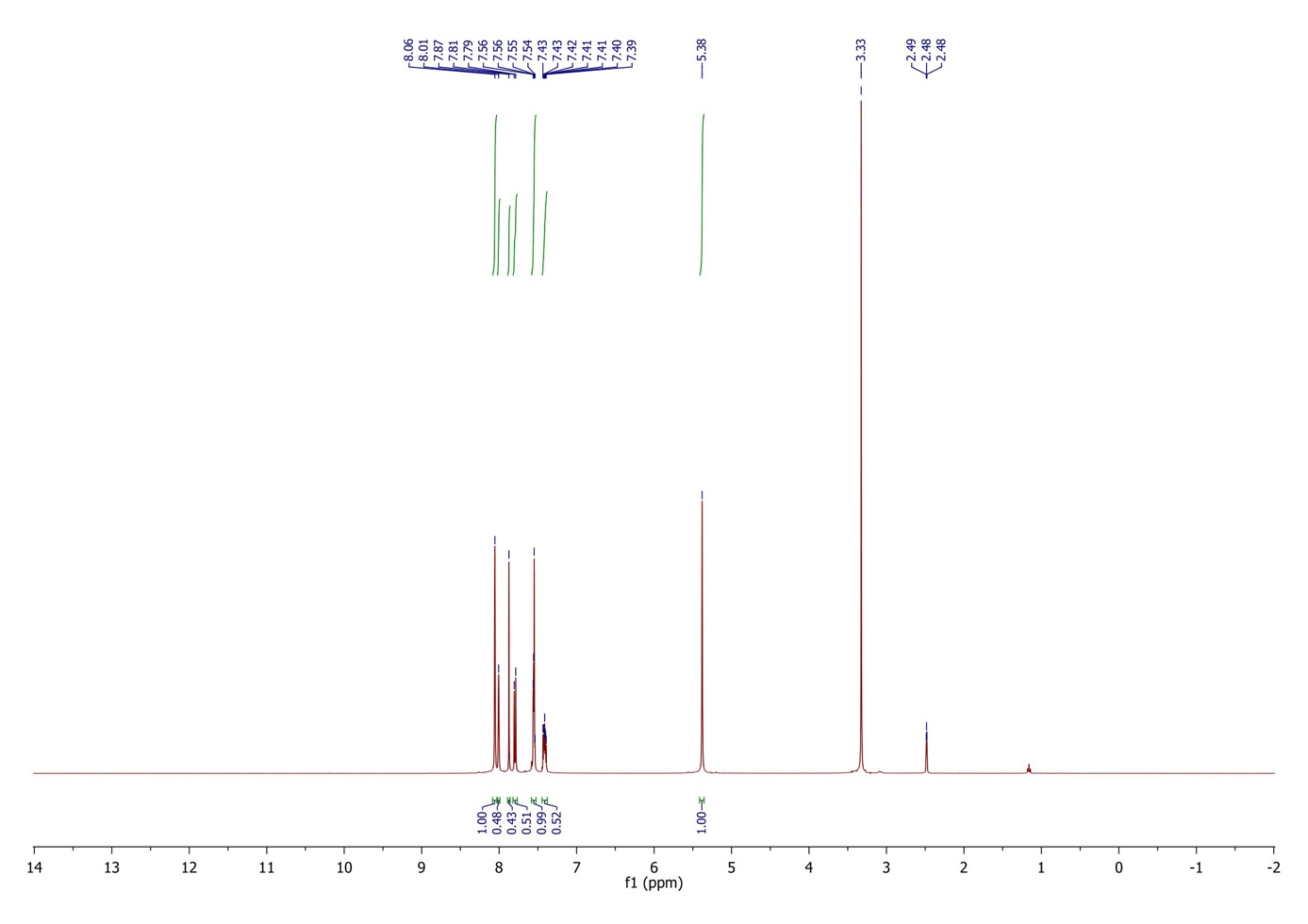
**

^1^H NMR spectra of compound **5l**

**
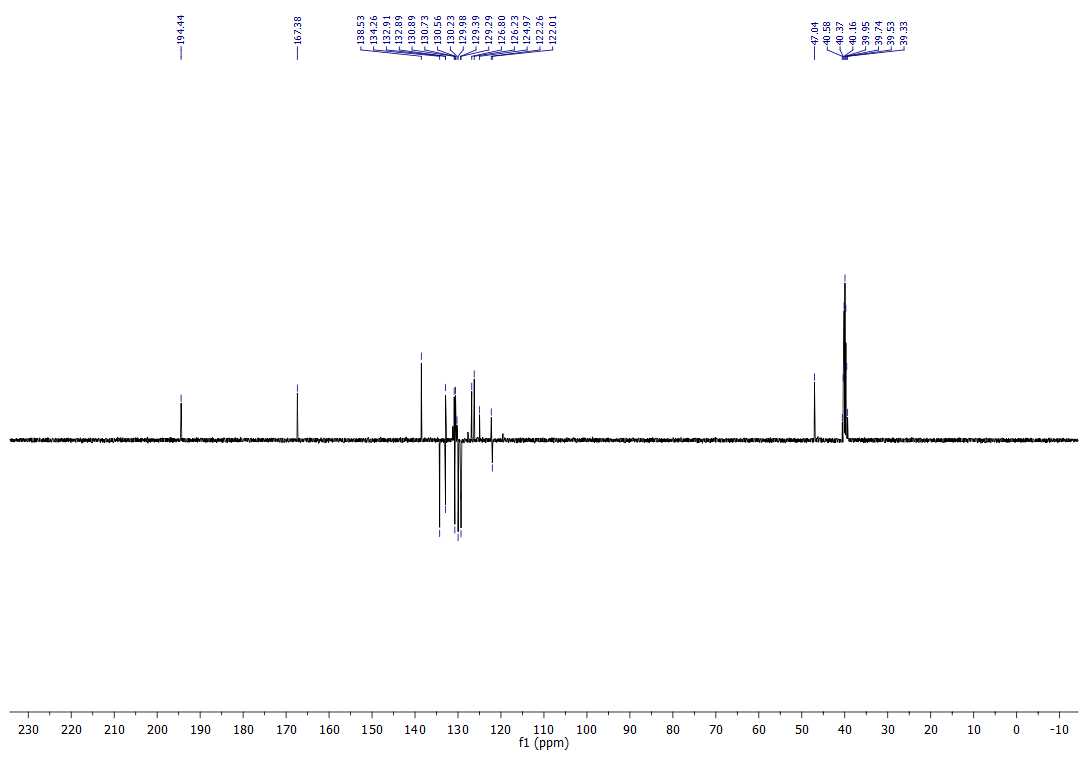
**

^13^C NMR (APT) spectra of compound **5l**

**
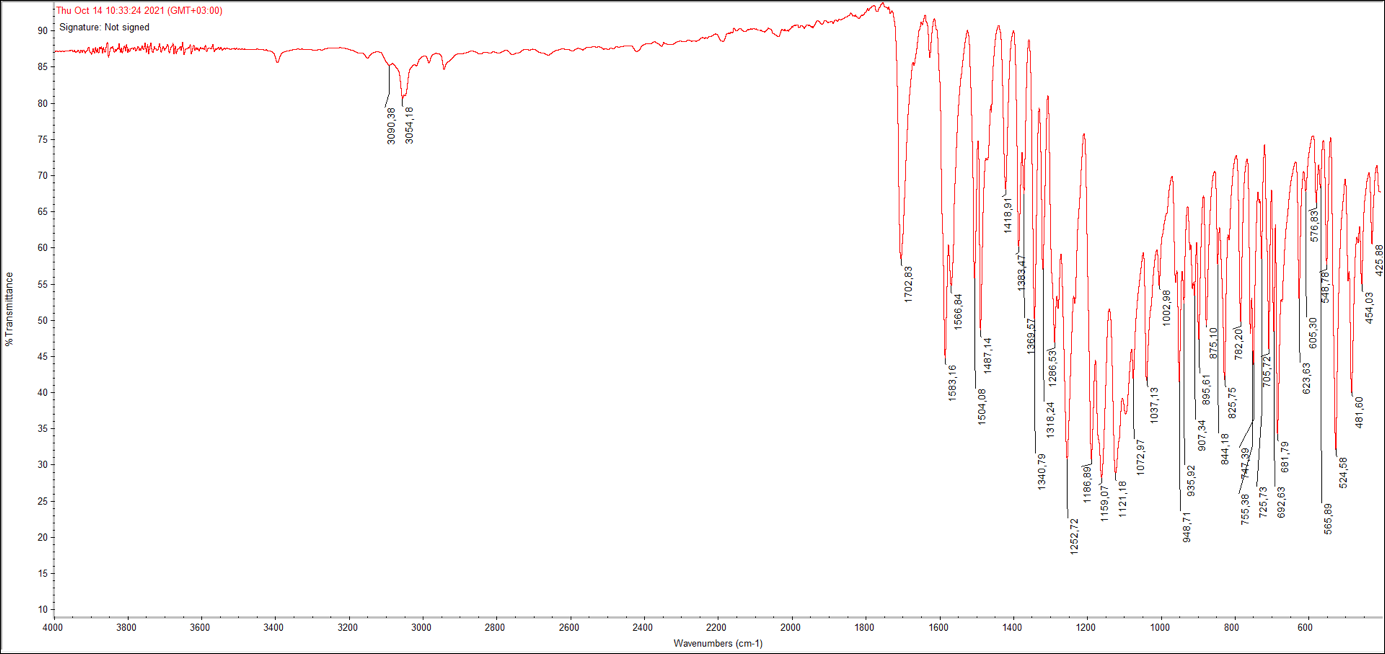
**

FTIR spectra of compound **5m**

**
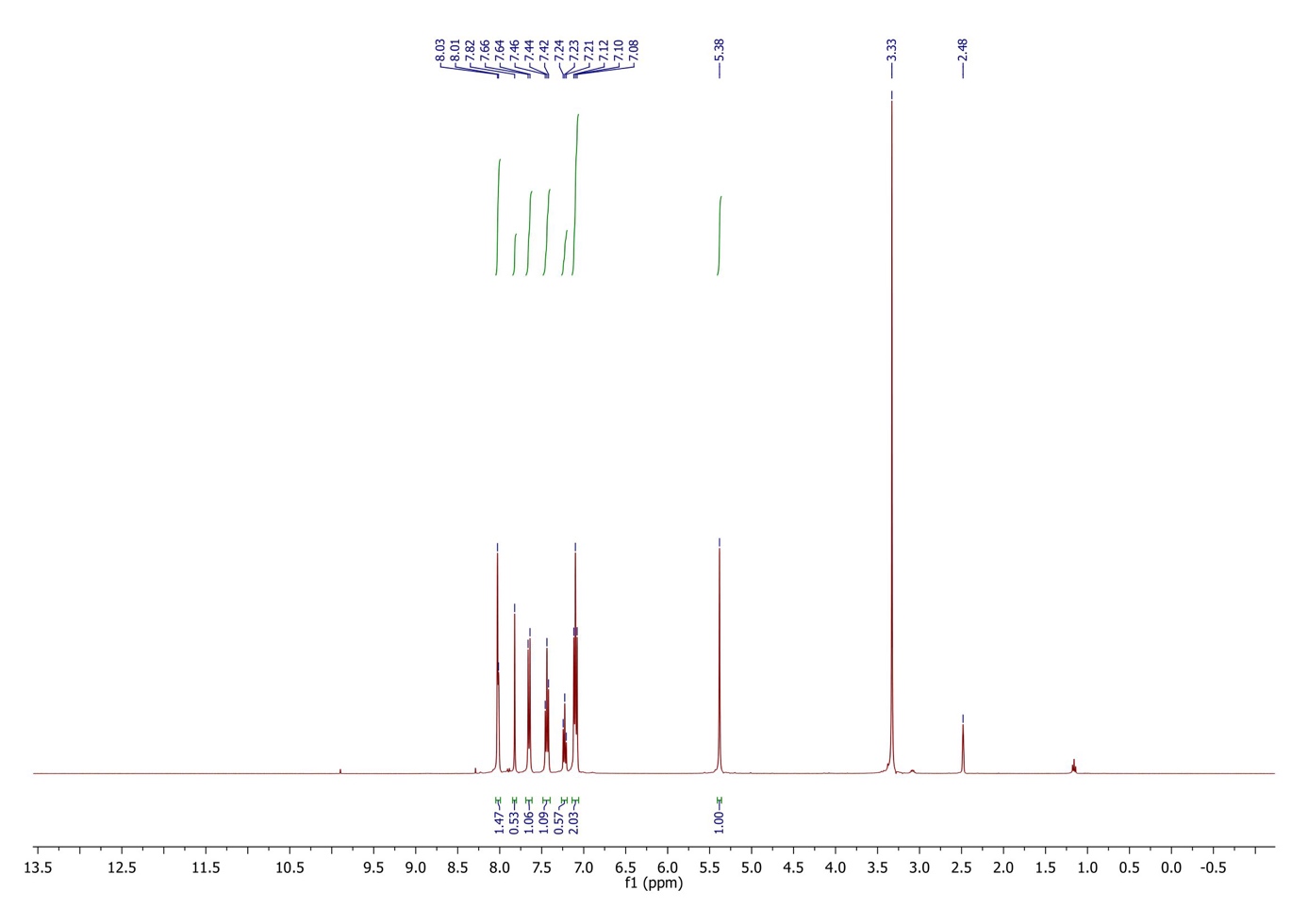
**

^1^H NMR spectra of compound **5m**

**
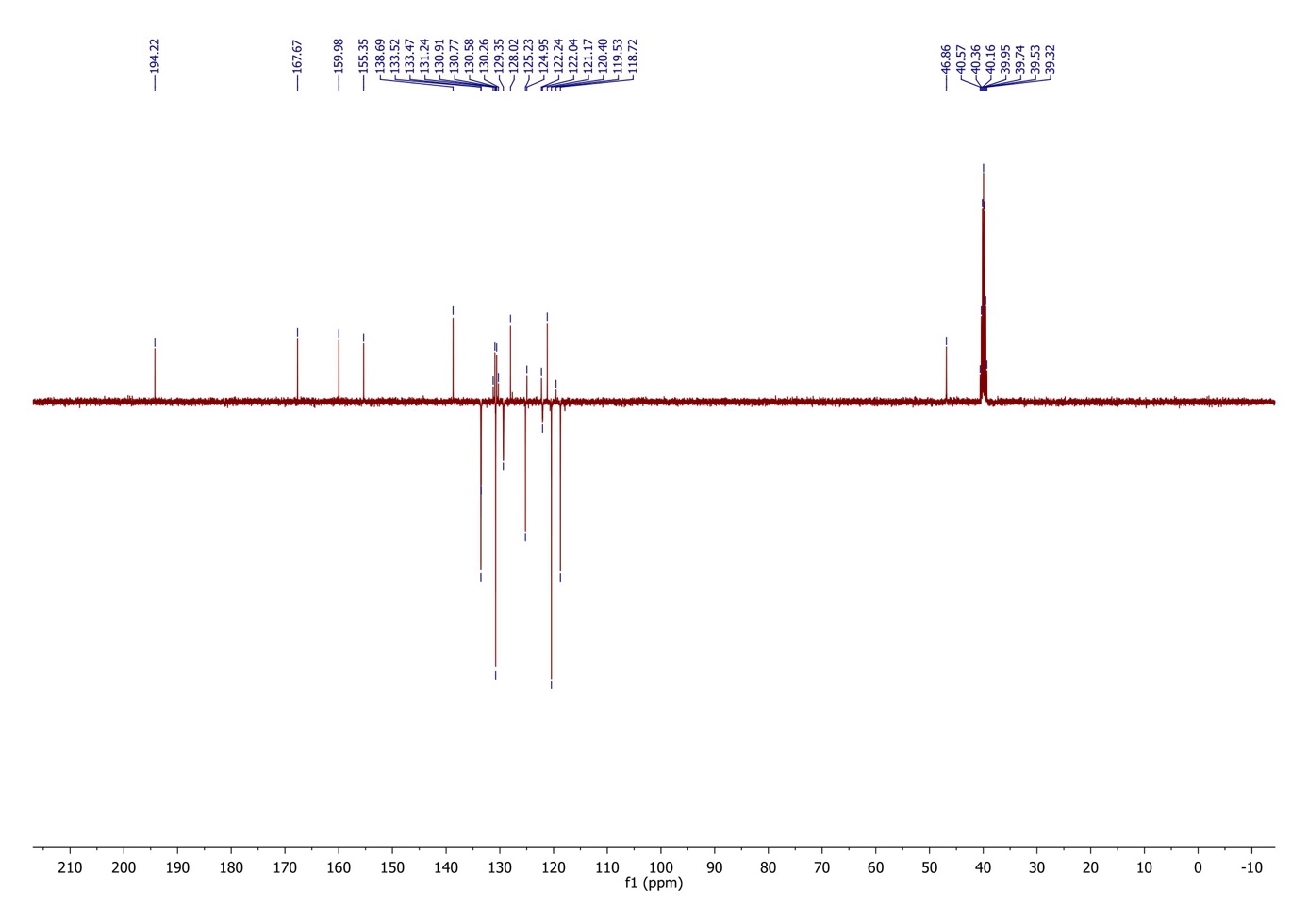
**

^13^C NMR (APT) spectra of compound **5m**

**
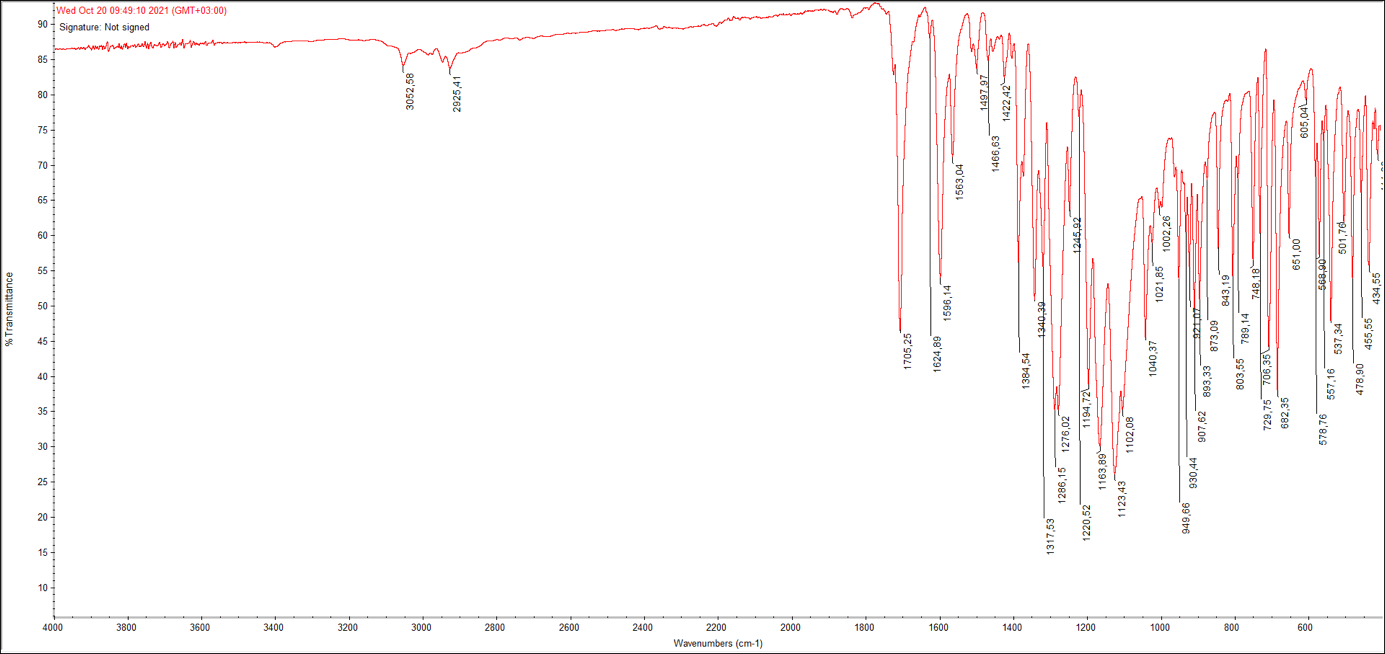
**

FTIR spectra of compound **5n**

**
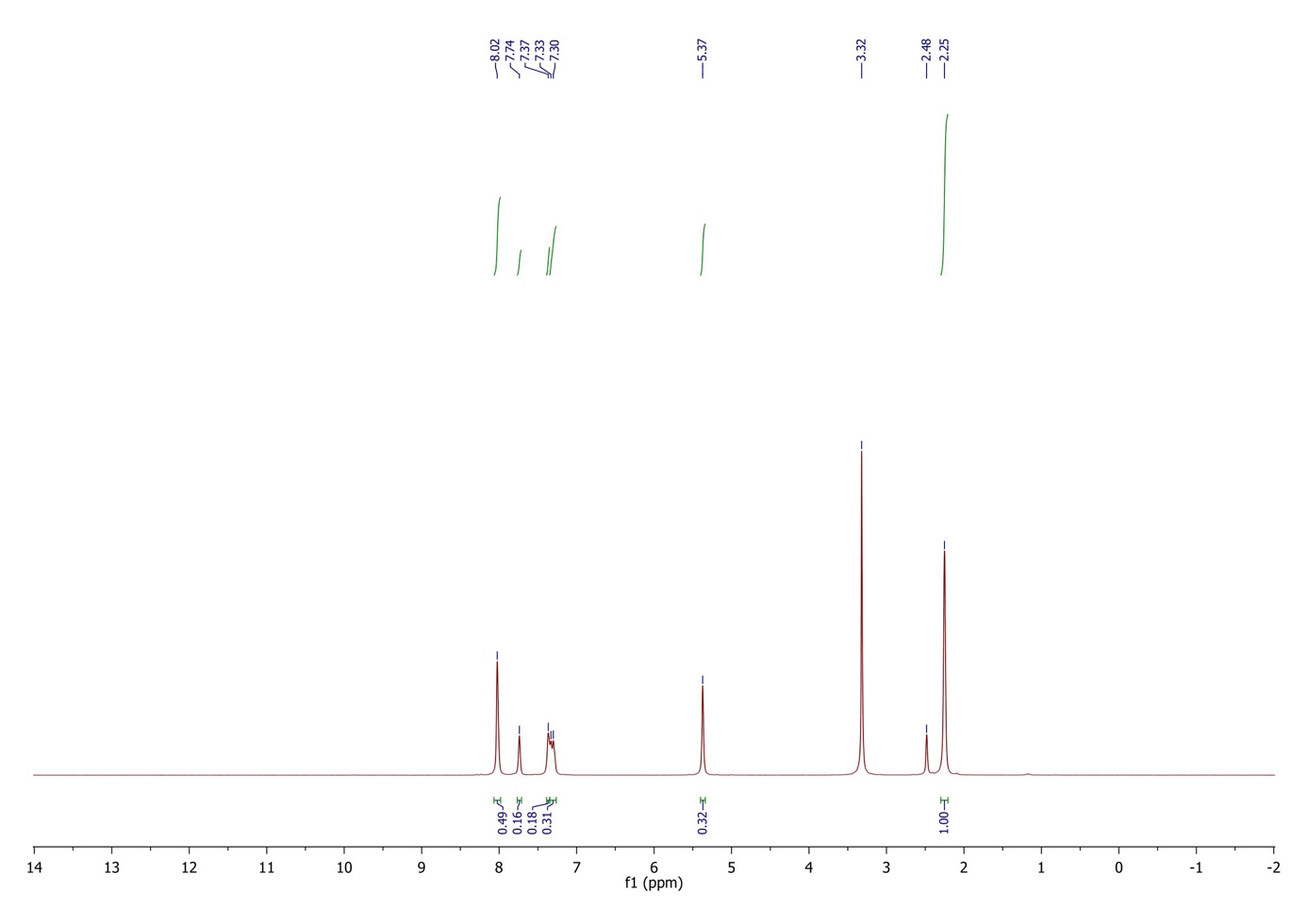
**

^1^H NMR spectra of compound **5n**

**
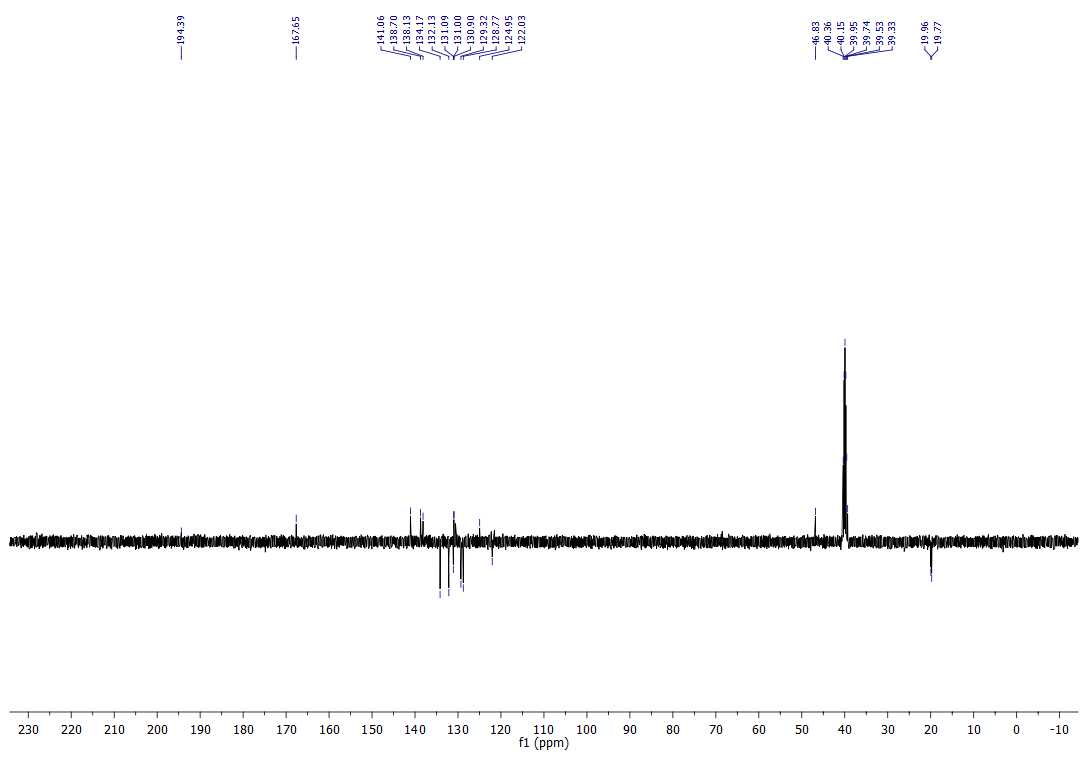
**

^13^C NMR (APT) spectra of compound **5n**
